# Supplementary material for: Temporal Sharpening of Sensory Responses by Layer V in the Mouse Primary Somatosensory Cortex
Source: Curr Biol. 2020 May 4;30(9):1589–1599.e10. doi: 10.1016/j.cub.2020.02.004 (PMC7198976; doi:10.1016/j.cub.2020.02.004)
Supplement: Document S1. Figures S1–S7 and Tables S1–S5 [file mmc1.pdf]

Current Biology, Volume *30*

Supplemental Information

Temporal Sharpening of Sensory Responses

by Layer V in the Mouse

Primary Somatosensory Cortex

Dania Vecchia, Riccardo Beltramo, Fabio Vallone, Ronan Chéreau, Angelo Forli, Manuel Molano-Mazón, Tanika Bawa, Noemi Binini, Claudio Moretti, Anthony Holtmaat, Stefano Panzeri, and Tommaso Fellin

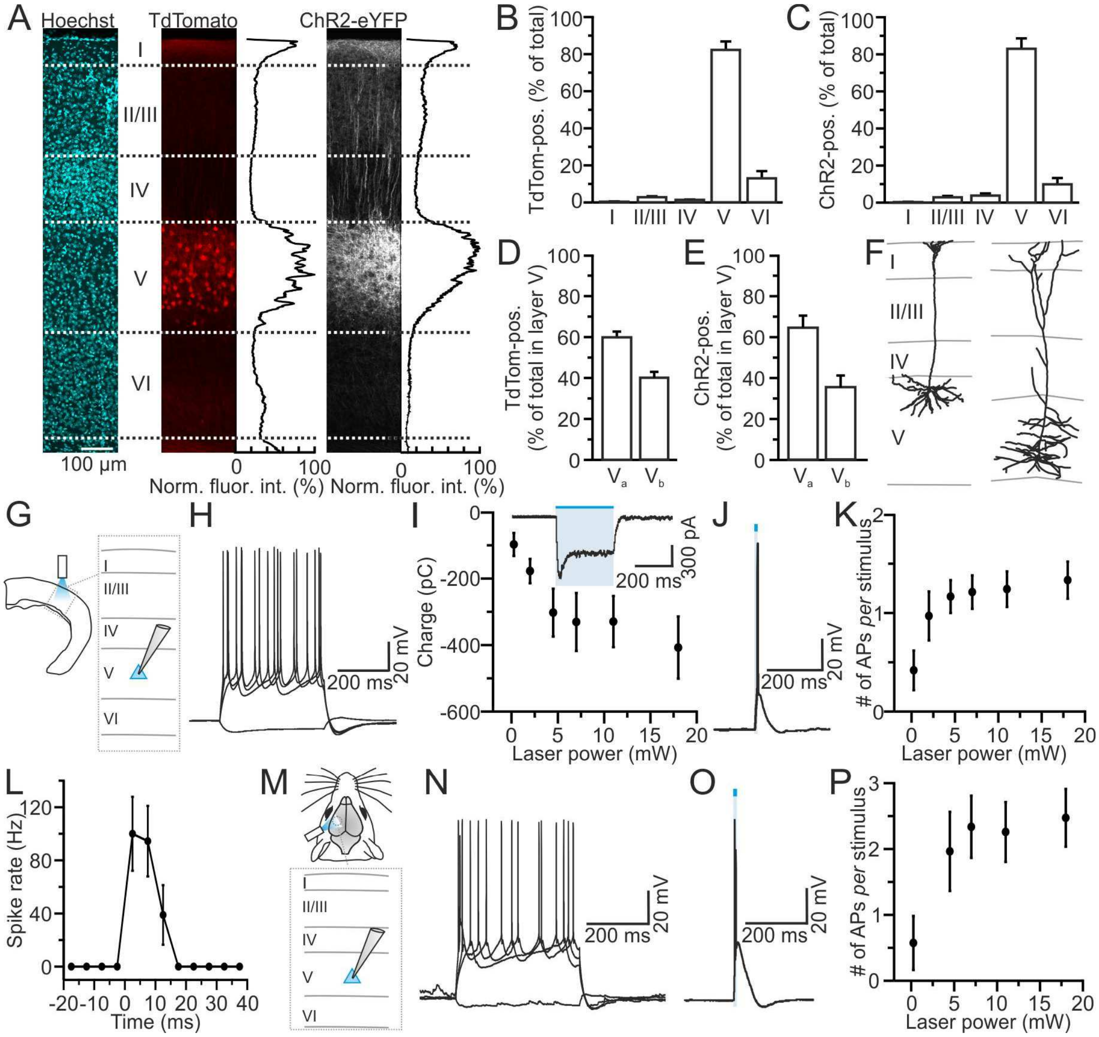

Figure S1. Selective expression of transgenes and functional characterization of ChR2 in layer V principal cells. Related to Figure 1. (A) Confocal images showing a coronal section of the primary somatosensory cortex of a double transgenic mouse *Rbp4-cre* x *TdTomato* which was injected with AAV transducing ChR2-eYFP. Left: The boundaries of cortical layers (dashed lines) were identified on the basis of cellular density using Hoechst staining. Middle and right: confocal images showing TdTomato (middle) and ChR2-eYFP (right) expression. The normalized fluorescence intensity is shown on the side for both images. **(B-C)** Percentage of TdTomato- (B) and ChR2-expressing cells (C) across cortical layers. Data were pooled from 3 animals (3 sections *per* animal). Within layer V, we found that 22 ± 6% and 16 ± 3% of the total Neuronal Nuclei- (NeuN) positive population expressed TdTomato and ChR2, respectively (n = 3 animals, 3 sections *per* animal). **(D-E)** Percentage of TdTomato- (D) and ChR2- (E) expressing cells within layer V_a and layer V_b. **(F)** Examples of ChR2-positive layer V cells that were filled with biocytin and reconstructed morphologically *a posteriori*. A layer V_a slender-tufted pyramidal neuron is shown on the left and a layer V_b thick-tufted pyramidal cell on the right. **(G)** Schematic representation of the experimental configuration used for brain slice experiments. Blue light was delivered to isolated cortical slices through a fiber optic and recordings were performed from ChR2-positive neurons (blue triangle). **(H)** Representative current-clamp patch-clamp recordings showing the membrane potential responses to current injections (-100, +350, +500, +650 pA) of a ChR2-positive layer V neuron. **(I)** Average charge transfer as a function of the light intensity (stimulus duration: 300 ms; n = 7 ChR2-positive cells). Inset: representative voltage-clamp recording showing blue light-induced photocurrent. Photocurrent latency: 0.20 ± 0.03 ms, n = 7 cells. **(J)** Representative current-clamp patch-clamp recording from a ChR2-positive layer V neuron showing the membrane potential response to 10 ms of blue light stimulation. **(K)** Average number of action potentials (AP) *per* stimulus as a function of the light stimulus intensity in ChR2-positive layer V neurons (n = 12 cells). A 10-ms light pulse in ChR2-positive neurons elicited one or few APs on average, over a large range of power values. Since our goal was to probe the cortical responses to a brief activation of layer V pyramidal cells, we used a 10-ms photoactivation stimulus throughout this study. **(L)** Average peri-stimulus time histogram (bin: 5 ms) of layer V ChR2-positive neurons during blue light stimulation (stimulus duration: 10 ms; laser power: 4.6-5 mW), n = 12 cells. **(M)** Schematic of the experimental configuration for *in vivo* recordings. **(N)** Representative current-clamp patch-clamp recording from a ChR2-positive layer V neuron *in vivo* showing membrane

potential responses to current injections (-100, +350, +450, +550 pA). **(O-P)** Same as in (J-K) for ChR2-positive layer V pyramidal neurons *in vivo*. In (P), n = 5 cells from 4 animals. Latency to membrane depolarization: 0.31 ± 0.02 ms, n = 5 cells.

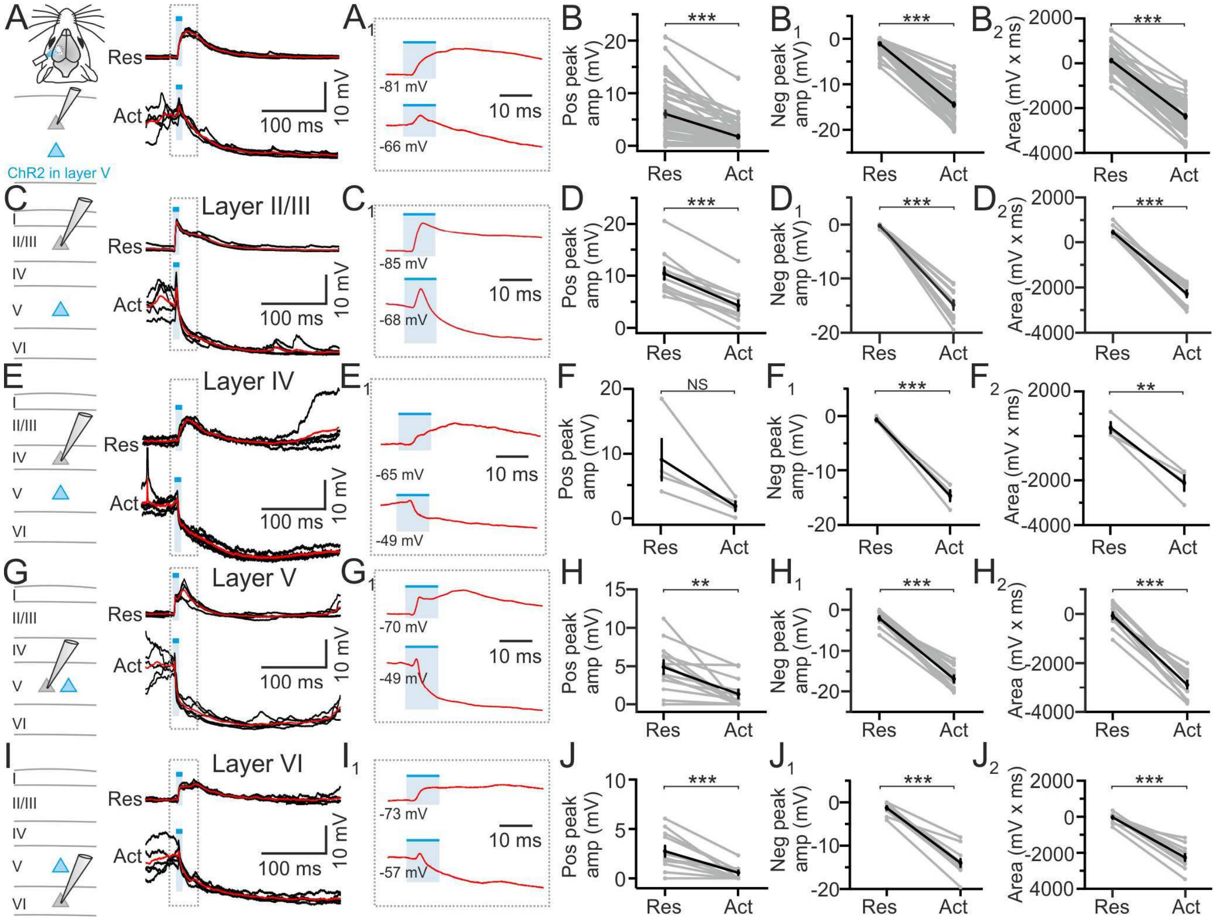

Figure S2. Layer V photostimulation evokes state-dependent changes in the membrane potential of cortical neurons *in vivo*. Related to Figure 1. (A) Left: Schematic of the experimental configuration for intracellular recordings in anesthetized mice. Blue light was delivered through a fiber optic. In this as well as in other figures ChR2-positive neurons are indicated in blue, ChR2-negative cells in grey. Right: Representative patch-clamp current-clamp recordings from a ChR2-negative pyramidal neuron showing cellular responses to blue light (blue bars, 10 ms duration) during spontaneous activity. Responses to five different light stimuli are shown in black and their average in red for light stimuli during the resting (Res, top) and the activated state (Act, bottom). **(A_1)** Zoom in of the average response (red) highlighted in (A, grey dotted line). **(B-B_2)** Positive peak amplitude (B), negative peak amplitude (B_1), and integral (Area, B_2) of the light-evoked response in ChR2-negative principal neurons in the resting (Res) and activated (Act) states. In this as well as in other figures, values from individual experiments are shown in grey, the average of all cells in black. n = 55 cells from 30 animals; Wilcoxon test in (B-B_1); Paired Student's *t*-test in (B_2). Subthreshold latency: 2.90 ± 0.09 (Res) and 2.37 ± 0.10 (Act). **(C-D_2)** Same as in (A-B_2) for ChR2-negative layer II/III principal neurons. n = 12 cells from 6 animals; Paired Student's *t*-test. **(E-F_2)** Same as in (A-B_2) for ChR2-negative layer IV principal neurons. n = 4 cells from 4 animals; Paired Student's *t*-test. **(G-H_2)** Same as in (A-B_2) for ChR2-negative layer V principal neurons. n = 13 cells from 8 animals; Paired Student's *t*-test. **(I-J_2)** Same as in (A-B_2) for ChR2-negative layer VI principal neurons. n = 12 cells from 9 animals. Subthreshold latencies for cells across layers are reported in Table S2.

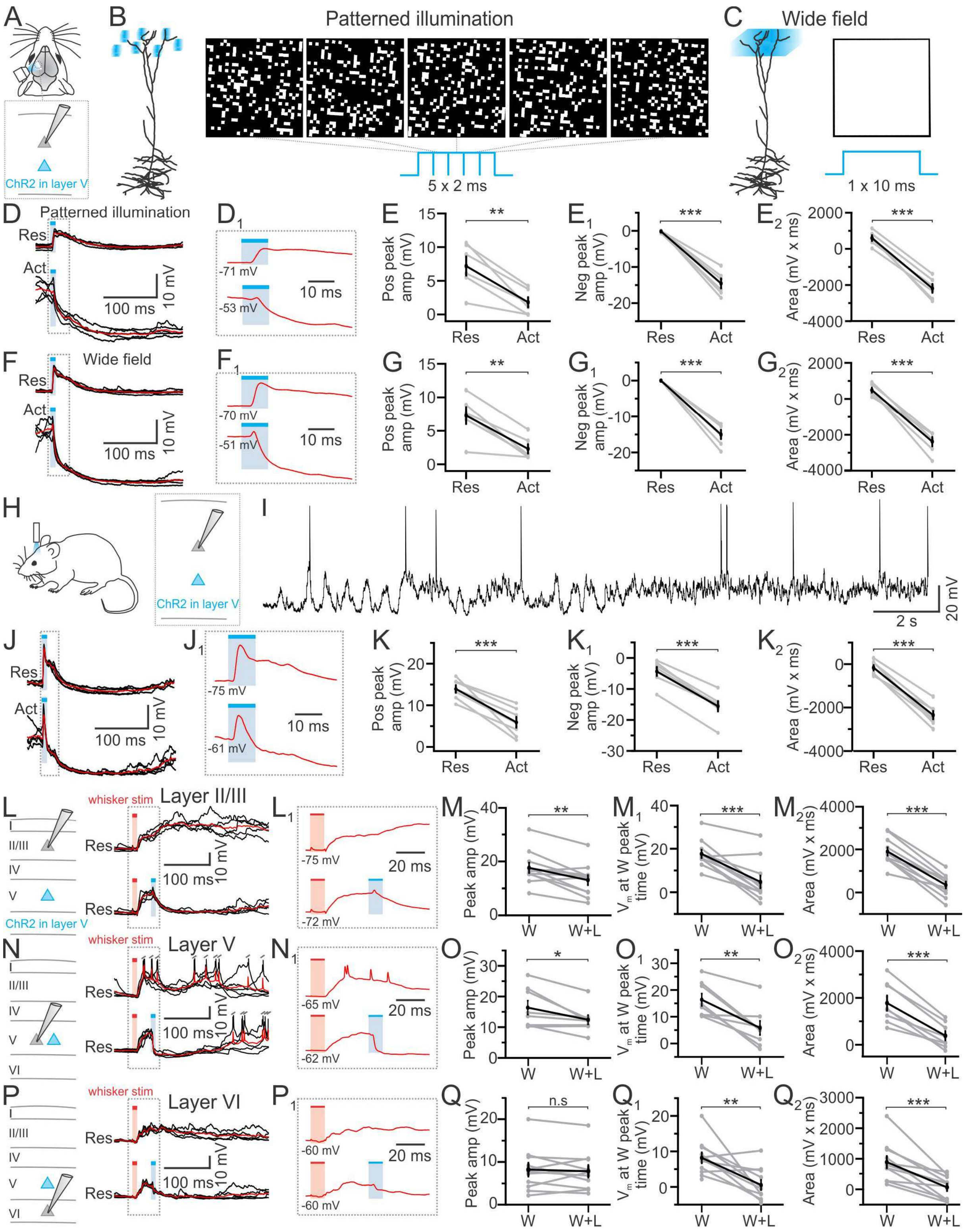

Figure S3. Stimulation of layer V pyramidal neurons either with patterned illumination or in awake mice generates state-dependent responses in cortical neurons. Related to Figure 1. (A) Schematic representation of the experimental configuration for patterned illumination in anesthetized animals. Fiber optic-mediated wide-field illumination may lead to non-physiological activation of ChR2-positive neurons. Because the fiber optic used for the photostimulation in Figures 1A-F and Figure S2 was placed on the pial surface, and because blue light is strongly scattered and absorbed within the brain, the neuronal structures that were illuminated most strongly under our experimental conditions were the distal

dendrites of layer V neurons. The photostimulation would thus likely cause synchronous depolarization of large portions of the dendritic trees. In contrast, under physiological conditions, cortical pyramidal neurons are bombarded by bursts of depolarizing synaptic inputs, each spatially confined. To mimic this spatially restricted pattern of dendritic activation *in vivo*, we used an optical illumination system based on a digital micromirror device (DMD; see STAR Methods). We projected light patterns of arbitrary geometry on the cortical surface, at a fast refresh rate (500 Hz). We selected a 400 x 400 μm^2 or 200 x 200 μm^2 field of view and we illuminated it with a spatially randomized patterns of 12-μm light spots (patterned stimulation, see STAR Methods for details). **(B)** The dendritic tree of layer V neurons was illuminated *in vivo* with complex light patterns (patterned illumination) to mimic physiological depolarization generated by local dendritic inputs. Five consecutive patterns of randomly generated spots (black and white images on the right) were projected for 2 ms each. White pixels indicate regions illuminated by blue light, black pixels regions that were not illuminated. **(C)** In each patterned illumination experiments, wide field illumination was also performed as control. **(D)** Representative current-clamp recordings from a ChR2-negative principal neuron *in vivo* during patterned optogenetic activation of layer V. Responses to five different trials are shown in black and their average in red for stimuli that occurred during the resting (Res, top) and activated state (Act, bottom). **(D_1)** The portion of the average response highlighted in (D, grey dotted line) is displayed at an enlarged scale. **(E-E_2)** Positive peak amplitude (E), negative peak amplitude (E_1), and area (E_2) of the light-evoked membrane response in ChR2-negative principal neurons in the resting (Res) and activated (Act) states following patterned illumination. n = 6 cells from 3 animals; Paired Student's *t*-test. **(F-G_2)** Same as in D-E_2 for wide field illumination. n = 6 cells from 3 animals; Paired Student's *t*-test. Wide-field illumination obtained with either the fiber optic (Figures S2A-B_2) or the DMD, which precisely controlled the area of illumination (400 x 400 μm^2 or 200 x 200 μm^2), produced similar responses on cortical neurons. Subthreshold latencies are reported in Table S2. **(H-I)** Schematic representation of the experimental configuration for intracellular recordings in awake mice (H) and example of continuous recording from a ChR2-negative neurons under these experimental conditions (I). **(J-J_1)** Representative current-clamp recording from a ChR2-negative principal neuron in an awake mouse. Five superimposed responses (black), and their average (red) are shown for wide field stimuli occurring in the resting (top) and activated state (bottom). **(K-K_2)** Same as in G-G_2 in awake mice. n = 7 cells from 3 animals; Paired Student's *t*-test. Subthreshold latencies are displayed in Table S2. **(L)** Left: schematic representation of the experimental configuration. A single whisker (C1, C2, or D1) was deflected with a piezoelectric actuator (stimulus duration: 10 ms) while patch-clamp current-clamp recordings were performed in the correspondent barrel related column in anesthetized animals (which was identified using intrinsic optical imaging, see STAR Methods for more details). Electrophysiological recordings were combined with optogenetic layer V activation (blue light duration: 10 ms). Right: representative traces from a layer II/III ChR2-negative neuron *in vivo*. Cellular responses to whisker deflection (red bar) and to whisker deflection followed by optogenetic activation of layer V pyramidal cells (blue bar) are shown. **(L_1)** The average responses highlighted in (L) are displayed at an enlarged scale. **(M-M_2)** Positive peak amplitude (M), membrane potential value at the whisker-response time peak (M_1) and area (M_2) in ChR2-negative principal layer II/III cells under the different experimental conditions. n = 11 cells from 9 animals; Paired Student's *t*-test. **(N-O_2)** Same as in (L-M_2) for identified ChR2-negative layer V principal neurons. n = 8 cells from 7 animals; Paired Student's *t*-test. **(P-Q_2)** Same as in (L-M_2) for identified ChR2-negative layer VI principal neurons. n = 11 cells from 9 animals; Paired Student's *t*-test.

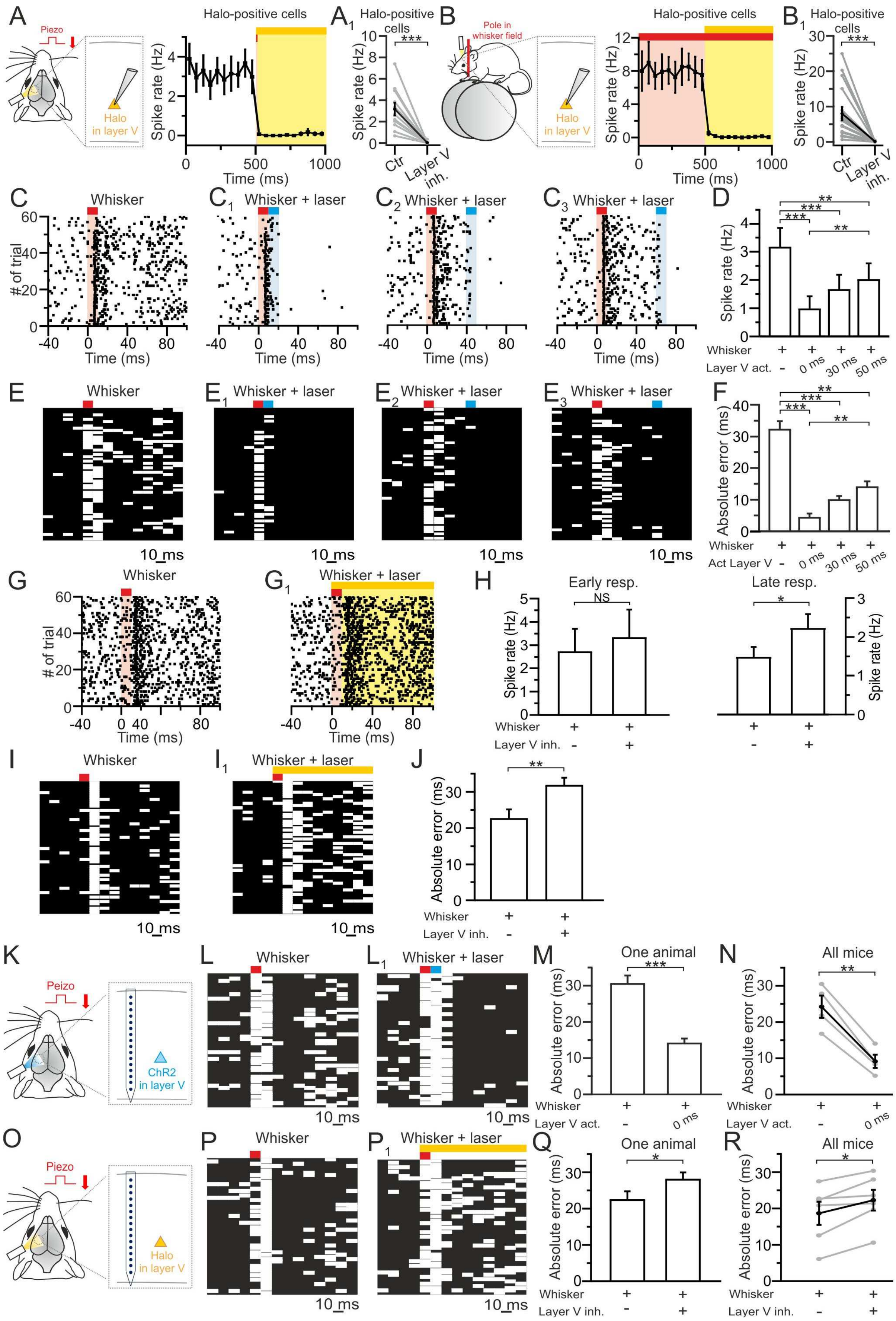

A
Piezo
Halo in layer V
Halo-positive cells
Spike rate (Hz)
Time (ms)
A1
Halo-positive cells
***
Ctr
Layer V inh.
B
Pole in whisker field
Halo in layer V
Halo-positive cells
Spike rate (Hz)
Time (ms)
B1
Halo-positive cells
***
Ctr
Layer V inh.
C
Whisker
# of trial
Time (ms)
C1
Whisker + laser
C2
Whisker + laser
C3
Whisker + laser
D
Spike rate (Hz)
Whisker
Layer V act.
0 ms
30 ms
50 ms
E
Whisker
E1
Whisker + laser
E2
Whisker + laser
E3
Whisker + laser
10 ms
F
Absolute error (ms)
Whisker
Act Layer V
0 ms
30 ms
50 ms
G
Whisker
# of trial
Time (ms)
G1
Whisker + laser
H
Early resp.
NS
Late resp.
Spike rate (Hz)
Whisker
Layer V inh.
I
Whisker
I1
Whisker + laser
10 ms
J
Absolute error (ms)
Whisker
Layer V inh.
K
Peizo
ChR2 in layer V
L
Whisker
L1
Whisker + laser
10 ms
M
One animal
Absolute error (ms)
Whisker
Layer V act.
0 ms
N
All mice
Absolute error (ms)
Whisker
Layer V act.
0 ms
O
Piezo
Halo in layer V
P
Whisker
P1
Whisker + laser
10 ms
Q
One animal
Absolute error (ms)
Whisker
Layer V inh.
R
All mice
Absolute error (ms)
Whisker
Layer V inh.

Figure S4. Layer V sharpens the temporal profile of neuronal responses to whisker stimulation across cortical layers in individually recorded cells and in population recordings. Related to Figures 1-3. (A) Left: schematic representation of the experimental configuration in anesthetized mice. Recordings were performed from Halo-positive neurons. Optogenetic and whisker stimulations were performed as in Figure 1G. Right: peri-stimulus time histogram (bin: 50 ms) of Halo-positive cells under control conditions and after simultaneous whisker stimulation (red bar, stimulus duration: 10 ms) and layer V photoinhibition (yellow bar, stimulus duration: 500 ms). Juxtasomal recordings were performed as described in Figure S3L. n = 11 cells from 7 animals. Please note that 20 Halo-positive neurons out of 90 (22 %) were recorded in anesthetized animals (see STAR Methods). The plot in (A) shows the 11 cells in which whisker stimulation and light stimulation were performed at the same time. In the remaining 9 Halo-positive cells, whisker stimulation was performed either after light illumination or not at all. **(A_1)** Average spike rate under the different experimental conditions (Control, Ctrl, *vs* layer V inhibition, layer V inh.). n = 11 cells from 7 animals; Wilcoxon test. **(B)** Left: same as in (A) for experiments in awake mice actively whisking on a pole, which was lowered in the contralateral mouse whisker field. Optogenetic and whisker stimulations were performed as in Figure 3. Right: peri-stimulus time histogram (bin 50 ms) of Halo-positive cells during pole presentation (red bar) and simultaneous pole presentation and layer V photoinhibition (yellow bar, stimulus duration 500 ms). n = 16 cells from 11 animals. 16 Halo-positive neurons out of 64 (25 %) were recorded in awake animals (see STAR Methods). **(B_1)** Same as in (A_1) for awake mice. n = 16 cells from 11 animals; Paired Student's *t*-test. **(C-C_3)** Pseudo-simultaneous population response to whisker stimulation obtained pooling together whisker responsive and whisker nonresponsive sequentially-recorded deep neurons that were negative for ChR2. Juxtasomal recordings were performed as described in Figure S3L. Neural activity was recorded in response to vibrissae stimulation (red bar), in the absence (C) or presence of layer V photostimulation (blue bar, C_1-C_3). Blue light was presented at different delays (~ 0 ms in C_1, ~ 30 ms in C_2, ~ 50 ms in C_3) with respect to the time of onset of the whisker-evoked response. n = 27 cells from 13 animals. **(D)** Average cellular spike rate under the different experimental conditions. n = 27 cells from 13 animals; Friedman test. **(E-E_3)** PREs from all whisker responsive and whisker non-responsive cortical neurons (and all trials) during whisker stimulation (red bar, E) and whisker stimulation followed by layer V optogenetic activation (blue bar, E_1-E_3). **(F)** Average absolute error of PRE under the different experimental conditions. All PREs in the time window [-40 , +100] ms from the onset of the whisker stimulus were considered. n = 142 (no layer V activation), n = 42 (layer V activation at ~ 0 ms), n = 72 (layer V activation at ~ 30 ms) and n = 78 (layer V activation at ~ 50 ms) events for n = 27 neurons; Kruskal-Wallis test. **(G-G_1)** Pseudo-simultaneous population response to vibrissae stimulation (red bar) obtained pooling together whisker responsive and whisker non-responsive sequentially-recorded deep neurons (Halo-negative), in the absence (G) or presence of layer V optogenetic inactivation (yellow bar, G_1). Juxtasomal recordings were performed as in (C). **(H)** Same as in (D) for layer V photoinhibition during the early [0, 40] ms and late [40, 100] ms response time window. n = 70 neurons from 19 animals; Wilcoxon test. **(I-I_1)** PREs from all recorded cortical neurons (and all trials) during whisker stimulation (red bar, I) and whisker stimulation paired with layer V optogenetic inactivation (yellow bar, I_1). **(J)** Same as in (F) for layer V optogenetic suppression experiments. n = 177 (no layer V inactivation) and n = 183 (layer V inactivation) events for n = 70 neurons; Mann-Whitney test. **(K)** Schematic representation of the experimental configuration. Optogenetic and whisker stimulations were performed as in Figure 1. A 16-channels linear probe placed across cortical layers was used for recordings. **(L-L_1)** PREs from all simultaneous units and trials recorded in one animal during whisker stimulation (red bar, L) and during whisker stimulation followed by layer V photoactivation (blue bar, L_1). Blue light was presented at ~ 0 ms with respect to the time of onset of the whisker-evoked response. All PREs in a time window from -40 ms to +100 ms from the onset of the whisker stimulation were considered. **(M)** Mean values of absolute error of PREs in whisker stimulation trials and in trials in which whisker stimulation was paired with layer V photostimulation. n = 167 PREs (no layer V activation) and n = 161 PREs (layer V activation at ~ 0 ms); Mann-Whitney test. **(N)** Average values of the absolute error of PREs across animals for whisker stimulation and for concurrent whisker stimulation and layer V photoactivation. n = 4 mice, Paired Student's *t*-test. **(O)** Same as in (K) for layer V photoinhibition. **(P-P_1)** Same as in (L-L_1) for layer V photosupression. **(Q)** Same as in (M) for layer V photoinhibition. n = 132 PREs (no layer V inactivation), n = 190 PREs (layer V inactivation); Mann-Whitney test. **(R)** Same as in (N) for layer V photosuppression. n = 6 mice, Paired Student's *t*-test.

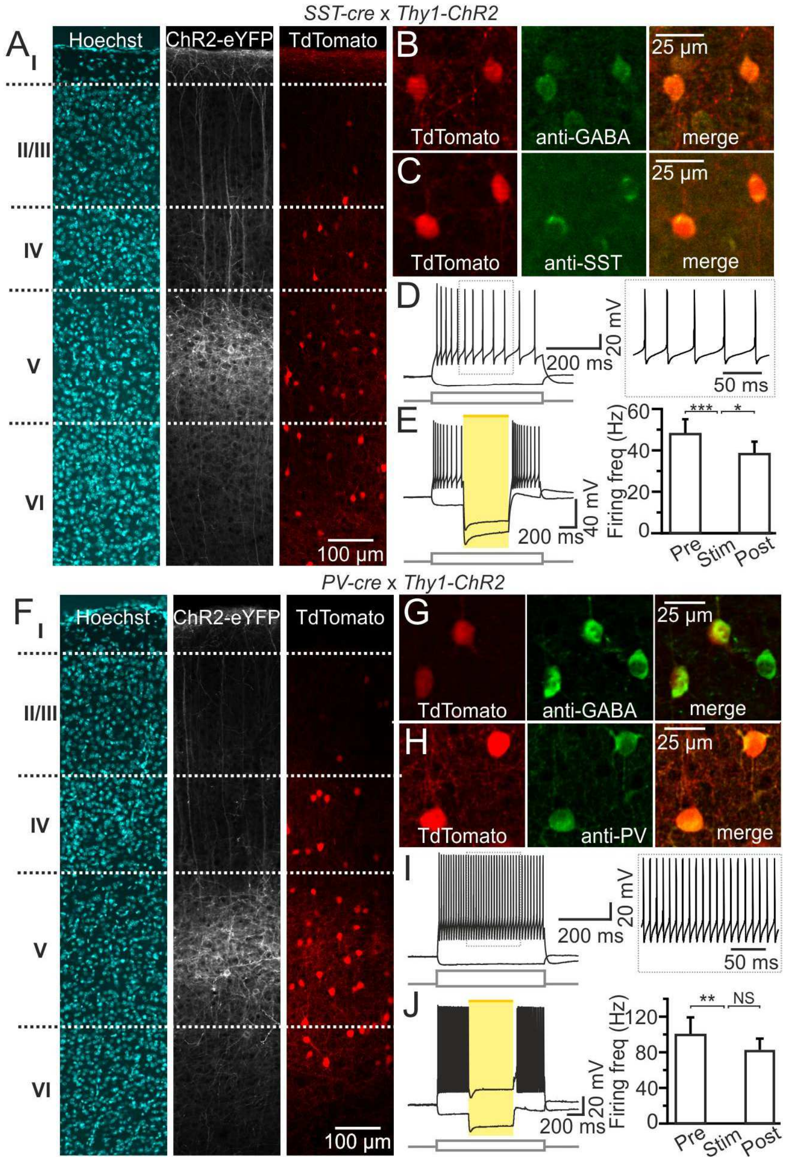

Figure S5. Histological and functional characterization of injected double transgenic *SST-cre* x *Thy1-ChR2* mice and *PV-cre* x *Thy1-ChR2* mice. Related to Figure 7. (A) Confocal images showing a coronal section of the primary somatosensory cortex of a double transgenic mouse *SST-cre* x *Thy1-ChR2* which was injected with AAV transducing TdTomato. Left: the boundaries of cortical layers (dashed lines) were identified on the basis of cellular density using Hoechst staining. Middle and right: Confocal images showing ChR2-eYFP (middle) and TdTomato (right) expression. Note that the somata of ChR2-expressing cells are confined to infragranular layers while TdTomato-positive cells are distributed across cortical layers. Data were pooled from 3 animals (3 sections *per* animal). **(B)** Left: confocal image of cortical section from a *SST-cre* x *Thy1-ChR2* mouse which was injected with AAV transducing TdTomato. Middle: the section was immunostained against GABA (anti-GABA). Right: the two images on the left are shown merged. **(C)** Same as in (B) for a section immunostained against somatostatin (anti-SST). **(D)** *In vitro* current-clamp recordings from a TdTomato-positive cell in a *SST-cre* x *Thy1-ChR2* mouse which was injected with AAVs coding for TdTomato and Arch. Current injections: -100, +250 pA. **(E)** Left: recording from the same neuron as in (D) during concurrent current injections (-100, +250 pA) and Arch photoactivation (yellow bar). Right: average firing frequency before (pre), during (stim), and after (post) yellow light stimulation in cells expressing the inhibitory opsin Arch or Halo. n = 11 cells located in layer V and II/III. Friedman test **(F)** Same as in (A) for a coronal section of the primary somatosensory cortex of a double transgenic mouse *PV-cre* x *Thy1-ChR2* which was injected with AAV transducing TdTomato. Data were pooled from 3 animals (3 sections *per* animal). **(G)** Same as in (B) for a cortical section from a *PV-cre* x *Thy1-ChR2* mouse which was injected with AAV transducing TdTomato. **(H)** Same as in (C) for a section which was immunostained against parvalbumin (anti-PV). **(I)** *In vitro* current-clamp recordings from a TdTomato-positive cell in a *PV-cre* x *Thy1-ChR2* mouse which was injected with AAVs coding for TdTomato and Arch. Current injections: -100, +600 pA. **(J)** Left:

recording from the same neuron as in (I) during concurrent current injections (-100, +450 pA) and Arch photoactivation (yellow bar). Right: average firing frequency before (pre), during (stim), and after (post) yellow light stimulation in cells expressing Arch or Halo. n = 6 cells located in either layer V or II/III. Friedman test.

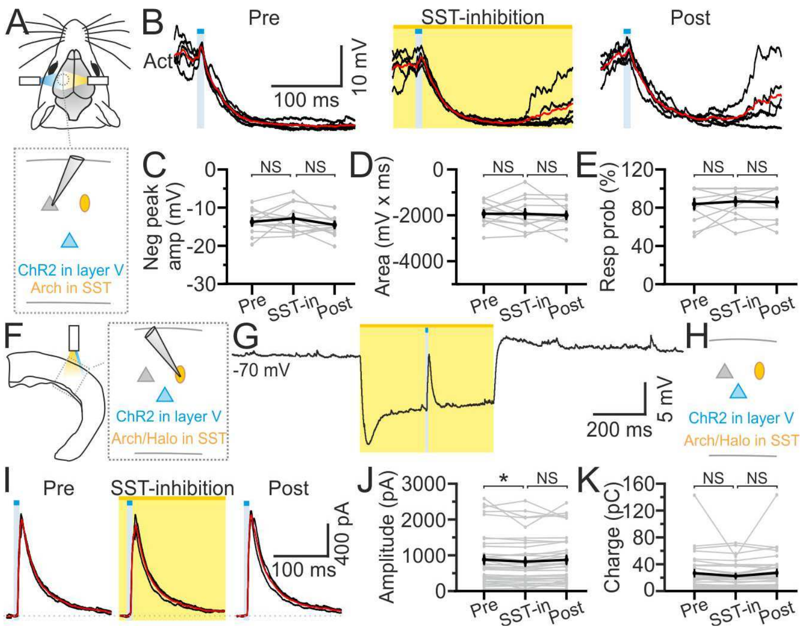

Figure S6. Inhibition of somatostatin (SST) interneurons has minor effects on cortical responses to layer V stimulation. Related to Figure 7. (A) *In vivo* recordings from both superficial and deep principal ChR2-negative cells (grey triangle) during concurrent stimulation of layer V principal neurons (blue triangle) and inhibition of SST interneurons (yellow oval). **(B)** Representative patch-clamp current-clamp recordings from a ChR2-negative principal neuron *in vivo* to blue light stimulation alone (Pre and Post) and to combined blue and yellow light stimulation (SST-inhibition) during an activated state. **(C-E)** Negative peak amplitude (C), area (D), and response probability (E) of light-evoked membrane potential responses under the conditions shown in (B). n = 12 cells from 6 animals. One-way repeated measures ANOVA. **(F)** Schematic of the experimental configuration in brain slices. **(G)** Representative trace from an Arch-expressing SST interneuron showing efficient cell hyperpolarization following yellow light illumination (yellow bar). Blue light pulse (blue bar) delivered during photoinhibition resulted in subthreshold EPSP. **(H)** Schematic of the recordings in brain slices from either ChR2-negative (grey triangle) or ChR2-positive (blue triangle) neurons. ChR2-negative cells were located both in layer II/III and V. **(I)** IPSCs evoked in recorded cells by layer V stimulation before (Pre), during (SST-inhibition), and after (Post) photoinhibition of SST interneurons. **(J-K)** Amplitude (J) and charge transfer (K) of IPSCs under the different experimental conditions. n = 35 cells. One-way repeated measures ANOVA.

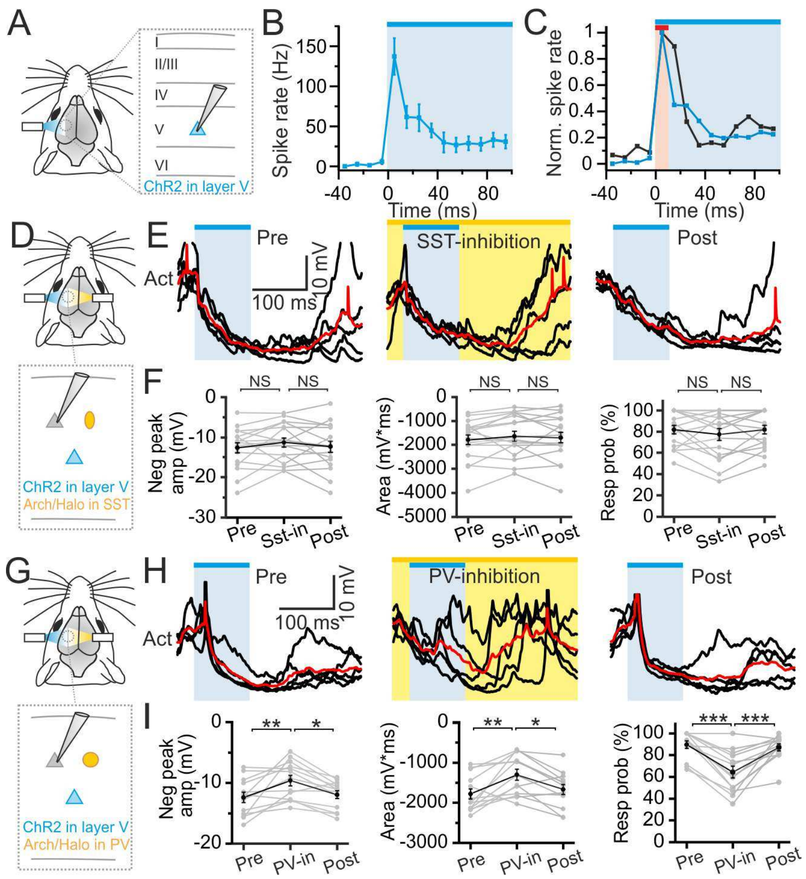

Figure S7. Effects of photoinhibition of SST or PV interneurons on cortical responses to prolonged layer V stimulation. Related to Figure 7. (A) Schematic of the experimental configuration. Juxtasomal recordings of layer V Thy1-ChR2-positive cells were performed in anesthetized animals during blue light stimulation (stimulus duration: 100 ms). **(B)** Average peri-stimulus time histogram (bin: 10 ms) of layer V ChR2-positive neurons during blue light stimulation (stimulus duration: 100 ms). n = 10 cells from 8 animals. **(C)** Normalized average peri-stimulus time histogram for cells reported in panel B (blue line) and in Figure 1E (black line) showing similar temporal profile of layer V spike activity during layer V optogenetic stimulation (blue bar) and during whisker stimulation (red bar). **(D)** *In vivo* recordings of ChR2-negative principal neurons (grey triangle) were performed in both superficial and deep layers during concurrent photoactivation of a subpopulation of layer V principal neurons (blue triangle) and photoinhibition of SST interneurons (yellow oval). **(E)** Five representative current-clamp patch-clamp traces (black) and their mean (red) from a ChR2-negative principal neuron *in vivo* during optogenetic stimulation of layer V with a prolonged blue light stimulus (Pre and Post, stimulus duration: 100 ms) and during combined blue and yellow light stimulation (SST-inhibition) during an activated state. **(F)** Negative peak amplitude (left), area (middle), and response probability (right) of light-evoked membrane potential responses under the three experimental conditions shown in (E). n = 17 cells from 8 animals. One-way repeated measures ANOVA. **(G)** Same as in (D) for photoinhibition of PV interneurons (yellow oval). **(H)** Same as in (E) for PV-inhibition. **(I)** Same as in (F) for PV-inhibition. n = 13 cells from 7 animals. One-way repeated measures ANOVA.

Laser power (mW)	Subthreshold latency (ms)	# of APs *per* stimulus	Time to 1st spike (ms) [# cells displaying APs]	Time to 2nd spike (ms) [# cells displaying APs]	Time to 3rd spike (ms) [# cells displaying APs]	# of recorded cells
18	0.19 ± 0.03	1.33 ± 0.19	3.3 ± 0.5 [n = 11]	11.1 ± 0.8 [n = 5]	N/A	12
13	0.20 ± 0.03	1.24 ± 0.18	3.5 ± 0.6 [n = 10]	12.7 ± 1.2 [n = 5]	N/A	11
7	0.22 ± 0.04	1.21 ± 0.17	4.4 ± 0.9 [n = 11]	11.9 ± 1.4 [n = 3]	24.0 ± 0.0 [n = 1]	11
4.6	0.26 ± 0.03	1.17 ± 0.17	4.6 ± 0.7 [n = 11]	11.6 ± 1.0 [n = 3]	N/A	12
1.8	0.51 ± 0.10	0.97 ± 0.25	6.4 ± 1.0 [n = 8]	12.8 ± 2.7 [n = 2]	20.9 ± 0.0 [n = 1]	11
0.14	1.14 ± 0.17	0.42 ± 0.20	8.4 ± 1.3 [n = 4]	12.7 ± 0.00 [n = 1]	N/A	11

Table S1. Light-evoked responses in ChR2-positive neurons in brain slices. Related to Figure 1 and Figure S1. Layer V ChR2-positive cells were recorded in patch-clamp current-clamp mode in isolated cortical slices. Blue light of different intensities (laser power) was shined on the sample for 10 ms through a fiber optic. ChR2-positive cells were characterized by subthreshold latency <1 ms at laser power > 1 mW (at the fiber exit, see STAR Methods).

	Subthreshold latency in the Res. state (ms)	Subthreshold latency in the Act. state (ms)	Significance
Exc. LII/III (n = 12)	2.32 ± 0.17	2.44 ± 0.13	* *vs* LII/III awake in Res. state<br>* vs LV in Act. state<br>* vs LVI in Act. state<br>* *vs* LII/III wide-field DMD in Act. state<br>*** *vs* LII/III awake in Act. state
Exc. LIV (n = 4)	2.79 ± 0.38	2.81 ± 0.26	* *vs* LII/III awake in Res. state<br>** vs LV in Act. state<br>* *vs* LII/III awake in Act. state
Exc. LV (n = 13)	2.14 ± 0.16	2.03 ± 0.12	* *vs* LII/III in Act. state<br>** *vs* LIV in Act. state<br>* *vs* LII/III wide-field DMD in Act. state
Exc. LVI (n = 12)	2.17 ± 0.23	2.01 ± 0.22	* *vs* LII/III in Act. state<br>* *vs* LII/III patterned DMD in Act. state<br>* *vs* LII/III wide-field DMD in Act. state
Exc. LII/III (n = 6) Patterned DMD	2.73 ± 0.30	3.02 ± 0.47	* *vs* LII/III awake in Res. state<br>* *vs* LVI in Act. state<br>* *vs* LII/III awake in Act. state
Exc. LII/III (n = 6) Wide-field DMD	2.67 ± 0.34	3.19 ± 0.42	* *vs* LII/III awake in Res. state<br>* *vs* LII/III in Act. state<br>* *vs* LV in Act. state<br>* *vs* LVI in Act. state<br>* *vs* LII/III awake in Act. state
Exc LII/III (n = 7) Awake	1.84 ± 0.15	1.75 ± 0.06	*** *vs* LII/III in Act. state<br>* *vs* LIV in Act. state<br>* *vs* LII/III DMD in Act. state<br>* *vs* LII/III wide-field DMD in Act. state

Table S2. Light-evoked responses of ChR2-negative cells *in vivo*. Related to Figure 1 and Figures S2 and S3. Principal ChR2-negative cells were recorded in current-clamp patch-clamp mode *in vivo*. Student's *t*-test was used for statistical comparison between two given classes of cells. When unequal variances were detected, the Welch's correction was used. Mann-Whitney test was applied in the case of non-parametric data.

	Laser power: 18-14 mW Vm = -70 mV	Laser power: 4.6-5 mW Vm = -70 mV	Laser power: 18-14 mW Vm = -50 mV	Laser power: 4.6-5 mW Vm = -50 mV
**Exc. LV (n=12)**				
Subthreshold latency (ms)	2.29 ± 0.15	3.05 ± 0.56	2.57 ± 0.21	3.18 ± 0.53
# of cells with APs	0	0	0	1
# of APs *per* stimulus	0	0	0	0.38 ± 0.00 (n = 1)
**Exc. LII/III (n=10)**				
Subthreshold latency (ms)	3.77 ± 0.18	4.79 ± 0.38	3.86 ± 0.24	5.62 ± 0.78
# of cells with APs	6	0	8	2
# of APs *per* stimulus	0.85 ± 0.15 (n = 6)	0	0.90 ± 0.10 (n = 8)	1 ± 0 (n = 2)
**Exc. LIV (n=10)**				
Subthreshold latency (ms)	2.44 ± 0.12	2.54 ± 0.14	2.35 ± 0.18	2.78 ± 0.19
# of cells with APs	0	0	2	0
# of APs *per* stimulus	0	0	0.27 ± 0.13 (n = 2)	0

Table S3. Light-evoked responses in principal ChR2-negative neurons in brain slices. Related to Figure 5. Layer V, II/III, and IV principal ChR2-negative cells were recorded in patch-clamp current-clamp mode in isolated cortical slices. Blue light of different intensities was shined for 10 ms through a fiber optic. Laser power used in Figure 5 was 4.6-5 mW.

	Subthreshold latency (ms)	Significance
FS LV (n = 9)	2.09 ± 0.21	*** *vs* NFS LII/III
NFS LV (n = 11)	2.66 ± 0.21	*** *vs* NFS LII/III<br>* *vs* FS LIV
FS LII/III (n = 6)	2.73 ± 0.25	** *vs* NFS LII/III<br>* *vs* FS LIV
NFS LII/III (n = 14)	4.62 ± 0.37	*** *vs* FS LV<br>*** *vs* NFS LV<br>** *vs* FS LII/III<br>*** *vs* FS LIV
FS LIV (n = 8)	1.94 ± 0.16	* *vs* NFS LV<br>* *vs* FS LII/III<br>*** *vs* NFS LII/III

Table S4. Subthreshold latencies of light-evoked responses in interneurons recorded in brain slices. Related to Figure 6. Interneurons were recorded in patch-clamp current-clamp mode in isolated cortical slices. Blue light (power: 4.6-5 mW) was shined for 10 ms through a fiber optic. Student's *t*-test was used for statistical comparison among two given classes of cells. When unequal variances were detected, the Welch's correction was used. Mann-Whitney test was applied in the case of non-parametric data.

	Exc. LV (n = 12)	Exc. LII/III (n = 10)	Exc. LIV (n = 10)	FS LV (n = 9)	NFS LV (n = 11)	FS LII/III (n = 6)	NFS LII/III (n = 14)	FS LIV (n = 8)
V rest (mV)	-69 ± 2	-78 ± 2	-72 ± 2	-70 ± 2	-65 ± 2	-67 ± 3	-66 ± 3	-68 ± 1
Input Resistance (MΩ)	154 ± 15	125 ± 14	129 ± 19	110 ± 16	175 ± 24	71 ± 8	212 ± 22	65 ± 8
Rheobase (pA)	121 ± 14	140 ± 19	145 ± 34	300 ± 42	168 ± 38	442 ± 40	118 ± 15	419 ± 38
Firing frequency max (Hz)	40 ± 3	35 ± 3	40 ± 2	197 ± 19	66 ± 10	148 ± 15	53 ± 7	189 ± 13
First spike threshold (mV)	-39 ± 1	-37 ± 1	-41 ± 1	-42 ± 2	-42 ± 2	-37 ± 2	-40 ± 7	-39 ± 2
First spike amplitude (mV)	75 ± 4	71 ± 3	80 ± 2	69 ± 4	68 ± 5	51 ± 6	74 ± 3	61 ± 3
First spike duration (ms)	1.36 ± 0.14	1.79 ± 0.08	1.14 ± 0.04	0.43 ± 0.05	0.82 ± 0.06	0.55 ± 0.05	0.99 ± 0.10	0.40 ± 0.02
First spike AHP (mV)	14 ± 1	14 ± 1	13 ± 2	22 ± 1	13 ± 2	18 ± 3	12 ± 1	19 ± 1
ISI_1/ISI_n	0.34 ± 0.06	0.32 ± 0.03	0.45 ± 0.03	0.66 ± 0.03	0.41 ± 0.06	0.67 ± 0.06	0.31 ± 0.04	0.59 ± 0.04

Table S5. Biophysical properties of recorded neurons in brain slice preparation. Related to Figures 5 and 6. AHP, after hyperpolarization; ISI_1, inter spike interval between the first pair of discharged spikes; ISI_n, inter spike interval between the last pair of discharged spikes.
