# Supplementary material for: Temporal Sharpening of Sensory Responses by Layer V in the Mouse Primary Somatosensory Cortex
Source: Curr Biol. 2020 May 4;30(9):1589–1599.e10. doi: 10.1016/j.cub.2020.02.004 (PMC7198976; doi:10.1016/j.cub.2020.02.004)
Supplement: Document S2. Article plus Supplemental Information [file mmc2.pdf]

Article

Current Biology

Temporal Sharpening of Sensory Responses by Layer V in the Mouse Primary Somatosensory Cortex

Graphical Abstract

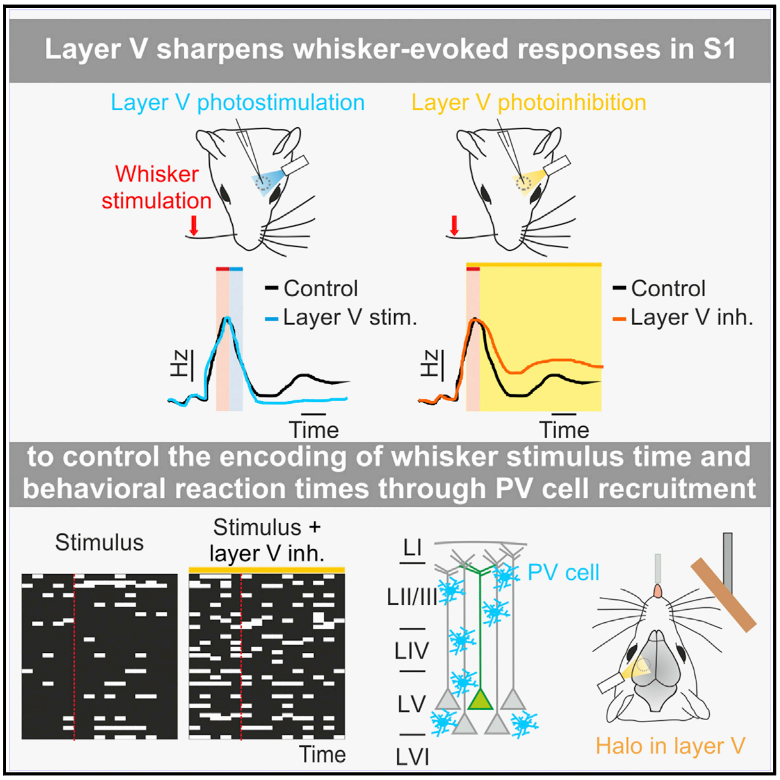

Highlights

- Layer V pyramidal neurons sharpen the temporal profile of sensory responses in S1
- Layer V pyramidal cells control the precision of encoding of whisker stimulus time
- Inhibition of layer V increases behavioral reaction time in a discrimination task
- The effect of layer V activation is largely mediated by PV-positive interneurons

Authors

Dania Vecchia, Riccardo Beltramo, Fabio Vallone, ..., Anthony Holtmaat, Stefano Panzeri, Tommaso Fellin

Correspondence

tommaso.fellin@iit.it

In Brief

Using cell-type-specific optogenetic manipulations, Vecchia et al. show that layer V pyramidal cells control the precision of encoding of whisker stimulus time in the primary somatosensory cortex through the activation of a specific inhibitory circuit. Layer V activity impacts behavioral reaction time in a whisker-based texture discrimination task.

Vecchia et al., 2020, Current Biology *30*, 1589–1599
May 4, 2020 © 2020 The Author(s). Published by Elsevier Ltd.
https://doi.org/10.1016/j.cub.2020.02.004

CellPress

Current Biology
Article

CellPress

Temporal Sharpening of Sensory Responses by Layer V in the Mouse Primary Somatosensory Cortex

Dania Vecchia,[1,2] Riccardo Beltramo,[1] Fabio Vallone,[2] Ronan Chéreau,[3] Angelo Forli,[1,2] Manuel Molano-Mazón,[2,4] Tanika Bawa,[3] Noemi Binini,[1,2] Claudio Moretti,[1,2] Anthony Holtmaat,[3] Stefano Panzeri,[2,4] and Tommaso Fellin[1,2,5,*]
[1]Optical Approaches to Brain Function Laboratory, Istituto Italiano di Tecnologia, 16163 Genova, Italy
[2]Neural Coding Laboratory, Istituto Italiano di Tecnologia, 16163 Genova and 38068 Rovereto, Italy
[3]Department of Basic Neurosciences, Geneva University Neurocenter, Faculty of Medicine, University of Geneva, 1206 Geneva, Switzerland
[4]Neural Computation Laboratory, Center for Neuroscience and Cognitive Systems @UniTn, Istituto Italiano di Tecnologia, 38068 Rovereto, Italy
[5]Lead Contact
*Correspondence: tommaso.fellin@iit.it
https://doi.org/10.1016/j.cub.2020.02.004

SUMMARY

The timing of stimulus-evoked spikes encodes information about sensory stimuli. Here we studied the neural circuits controlling this process in the mouse primary somatosensory cortex. We found that brief optogenetic activation of layer V pyramidal cells just after whisker deflection modulated the membrane potential of neurons and interrupted their long-latency whisker responses, increasing their accuracy in encoding whisker deflection time. In contrast, optogenetic inhibition of layer V during either passive whisker deflection or active whisking decreased accuracy in encoding stimulus or touch time, respectively. Suppression of layer V pyramidal cells increased reaction times in a texture discrimination task. Moreover, two-color optogenetic experiments revealed that cortical inhibition was efficiently recruited by layer V stimulation and that it mainly involved activation of parvalbumin-positive rather than somatostatin-positive interneurons. Layer V thus performs behaviorally relevant temporal sharpening of sensory responses through circuit-specific recruitment of cortical inhibition.

INTRODUCTION

The cortex is crucially involved in sensory perception, and its function is dictated by its architecture [1]. Sensory cortices are organized in six horizontal layers containing functionally distinct neuronal subnetworks [2–12]. Elucidating the logic of interactions within and between cortical layers is essential for understanding the cellular basis of perception. Among sensory cortices, the whisker system is a preferred model for studying the mechanisms of sensory processing [13, 14]. Through their whiskers, rodents detect and localize objects [15–18] and discriminate their textures [19–21]. Contacts with objects trigger patterns of whisker deflections whose temporal structure is crucial for sensory perception [21–26] and that is in part encoded as precisely timed patterns of cortical spikes [20, 24, 26–29]. However, the neural circuits controlling how neural responses encode the timing of sensory stimuli remain largely unresolved.

In this paper, we demonstrate that activity of layer V pyramidal cells sharpens the temporal neuronal responses to whisker stimuli, leading to more precise encoding of whisker deflection or touch time in the primary somatosensory cortex (S1). Inhibiting layer V activity increased the animal's behavioral reaction time in a whisker-based texture discrimination task. Moreover, our experiments suggest that layer V modulation of sensory responses occurs through intracortical axons recruiting a specific inhibitory circuit. Besides being a main cortical output, layer V neurons perform behaviorally relevant tuning of sensory responses within the cortex.

RESULTS

Activation of Layer V Triggers Membrane Potential-Dependent Responses in Neurons across Cortical Layers *In Vivo*

To specifically manipulate layer V pyramidal neurons, we initially used *Rbp4-cre* transgenic mice in combination with the injection of an adeno-associated virus (AAV) carrying a double-floxed channelrhodopsin-2 (ChR2) construct (Figure S1; Table S1). We first recorded from ChR2-negative principal cells (STAR Methods) in S1 of urethane-anesthetized mice during spontaneous activity (Figure S2; Table S2). When the membrane potential was close to resting, we found that all neurons (n = 55) responded to layer V photoactivation with a pronounced depolarization (Figures S2A and S2A$_1$, top panel; "positive peak," Figure S2B), followed by a small, long-lasting hyperpolarization ("negative peak," Figure S2B$_1$). In the activated state (a spontaneously occurring depolarized state [30–33]), the majority of recorded cells (38/55) responded to blue light with a small depolarization followed by a large, long-lasting hyperpolarization (Figures S2A and S2A$_1$, bottom panel; and S2B–S2B$_2$). The small depolarizing component led to firing in a minority of cells (8/55). In 17/55 neurons only the hyperpolarization was observed. These membrane potential dynamics occurred across layers,

This is an open access article under the CC BY license (http://creativecommons.org/licenses/by/4.0/).

as verified by identifying cell location through post hoc biocytin staining (Figures S2C–S2J$_2$). Responses to layer V activation were similar in awake animals when we used an illumination system based on a digital micromirror device (DMD; Figures S3A–S3G$_2$), which provided complex spatial illumination patterns to ChR2-expressing neurons (Figures S3H–S3K$_2$).

Temporal Sharpening of Cortical Responses to Sensory Inputs by Layer V

To investigate whether layer V activation modulates sensory inputs, we combined single-whisker stimulation with optogenetic manipulation of layer V. We first recorded in whole-cell configuration the membrane potential of ChR2-negative cells in the barrel column corresponding to the stimulated whisker in anesthetized mice during resting states (Figures 1A–1B$_1$). A stepwise whisker deflection (duration, 10 ms) caused a depolarizing response, frequently driving the recorded cells into an activated state [30]. When layer V was photostimulated (duration, 10 ms) during a whisker-evoked activated state, all neurons responded with a small, transient depolarization followed by a pronounced hyperpolarization (Figures 1B–1C$_1$). Responses during combined whisker and optogenetic stimulation were similar across cortical layers II/III, V, and VI (Figures S3L–S3Q$_2$).

To evaluate the effect of layer V activation on the sensory-evoked firing, we performed juxtasomal recordings from 27 opsin-negative (STAR Methods) cortical cells. Because layer V cells discharge action potentials (APs) in response to sensory inputs with various latencies [34, 35], we delivered blue light at different delays ($\sim$0, 30, and 50 ms) from the onset of the whisker-evoked response. In the 13 (out of 27) neurons responding with increased firing to the whisker deflection, blue light illumination decreased the whisker-evoked response (Figures 1D–1F) and the average time of individual spikes when the delay between onset of whisker-evoked response and light delivery was $\leq$30 ms (n = 8 cells from 6 animals, one-way repeated-measures ANOVA, p = 5E-4).

To demonstrate that endogenous spiking of layer V neurons similarly controls whisker-evoked responses, we expressed the inhibitory opsin halorhodopsin 3.0 (Halo) in a subpopulation of layer V neurons (Figures 1G, S4A, and S4A$_1$). We then performed juxtasomal electrophysiological recordings from 70 opsin-negative cortical neurons, 19 of which were classified as responsive to whisker deflection. When whisker stimulation was paired with photoinhibition (duration, 500 ms) of layer V neurons, the spike rate of the late (40–100 ms from the onset of the whisker deflection) neuronal response was increased, whereas no effect was observed on the spike rate of the early (0–40 ms) response (Figures 1H–1J). The average time of individual whisker-elicited spikes was increased by layer V suppression (n = 19 neurons from 14 mice, paired t test, p = 0.027).

Modulation of the Accuracy of Stimulus Time Encoding by Layer V

The temporal sharpening or widening of whisker-evoked response observed with activation and inactivation of layer V pyramidal cells led us to hypothesize that layer V modulates the accuracy of encoding of whisker deflection time in barrel cortical activity. The occurrence and timing of whisker deflections are signaled when several cortical neurons fire within a short window [36, 37]. To test our hypothesis, we thus built single-trial pseudo-simultaneous population responses of whisker-responsive neurons (Figures 2A–2D). We defined a population response event (PRE) as the firing of at least a certain fraction of neurons within a 10-ms bin (Figures 2A$_1$–2D$_1$; STAR Methods). We quantified the effect of optogenetic activation (Figures 2B and 2B$_1$) and inactivation (Figures 2D and 2D$_1$) of layer V on how the stimulus time was encoded by PREs, compared to control conditions without illumination (Figures 2A, 2A$_1$, 2C, and 2C$_1$). Layer V stimulation occurring at latencies $\leq$30 ms from the whisker response onset elicited a distribution of PRE times shorter than in the absence of blue light (Figures 2E and 2E$_1$). In contrast, layer V inactivation led to longer PRE times (Figures 2F and 2F$_1$). We compared the absolute error of stimulus time estimation from PRE times (estimated by subtracting from each PRE time the mode of its distribution) across different conditions. We found that the absolute error was reduced by layer V activation with latencies $\leq$0 ms from the whisker response onset (Figures 2G and S4C–S4F) and increased by layer V inactivation (Figures 2H and S4G–S4J). Simultaneous recording of neuronal spiking across cortical layers using linear probes confirmed these results (Figures S4K–S4R).

Layer V Controls the Accuracy of Touch Time Encoding in Actively Sensing Animals

We performed juxtasomal recordings from S1 neurons during the presentation of a vertical pole in the contralateral whisker field (Figure 3A). Mice were free to run on a wheel and to contact the pole through whisking, all but one whisker were trimmed, and recordings were targeted to the principal barrel column of the spared whisker. We restricted our analysis to touch-responsive deep cells (STAR Methods). Layer V neurons increased their firing rate after pole touch (Figures 3B and 3C). Optogenetic inhibition of layer V increased the firing rate of cortical neurons both in the pre-touch and in the post-touch time window (Figures 3B$_1$ and 3C$_1$; pre-touch spike rate: 5 ± 1 Hz versus 12 ± 3 Hz under control conditions and during layer V photoinhibition, paired t test, p = 8E-3; post-touch spike rate: 7 ± 1 Hz versus 12 ± 2 Hz under control conditions and during layer V photoinhibition, paired t test, p = 0.015). Moreover, layer V inhibition eliminated the touch-induced increase in neuronal firing rate (Figure 3D). As a consequence, the absolute error of stimulus time estimation increased (Figures 3E, 3E$_1$, and 3F).

Layer V Inactivation Increases Reaction Times in a Texture Discrimination Task

To determine whether the decreased encoding accuracy of touch time triggered by layer V inhibition impacts perceptual behavior, we performed experiments in head-fixed *Rbp4-cre* mice expressing Halo and performing a go/no-go texture discrimination task (Figures 4A and 4A$_1$). The mouse's performance remained at around 80% correct upon optogenetic illumination on the cranial window (Light WIN) or when light was delivered in a region outside the cranial window (Light EXT, Figure 4B). However, reaction times (RTs) in Hit trials were longer during optogenetic inactivation of layer V cells compared to RTs when the light was not presented (mean RT: 989 ± 80 ms versus 921 ± 78 ms in Light WIN and Light OFF,

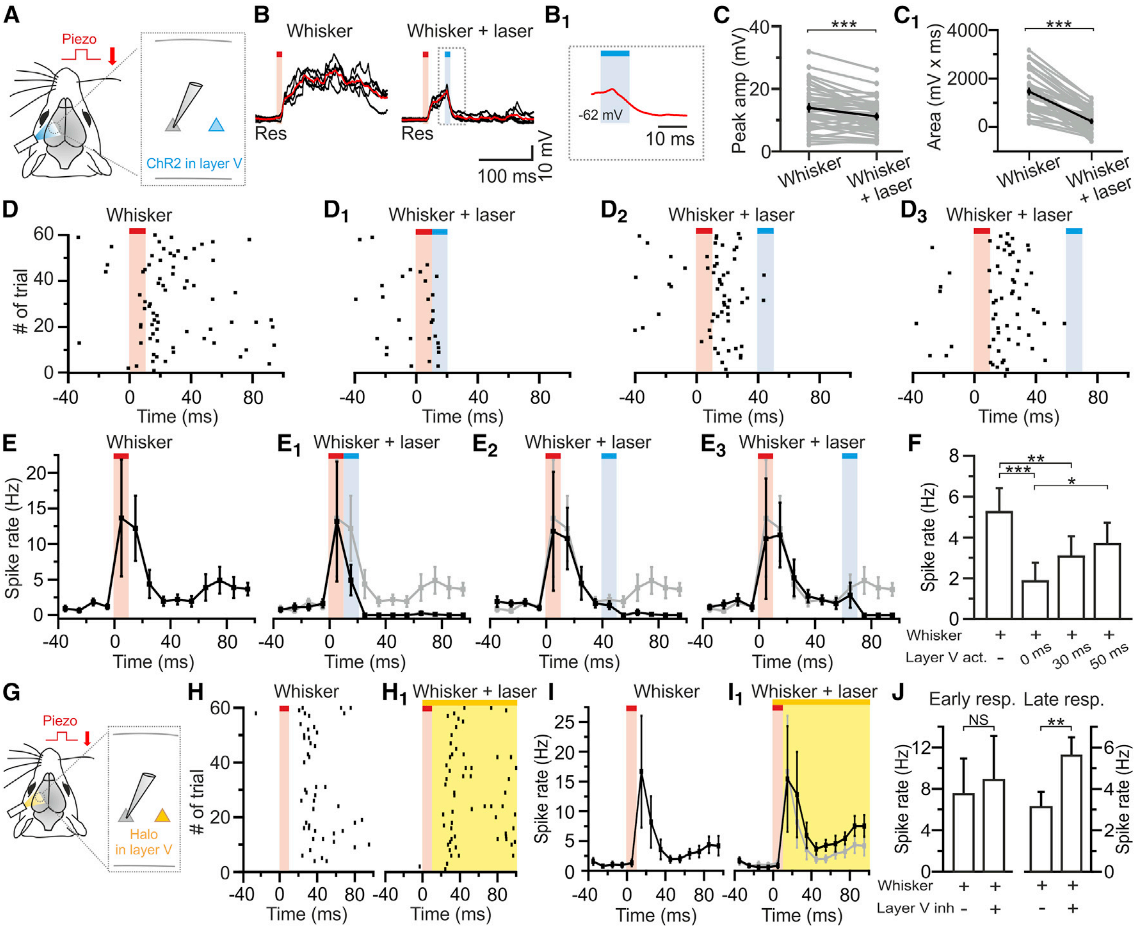

Figure 1. Layer V Sharpens the Temporal Response to Whisker Stimulation

(A) Schematic of the experiment in anesthetized animals. In this as well as in other figures, ChR2-positive neurons are indicated in blue, ChR2-negative cells in grey.

(B) Representative traces from a layer II/III ChR2-negative principal neuron *in vivo*.

(B_1) Average response in (B) at a finer temporal scale.

(C and C_1) Peak amplitude (C) and area (C_1) in ChR2-negative layers II/III, V, and VI cells under the different experimental conditions. n = 39 cells from 17 animals; paired t test. In this as well as in other figures, values from individual experiments are shown in grey, the average of all cells in black. Error bars indicate SEM.

(D–D_3) Spiking response of a deep neuron under the different experimental conditions. Blue light was presented at different delays (~0 ms in D_1, ~30 ms in D_2, ~50 ms in D_3) from the onset of the whisker-evoked response.

(E–E_3) Peri-stimulus time histogram (black trace, bin 10 ms) of whisker-responsive deep cortical neurons during whisker stimulation (red bar, E) or during whisker stimulation followed by layer V activation (blue bar, E_1–E_3). Gray traces in (E_1)–(E_3) show the average peri-stimulus histogram shown in (E).

(F) Cellular spike rate in a time window of 100 ms from the onset of the whisker stimulation under the different experimental conditions shown in (E)–(E_3). n = 13 cells from 7 animals; Friedman test.

(G) Schematic of the experimental configuration.

(H and H_1) Raster plots showing the response of a representative deep cortical neuron to whisker stimulation (red bar) alone (H) and during concurrent layer V photosuppression (yellow bar, H_1).

(I and I_1) Peri-stimulus time histograms (black trace) of whisker-responsive deep cortical neurons recorded during whisker stimulation (red bar, I) and during whisker stimulation and concurrent layer V photoinhibition (yellow bar, I_1). The gray trace in (I_1) represents the average peri-stimulus histogram shown in (I).

(J) Spike rate in the early (0–40 ms) and late (40–100 ms) response window under the different experimental conditions shown in (I) and (I_1). n = 19 cells from 14 animals; early response, Wilcoxon test; late response, paired t test. In this as well as in other figures, *$p < 0.05$; **$p < 0.01$; ***$p < 0.001$. Error bars indicate SEM. See also Figures S1–S4 and Tables S1 and S2.

respectively; one-tailed paired t test, p = 0.014 Holm-Bonferroni corrected, n = 15 sessions from 5 animals; Figure 4C, left panel) and when the light was delivered outside the cranial window (mean RT: 989 ± 80 ms versus 945 ± 80 ms under Light WIN and Light EXT, respectively; one-tailed paired t test, p = 0.04 Holm-Bonferroni corrected; Figure 4C, right panel). We verified that light *per se* did not affect the animal's performance (one-tailed paired t test, p = 0.22 between Light OFF and Light EXT, n = 15 sessions from 5 animals) and RTs (mean RT: 921 ± 78 ms versus 945 ± 80 ms in Light OFF and Light EXT, respectively; one-tailed paired t test with Holm-Bonferroni correction, p = 0.20; n = 15 sessions from 5 animals). Importantly, local application of the GABA agonist muscimol in the barrel field of S1 reduced the performance to chance levels (Figure 4D).

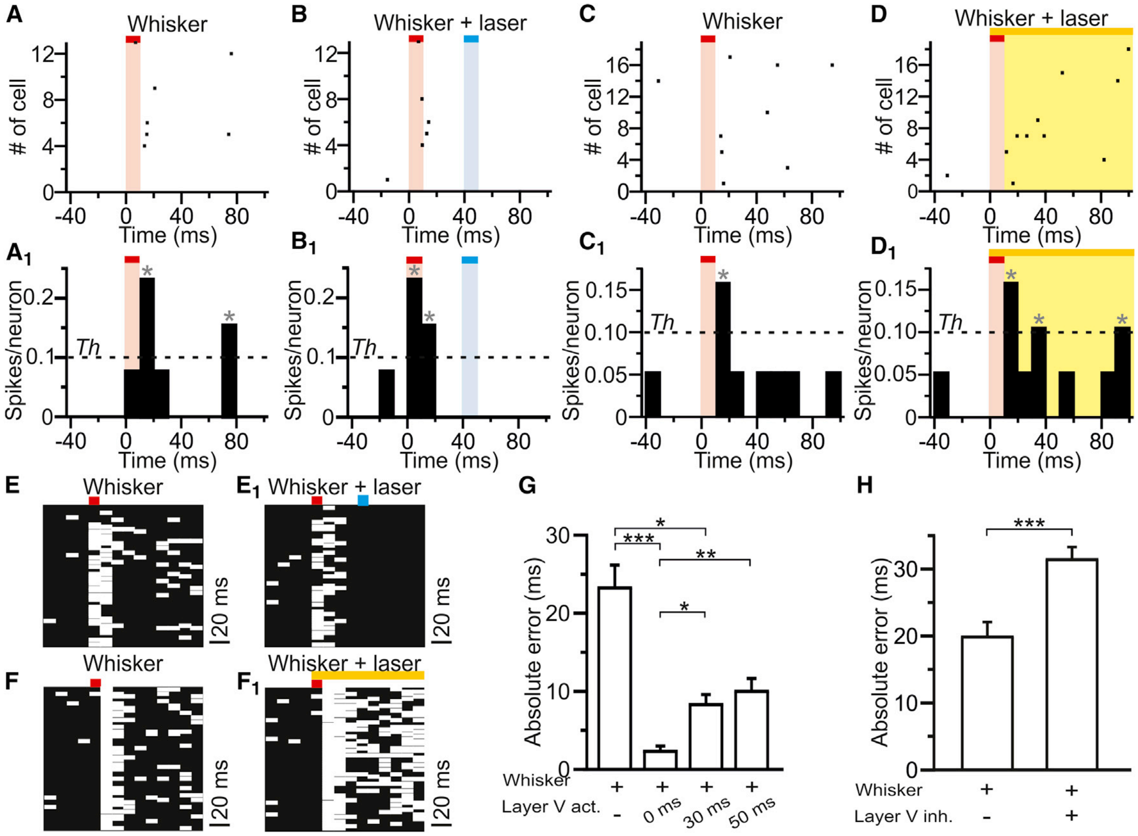

Figure 2. Layer V Increases the Precision of Encoding of Whisker Deflection Time

(A and B) Representative pseudo-simultaneous population response to whisker stimulation (red bar, A) for the neurons displayed in Figures 1E and 1F, in the absence (A) or presence of layer V photoactivation (blue bar, B).

(A_1 and B_1) Number of spikes per neuron for the same single trials shown in (A) and (B). The dashed line indicates the threshold (*Th*) for population response event (PRE) detection (see STAR Methods). Gray asterisks indicate PREs.

(C and D) Same as (A) and (B), respectively, for whisker-responsive neurons displayed in Figures 1H–1J.

(C_1 and D_1) Same as (A_1) and (B_1) for the same trials shown in (C) and (D).

(E and E_1) PREs in all pseudo-simultaneous whisker stimulation trials for the same neurons shown in (A) and (B) in the absence (E) or presence of layer V photoactivation (blue bar, E_1).

(F and F_1) Same as in (E) and (E_1) for layer V photosuppression.

(G) Absolute estimate time error of PREs (absolute error) following whisker deflection and whisker deflection in the presence of layer V photostimulation. n = 99 (no layer V activation), n = 39 (layer V activation at ~0 ms), n = 59 (layer V activation at ~30 ms), and n = 65 (layer V activation at ~50 ms) PREs from n = 13 neurons from 7 animals; Kruskal-Wallis test.

(H) Same as in (G) for layer V photoinhibition. n = 138 (no layer V inactivation) and n = 237 (layer V inactivation) PREs from n = 19 neurons from 14 animals; Mann-Whitney test.

See also Figure S4.

Intracortical Layer V Projections Are Sufficient to Spread Excitation and Recruit Inhibitory Networks across Layers

The effects of layer V activation on cortical cells *in vivo* could be mediated by direct, intracortical layer V projections or by an indirect loop in which layer V neurons project to a subcortical structure, which then projects back to the same cortical area. To discriminate between these possibilities, we performed combined patch-clamp recordings and optogenetic manipulations in brain slices in which the cortex was isolated from the rest of the brain (Figure 5; Table S3). We recorded from 32 ChR2-negative principal neurons, 12 from layer V (Figures 5A–5B_2), 10 from layer II/III (Figures 5C–5D_2), and 10 from layer IV (Figures 5E–5F_2). All recorded cells responded to blue light illumination with a pronounced depolarization when the cell was close to its resting potential, which turned into a transient depolarization followed by a large hyperpolarization when the cell was held at −50 mV. Few neurons (3/32) responded with an AP discharge during the initial transient depolarizing phase at depolarized holding potentials. All responses were reduced by DNQX (10 μM) and D-AP5 (50 μM, average peak response: control, 6.3 ± 1.8 mV; DNQX/D-AP5, 0.14 ± 0.14 mV, n = 4, paired t test, p = 0.036).

The hyperpolarizing response observed in brain slice experiments at depolarized membrane potential is consistent with the recruitment of inhibitory networks by layer V activation. We tested this hypothesis recording from electrophysiologically identified layer V, layer II/III, and layer IV interneurons in slices (Figure 6; Table S4). Interneurons were divided into two groups (Figures

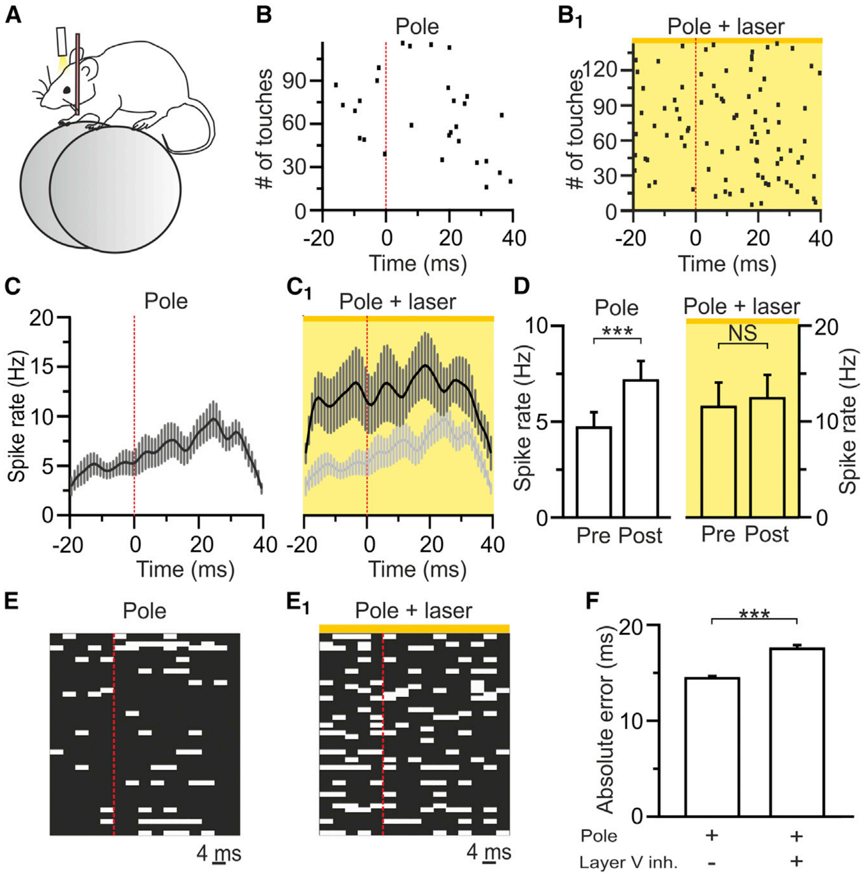

Figure 3. Layer V Controls the Encoding of Touch Time during Active Whisking

(A) Juxtasomal recordings were performed in awake animals.

(B and B_1) Representative cell response during pole presentation trials (Pole, B) and touches during concurrent pole presentation and yellow light stimulation (Pole + laser, B_1). Spikes were aligned to touch onset (0 ms, red line).

(C) Average peri-touch time histograms of touch-responsive deep neurons under the different experimental conditions. n = 12 cells from 9 mice.

(D) Spike rate in the pre (−20–0 ms) and in the post (0–40 ms) touch window during pole presentation (Pole) and during simultaneous pole presentation and yellow light (Pole + laser). n = 12 cells from 9 animals; paired t test.

(E and E_1) Representative PRE distribution for the same neurons shown in (C) and (C_1), under the different experimental conditions.

(F) Absolute error of touch time during pole presentation and during pole presentation in the presence of layer V photoinhibition. n = 12 cells from 9 animals; binomial test.

See also Figure S4.

6A and $6A_1$; Table S5): fast spiking (FS) and non-fast spiking (NFS) [38, 39]. Across layers, all FS cells and NFS cells responded to layer V photostimulation with a depolarization (Figures 6B–$6C_2$), which was blocked by DNQX and D-AP5 application (average response peak: control, 13.3 ± 3.9 mV; DNQX/D-AP5, 0.7 ± 0.4 mV; n = 5; paired t test, p = 0.024). Layer V photoactivation elicited firing in 7/9 FS and 4/11 NFS layer V cells, in 5/6 FS and 5/14 NFS layer II/III cells, and in 7/8 FS layer IV interneurons (Figure $6C_3$). To investigate whether layer V recruited inhibition through direct layer V-interneuron connection, we recorded light-evoked excitatory postsynaptic currents (EPSCs) from layer II/III inhibitory (and excitatory) neurons in the presence of tetrodotoxin (TTX) (0.5–1 μM). In all the NFS (n = 3) and FS (n = 4) neurons and in 7 out of 9 pyramidal neurons, we recorded EPSCs of significant amplitude (Figures 6D and $6D_1$). Light-evoked EPSCs were recorded in TTX also in 4 layer IV FS interneurons (EPSC amplitude, −15.7 ± 0.8 pA, n = 4 cells). EPSCs were abolished by NBQX and D-AP5 (n = 9 cells).

Given that layer V photostimulation drives both FS and NFS interneurons to fire and interneurons innervate pyramidal neurons, layer V photostimulation should trigger inhibitory postsynaptic currents (IPSCs) in ChR2-negative cells. To test this hypothesis, we performed voltage-clamp recordings in brain slices from ChR2-negative pyramidal cells in layer V, whereas stimulating the ChR2-positive pyramidal neurons in layer V. All recorded neurons displayed IPSCs following layer V stimulation (Figures 6E and 6F).

PV-Positive Interneurons Modulate Cortical Response to Layer V Stimulation

Is the inhibitory component of cortical responses to layer V activation mainly dependent on one specific class of interneurons? It has been shown that a subpopulation of somatostatin (SST) interneurons provide strong di-synaptic inhibition to principal neurons within layer V [40, 41]. We thus first performed combinatorial optogenetic experiments in which we expressed inhibitory opsins in SST interneurons, whereas expressing ChR2 in a subpopulation of layer V cells. We crossed *Thy1-ChR2* line 18 mice, in which ChR2 is expressed mainly in a subpopulation of pyramidal layer V cells [42, 43], with *SST-cre* animals (Figure S5A) and performed injections of AAV carrying a flex inhibitory opsin construct together with AAV transducing TdTomato. Cre-mediated recombination occurred in GABAergic (Figure S5B) and SST-positive (Figure S5C) interneurons. Arch- or Halo-expressing cells displayed firing properties compatible with those of SST interneurons and yellow light (λ = 594 nm) suppressed AP firing in SST cells (Figures S5D and S5E). In the hemisphere contralateral to the AAV injection site in double-transgenic mice, blue light evoked state-dependent membrane potential responses in cortical cells, similar to those observed in *Rbp4-cre* mice. We then stimulated layer V pyramids with blue light while inhibiting SST interneurons with yellow light (Figure S6A) in the hemisphere ipsilateral to the injection site, *in vivo*. We found that the responses of supragranular and infragranular cells to blue light illumination were not affected by the photoinhibition of SST cells (n = 12 from 6 animals; Figures S6B–S6E). As important controls, we found that yellow light *in vivo* did not cause any change in the membrane potential of cells in the hemisphere contralateral to the injected one (n = 5 cells from 2 animals, one-way repeated-measures

CellPress

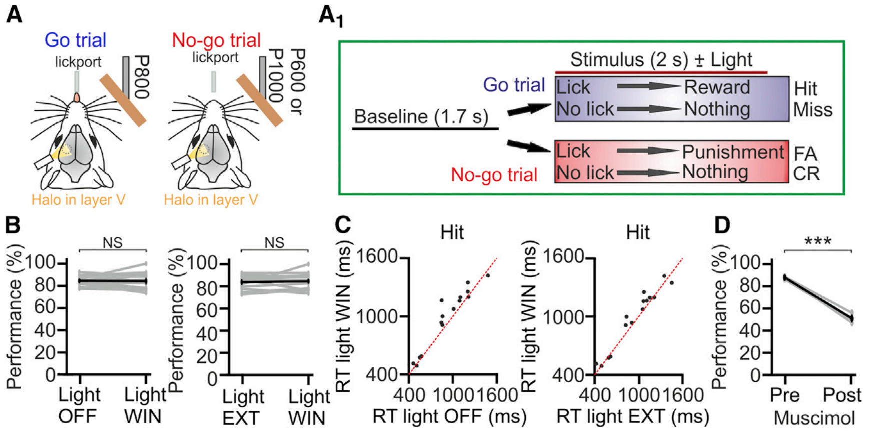

Figure 4. Inhibition of Layer V Increases Reaction Times in a Texture Discrimination Task

(A) Go/no-go texture discrimination task in head-fixed mice.

(A_1) Trial structure. CR, correct rejection; FA, false alarm.

(B) Left: performance during Light OFF and Light WIN. Right: performance during Light EXT and Light WIN. n = 15 sessions from 5 mice; one-tailed paired t test.

(C) Left: RTs in Hit trials during Light WIN as a function of the RTs in Hit trials during Light OFF. Right: RTs in Hit trials during Light WIN as a function of the RTs in Hit trials during Light EXT.

(D) Performance before (Pre) and after (Post) muscimol application in S1. n = 3 sessions from 3 mice; paired t test.

ANOVA, p = 0.52 and p = 0.13 for the resting and the activated states). We performed experiments using a prolonged blue light stimulus (stimulus duration, 100 ms), which generated a pattern of APs in ChR2-positive layer V neurons similar to the one elicited by whisker deflection (Figures S7A–S7C). Similar to what was observed before, we found that the responses to blue light were not affected by the SST photoinhibition (Figures S7D–S7F). Moreover, we recorded from opsin-positive SST interneurons in slice preparations and found that yellow light efficiently hyperpolarized recorded cells (Figures S6F and S6G; average hyperpolarization: −34 ± 6 mV, n = 9), whereas blue light delivered during yellow light illumination generated depolarization (average depolarization: 20 ± 5 mV, n = 9). The yellow light-induced hyperpolarization reduced spiking evoked by blue light (0.71 ± 0.19 spikes/stimulus versus 0 ± 0 spikes/stimulus, with blue light and with blue light in the presence of yellow light, respectively; n = 9, Wilcoxon test, p = 4E-3). We also recorded light-induced IPSCs in layer II/III and V cells during blue light alone or during concurrent illumination with blue and yellow light (Figures S6H–S6K). Photoinhibition of SST interneurons had a small impact on layer V-evoked IPSCs.

We next targeted inhibitory opsins to parvalbumin (PV) cells by crossing *Thy1-ChR2* mice with *PV-cre* mice (Figure S5F) and injecting the double-transgenic offspring with AAVs transducing

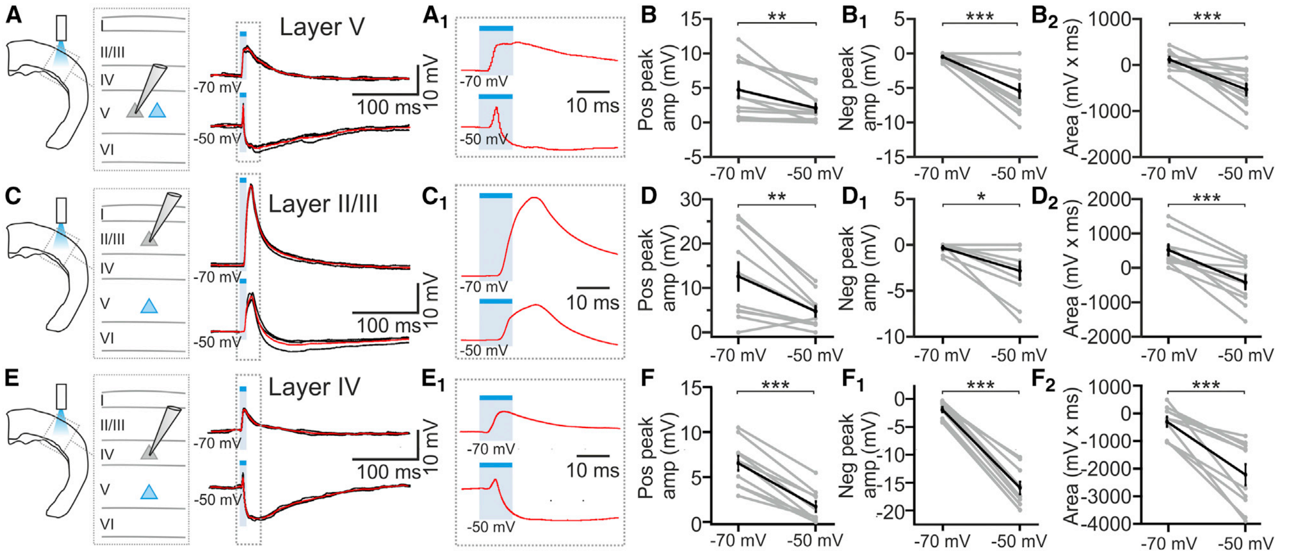

Figure 5. Intracortical Projections Are Sufficient to Mediate Membrane Potential Changes in Cortical Neurons

(A) Schematic (left) of the experimental configuration for slice recordings and representative traces from a ChR2-negative layer V pyramidal neuron (right) during stimulation of layer V.

(A_1) Zoom-in of the response shown in (A).

(B–B_2) Positive peak amplitude (B), negative peak amplitude (B_1), and area (B_2) of the light-evoked response in ChR2-negative layer V principal neurons. n = 12; paired t test.

(C–D_2) Same as in (A)–(B_2), respectively, for ChR2-negative layer II/III pyramidal neurons. n = 10; paired t test (D), Wilcoxon test (D_1), and paired t test (D_2).

(E–F_2) Same as in (A)–(B_2), respectively, for ChR2-negative layer IV principal neurons. n = 10; paired t test.

See also Tables S3 and S5.

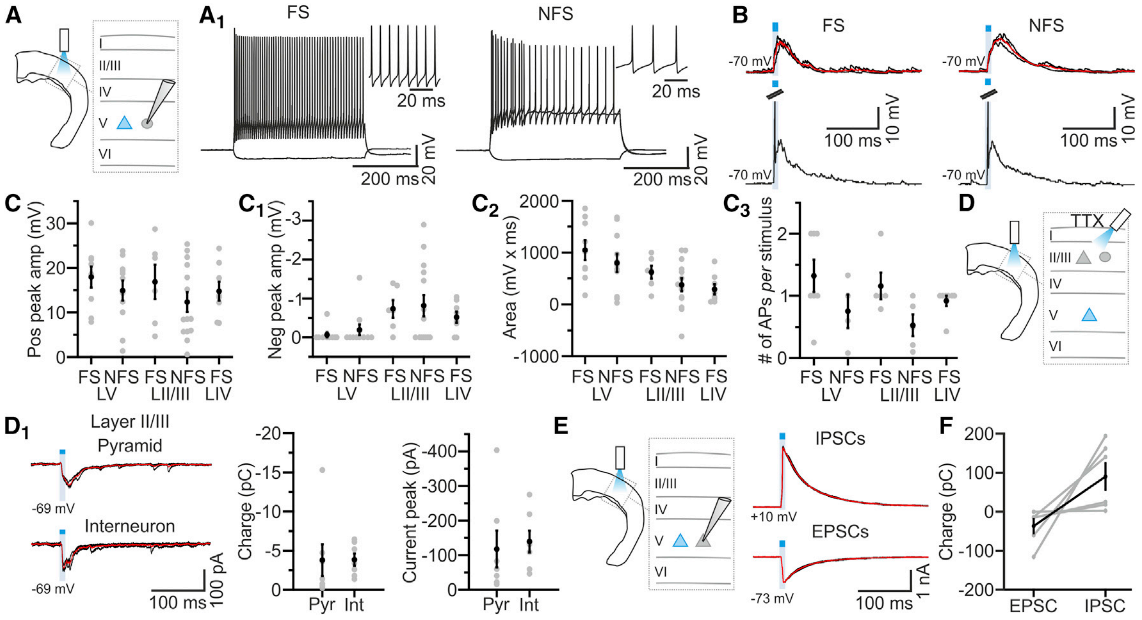

Figure 6. Layer V Recruits Inhibitory Networks across Layers

(A) Schematic of the experiment in cortical slices. The gray circle indicates ChR2-negative interneurons.

(A_1) Current injections in a layer V fast-spiking (FS; left; −100 and +600 pA) interneuron and in a non-fast-spiking (NFS; right; −100, +500, +700 pA) interneuron. APs are shown at an enlarged timescale in the insets.

(B) Top left: subthreshold responses to layer V stimulation for a layer V FS interneuron. Bottom left: a different FS interneuron showing supra-threshold response to blue light stimulation. Right: same as on the left for layer V NFS interneurons.

(C–C_3) Positive peak amplitude (C), negative peak amplitude (C_1), and area (C_2) of the light-evoked response in 9 FS and 11 NFS layer V interneurons, in 6 FS and 14 NFS layer II/III interneurons, and in 8 FS layer IV interneurons.

(C_3) The probability of firing for 7 FS and 4 NFS layer V cells, for 5 FS and 5 NFS layer II/III cells, and for 7 FS layer IV interneurons.

(D) EPSCs were recorded from layer II/III ChR2-negative pyramidal cells (gray triangle) or interneurons (gray oval) in brain slices in the presence of TTX.

(D_1) Left: light-evoked EPSCs recorded in one layer II/III ChR2-negative pyramidal neuron (top) and one layer II/III interneuron (bottom) in TTX. Middle: charge transfer under the different conditions (n = 7 pyramids and n = 7 interneurons). Right: peak EPSC amplitude for the same cells displayed in the middle panel.

(E) Left: schematic of the experiment. Right: voltage-clamp recording from a ChR2-negative layer V pyramidal neuron showing light-evoked IPSCs and EPSCs.

(F) Excitatory and inhibitory charge transfer for ChR2-negative layer V pyramidal neurons (n = 6 cells).

See also Tables S4 and S5.

an inhibitory opsin together with TdTomato. Cre recombination occurred in GABA-positive and PV-positive cells (Figures S5G and S5H). Neurons expressing the inhibitory opsin displayed a firing pattern compatible with that of PV-positive interneurons and yellow light suppressed AP firing (Figures S5I and S5J). We photostimulated layer V neurons while photoinhibiting PV-positive cells *in vivo* (Figures 7A and 7B). We found that responses of supragranular and infragranular cells to layer V activation were decreased by concurrent photoinhibition of PV interneurons (Figures 7C and 7D). Moreover, the probability of observing hyperpolarizing responses induced by layer V activation was reduced (Figure 7E). Experiments with prolonged blue light stimuli confirmed these results (Figures S7G–S7I). Importantly, in cortical slices, yellow light hyperpolarized the recorded interneuron (Figures 7F and 7G; average cell hyperpolarization: −7 ± 3 mV, n = 4), whereas blue light delivered during yellow light illumination generated membrane depolarization (average depolarization: 18 ± 4 mV, n = 4). The yellow light-induced hyperpolarization reduced spiking evoked by blue light stimuli (0.68 ± 0.13 spikes/stimulus versus 0.36 ± 0.14 spikes/stimulus, under control conditions and in the presence of yellow light, respectively; n = 17, paired t test, p = 5E-3). Photoinhibition of PV interneurons attenuated the amplitude and charge transfer of the IPSC evoked by the activation of layer V (Figures 7H–7K), confirming that PV interneurons contribute to the response of cortical neurons to layer V stimulation. Importantly, the inhibitory effect of layer V activation on whisker-evoked activity *in vivo* was reduced upon PV interneuron photoinhibition (Figures 7L–7P).

DISCUSSION

Previous optogenetic studies have begun to reveal the complex cellular interactions in supra-, infra-, and granular layers [2–6, 10–12, 44, 45]. Here, we show for the first time that firing of layer V cells tunes the temporal pattern of cortical sensory responses through intra- and trans-laminar excitation followed by PV-mediated inhibition. The temporal sharpening of the cortical responses to sensory stimulation by layer V increases the accuracy of cortical encoding of stimulus time.

Active exploration of surfaces by the animal to discriminate objects and textures elicits sudden whisker deflections whose temporal structure contributes to tactile discrimination [26, 27]. S1 neurons respond to whisker deflections with millisecond latency and with a precise temporal structure that encodes

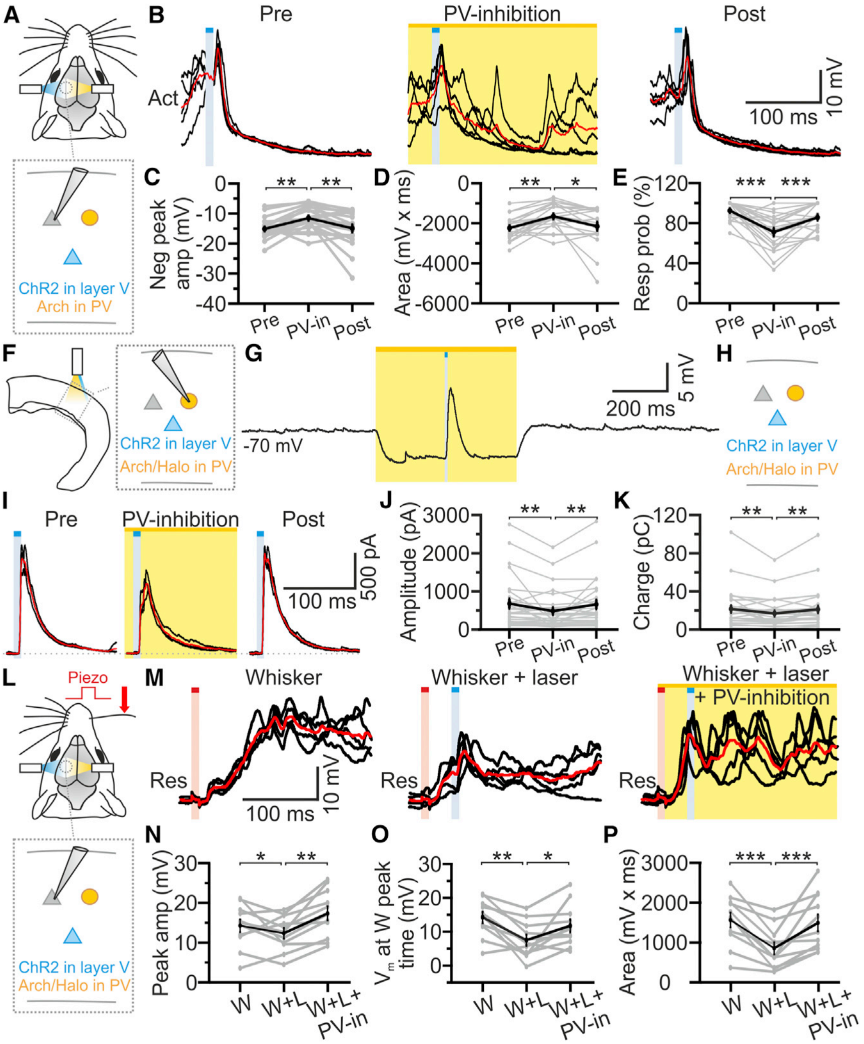

Figure 7. PV Interneurons Strongly Modulate Cortical Responses to Layer V Stimulation

(A) Schematic of the *in vivo* experiment.

(B) Recordings from a ChR2-negative principal neuron *in vivo* showing blue light stimulation alone (Pre and Post) and combined blue and yellow light stimulation (PV-inhibition) during an activated state.

(C–E) Negative peak amplitude (C), area (D), and response probability (E) of light-evoked responses under the conditions shown in (B). n = 20 cells from 10 animals; one-way repeated-measures ANOVA.

(F) Schematic of the experimental configuration in slices.

(G) A Halo-expressing PV interneuron showing cell hyperpolarization upon yellow light (yellow bar).

(H) Scheme of the brain slice experiment; recorded cells were located both in layers II/III and V.

(I) Representative examples of IPSCs evoked in recorded cells by layer V stimulation before (Pre), during (PV-inhibition), and after (Post) photo-inhibition of PV interneurons.

(J and K) Amplitude (J) and charge (K) of IPSCs under the different experimental conditions. n = 28 cells; one-way repeated-measures ANOVA.

(L) Same as in (A) and (B) but in the presence of whisker deflection.

(M) A ChR2-negative principal neuron *in vivo* showing the response to whisker stimulation (Whisker), whisker stimulation in the presence of blue light (Whisker + laser), and whisker stimulation in the presence of blue and yellow light (Whisker + laser + PV-inhibition) during an activated state.

(N–P) Positive peak amplitude (N), membrane potential value at the whisker response time peak (O), and area (P) for supragranular and infragranular ChR2-negative neurons under the different conditions shown in (M). n = 11 cells from 5 animals; one-way repeated-measures ANOVA.

See also Figures S5–S7.

information relevant to the animal's perception [28, 46]. If layer V neurons fire immediately after the initial response of whisker-coding neurons, as suggested by the response latencies observed *in vivo* [34, 35], they would suppress later firing while preserving the short-latency response that carries almost all the information about rapid whisker stimulation [47]. Such sharpening would greatly facilitate the decoding of timing of rapid whisker deflection from coincident firing in pools of S1 neurons [36] by reducing the false detection of whisker activation due to later firing. Truncating the neuronal response would also avoid the superposition of a given response with that of successive whisker deflections. By temporally tuning the cortical response to tactile stimuli, layer V neurons may therefore facilitate the encoding of sequences of rapid whisker deflections that occur during stimulation by surface textures. In a texture discrimination task, we indeed found that RTs in Hit trials were longer during optogenetic inactivation of layer V cells compared to when the light was not presented. Although significant, the effect on RTs was small (44–68 ms) compared to RTs under control conditions (989 ± 80 ms), consistent with the observation that we manipulated the activity of a minority of layer V cells ($\leq$25%; see STAR Methods and the legends of Figures S1B, S1C, and S4A–S4B$_1$). Our observation that RTs increase upon layer V inhibition is consistent with the proposed effect of layer V on sensory coding. Indeed, layer V inhibition during either passive whisker stimulation in anesthetized animals or active whisking in awake mice resulted in decreased precision of encoding of whisker stimulus time. Decreasing the precision in encoding stimulus time is expected to make it harder for the mouse to detect the presence of the sensory stimulus, in agreement with the observed increase in RTs in the discrimination task. The sensory response to single-whisker touch on the pole during active whisking was small and the effect of layer V inhibition included an increase in firing rate in the post-touch window but also in the pre-touch window (Figure 3C$_1$). This latter observation is not inconsistent with the effect of layer V inhibition on the late whisker response to passive deflection described in Figure 1I$_1$, because a delayed effect of layer V inhibition on the cortical response to whisker touch may be observed in the post-touch window of touch (*i*) but also in the pre-touch window of touch (*i* + 1) during active whisking. Future experiments will be needed to investigate the difference in the absolute value of baseline firing and whisker deflection-evoked or touch-evoked firing observed under anesthesia and in awake mice.

To dissect the cellular mechanisms underlying layer V control of cortical excitability, we first asked whether intracortical circuits were sufficient to generate the complex membrane potential responses observed in ChR2-negative cells following layer V activation. Given the widespread projection of layer V neurons to extracortical areas that in turn massively project back to the cortex (e.g., the cortico-thalamo-cortical loop), the effect of layer V stimulation could be mediated by cells located in extracortical structures. However, our findings in neocortical slices isolated from the rest of the brain demonstrated that intracortical projections are sufficient to mediate the effects of layer V stimulation. Moreover, recordings in the presence of TTX indicated that layer V directly innervates pyramids and interneuronal cells across layers.

Activation of layer V excitatory fibers heavily recruits local cortical interneurons across layers. Cortical slice experiments demonstrated that layer V stimulation evoked spiking in ~80% of FS and 40% of NFS interneurons and elicited GABAergic IPSCs in principal cells (Figures 6E and 6F). Moreover, combinatorial optogenetic experiments *in vivo* and *in vitro* showed that PV interneurons, rather than SST-positive cells, predominantly influenced the cortical responses to layer V photostimulation (Figures 7, S6, and S7). Previous findings in slice preparations [40, 41] reported a di-synaptic inhibitory pathway among layer V pyramidal neurons mediated by a subpopulation of NFS SST-positive interneurons, the Martinotti cells. Our data are not inconsistent with these reports, because we found that layer V pyramids drive firing in NFS neurons, a group of interneurons that includes the Martinotti cells. Furthermore, in slice recordings, we observed that photoinhibition of SST interneurons had a small, but significant, impact on the IPSC in principal neurons that was evoked by stimulation of layer V pyramidal cells (Figure S6J). However, *in vivo* the cortical responses to layer V stimulation were affected by photoinhibition of PV cells (Figures 7B–7E) but not SST cells (Figures S6B–S6E). Therefore, we propose that *in vivo*, PV-positive cells are the major interneuronal population contributing to the modulation of cortical excitability exerted by layer V activation. In this regard, it is interesting to note that in cortical slice recordings, we found that layer V activation efficiently drives firing in interneurons located across layers. Analogous to layer VI principal cells, which suppress cortical activity by activating layer VI inhibitory neurons whose axons span all layers [3, 4], layer V pyramidal neurons contact a population of PV-positive GABAergic neurons in layer V whose trans-laminar axons project to supra-granular layers [48, 49]. Thus, one possibility is that the hyperpolarizing responses evoked by layer V are mediated by a combination of intra- and trans-laminar inhibition.

Based on the evidence presented in this study, we propose the presence of two prominent circuits for sensory processing within a single cortical column: layer IV to layer II/III and layer V to layer II/III. Each of these two circuits can be directly activated by thalamic inputs [35, 50–52] and process information. The activity of these two parallel pathways is then integrated (1) in infra-granular layers, a major target of layer II/III axons; (2) in supra-granular layer II/III, where the axonal projections from both layer IV and layer V converge [9]; (3) through layer IV control of layer V interneurons [10]; and (4) through layer V control of layer IV cells (Figures 5, 6, and S2).

In summary, our study shows that the prototypical corticofugal layer V directly controls information encoding in the cortex via intra- and trans-laminar excitatory projections that recruit both glutamatergic and GABAergic cortical circuits. During somatosensation, layer V pyramidal neurons not only relay sensory information to higher brain areas but actively sculpt local cortical coding, leading to temporal sharpening of cortical responses to sensory inputs.

STAR★METHODS

Detailed methods are provided in the online version of this paper and include the following:

- KEY RESOURCES TABLE
- LEAD CONTACT AND MATERIALS AVAILABILITY
- EXPERIMENTAL MODEL AND SUBJECT DETAILS
  - Mice
- METHOD DETAILS
  - Viral Injections
  - Animal preparation and surgery for *in vivo* recordings
  - Slices preparation
  - Single cells electrophysiology
  - *In vivo* recordings
  - *In vitro* recordings
  - Linear probes recordings
  - Optical stimulation
  - Sensory stimulation
  - Behavioral experiments
  - Histology
- QUANTIFICATION AND STATISTICAL ANALYSIS
  - Statistical methods
  - Analysis of *in vivo* recordings
  - Analysis of *in vitro* experiments
  - Analysis of behavioral experiments
- DATA AND CODE AVAILABILITY

SUPPLEMENTAL INFORMATION

Supplemental Information can be found online at https://doi.org/10.1016/j.cub.2020.02.004.

ACKNOWLEDGMENTS

We thank F. Succol and S. Zucca for technical help; P. Farisello and M. Maravall for preliminary work; C. Gerfen for *Rbp4-cre* mice; and K. Deisseroth and E. Boyden for constructs (Addgene 20298, 26966, and 22222). This work was supported by the ERC (EU; NEURO-PATTERNS), NIH (USA; U01 NS090576), U19 NS107464), Flag-Era JTC (EU; SLOW-DYN), FP7-602531 (EU; DESIRE), and Marie Sklodowska-Curie program (EU; ETIC (699829).

AUTHOR CONTRIBUTIONS

D.V. and R.B. performed experiments and analysis. R.C. and T.B. performed behavioral experiments. F.V., D.V., M.M.-M., and S.P. performed analysis of extracellular recordings. D.V., A.F., and R.C. analyzed behavioral data. C.M. and T.F. developed DMD photostimulation. F.V., C.M., M.M.-M., A.F., and S.P. wrote software. N.B. performed preliminary optogenetic experiments in brain slices. T.F. conceived and coordinated the project. S.P. and T.F. proposed that layer V neurons encode whisker deflection time. D.V., R.B., A.H., S.P., and T.F. wrote the paper. All authors approved the final manuscript.

DECLARATION OF INTERESTS

The authors declare no competing interests.

Received: June 24, 2019
Revised: January 16, 2020
Accepted: February 3, 2020
Published: March 12, 2020

REFERENCES

1. Harris, K.D., and Mrsic-Flogel, T.D. (2013). Cortical connectivity and sensory coding. Nature *503*, 51–58.
2. Adesnik, H., and Scanziani, M. (2010). Lateral competition for cortical space by layer-specific horizontal circuits. Nature *464*, 1155–1160.
3. Olsen, S.R., Bortone, D.S., Adesnik, H., and Scanziani, M. (2012). Gain control by layer six in cortical circuits of vision. Nature *483*, 47–52.
4. Bortone, D.S., Olsen, S.R., and Scanziani, M. (2014). Translaminar inhibitory cells recruited by layer 6 corticothalamic neurons suppress visual cortex. Neuron *82*, 474–485.
5. Kim, J., Matney, C.J., Blankenship, A., Hestrin, S., and Brown, S.P. (2014). Layer 6 corticothalamic neurons activate a cortical output layer, layer 5a. J. Neurosci. *34*, 9656–9664.
6. Pluta, S., Naka, A., Veit, J., Telian, G., Yao, L., Hakim, R., Taylor, D., and Adesnik, H. (2015). A direct translaminar inhibitory circuit tunes cortical output. Nat. Neurosci. *18*, 1631–1640.
7. Wester, J.C., and Contreras, D. (2012). Columnar interactions determine horizontal propagation of recurrent network activity in neocortex. J. Neurosci. *32*, 5454–5471.
8. Feldmeyer, D. (2012). Excitatory neuronal connectivity in the barrel cortex. Front. Neuroanat. *6*, 24.
9. Feldmeyer, D., Brecht, M., Helmchen, F., Petersen, C.C., Poulet, J.F., Staiger, J.F., Luhmann, H.J., and Schwarz, C. (2013). Barrel cortex function. Prog. Neurobiol. *103*, 3–27.
10. Pluta, S.R., Telian, G.I., Naka, A., and Adesnik, H. (2019). Superficial layers suppress the deep layers to fine-tune cortical coding. J. Neurosci. *39*, 2052–2064.
11. Pauzin, F.P., and Krieger, P. (2018). A corticothalamic circuit for refining tactile encoding. Cell Rep. *23*, 1314–1325.
12. Quiquempoix, M., Fayad, S.L., Boutourlinsky, K., Leresche, N., Lambert, R.C., and Bessaih, T. (2018). Layer 2/3 pyramidal neurons control the gain of cortical output. Cell Rep. *24*, 2799–2807.e4.
13. Diamond, M.E., von Heimendahl, M., Knutsen, P.M., Kleinfeld, D., and Ahissar, E. (2008). 'Where' and 'what' in the whisker sensorimotor system. Nat. Rev. Neurosci. *9*, 601–612.
14. Hong, Y.K., Lacefield, C.O., Rodgers, C.C., and Bruno, R.M. (2018). Sensation, movement and learning in the absence of barrel cortex. Nature *561*, 542–546.
15. Knutsen, P.M., Pietr, M., and Ahissar, E. (2006). Haptic object localization in the vibrissal system: behavior and performance. J. Neurosci. *26*, 8451–8464.
16. Mehta, S.B., Whitmer, D., Figueroa, R., Williams, B.A., and Kleinfeld, D. (2007). Active spatial perception in the vibrissa scanning sensorimotor system. PLoS Biol. *5*, e15.
17. O'Connor, D.H., Peron, S.P., Huber, D., and Svoboda, K. (2010). Neural activity in barrel cortex underlying vibrissa-based object localization in mice. Neuron *67*, 1048–1061.
18. Kleinfeld, D., and Deschênes, M. (2011). Neuronal basis for object location in the vibrissa scanning sensorimotor system. Neuron *72*, 455–468.
19. Carvell, G.E., and Simons, D.J. (1990). Biometric analyses of vibrissal tactile discrimination in the rat. J. Neurosci. *10*, 2638–2648.
20. Arabzadeh, E., Zorzin, E., and Diamond, M.E. (2005). Neuronal encoding of texture in the whisker sensory pathway. PLoS Biol. *3*, e17.
21. Jadhav, S.P., and Feldman, D.E. (2010). Texture coding in the whisker system. Curr. Opin. Neurobiol. *20*, 313–318.
22. von Heimendahl, M., Itskov, P.M., Arabzadeh, E., and Diamond, M.E. (2007). Neuronal activity in rat barrel cortex underlying texture discrimination. PLoS Biol. *5*, e305.
23. Adibi, M., and Arabzadeh, E. (2011). A comparison of neuronal and behavioral detection and discrimination performances in rat whisker system. J. Neurophysiol. *105*, 356–365.
24. Lee, C.C., Diamond, M.E., and Arabzadeh, E. (2016). Sensory prioritization in rats: behavioral performance and neuronal correlates. J. Neurosci. *36*, 3243–3253.
25. Saraf-Sinik, I., Assa, E., and Ahissar, E. (2015). Motion makes sense: an adaptive motor-sensory strategy underlies the perception of object location in rats. J. Neurosci. *35*, 8777–8789.
26. Isett, B.R., Feasel, S.H., Lane, M.A., and Feldman, D.E. (2018). Slip-based coding of local shape and texture in mouse S1. Neuron *97*, 418–433.e5.
27. Jadhav, S.P., Wolfe, J., and Feldman, D.E. (2009). Sparse temporal coding of elementary tactile features during active whisker sensation. Nat. Neurosci. *12*, 792–800.
28. Zuo, Y., Safaai, H., Notaro, G., Mazzoni, A., Panzeri, S., and Diamond, M.E. (2015). Complementary contributions of spike timing and spike rate to perceptual decisions in rat S1 and S2 cortex. Curr. Biol. *25*, 357–363.
29. Ahissar, E., and Zacksenhouse, M. (2001). Temporal and spatial coding in the rat vibrissal system. Prog. Brain Res. *130*, 75–87.
30. Petersen, C.C., Hahn, T.T., Mehta, M., Grinvald, A., and Sakmann, B. (2003). Interaction of sensory responses with spontaneous depolarization in layer 2/3 barrel cortex. Proc. Natl. Acad. Sci. USA *100*, 13638–13643.
31. Crochet, S., and Petersen, C.C.H. (2006). Correlating whisker behavior with membrane potential in barrel cortex of awake mice. Nat. Neurosci. *9*, 608–610.
32. Steriade, M., Nuñez, A., and Amzica, F. (1993). A novel slow (< 1 Hz) oscillation of neocortical neurons in vivo: depolarizing and hyperpolarizing components. J. Neurosci. *13*, 3252–3265.
33. Crunelli, V., and Hughes, S.W. (2010). The slow (<1 Hz) rhythm of non-REM sleep: a dialogue between three cardinal oscillators. Nat. Neurosci. *13*, 9–17.
34. Sakata, S., and Harris, K.D. (2009). Laminar structure of spontaneous and sensory-evoked population activity in auditory cortex. Neuron *64*, 404–418.
35. Constantinople, C.M., and Bruno, R.M. (2013). Deep cortical layers are activated directly by thalamus. Science *340*, 1591–1594.
36. Stüttgen, M.C., and Schwarz, C. (2010). Integration of vibrotactile signals for whisker-related perception in rats is governed by short time constants: comparison of neurometric and psychometric detection performance. J. Neurosci. *30*, 2060–2069.
37. Panzeri, S., and Diamond, M.E. (2010). Information carried by population spike times in the whisker sensory cortex can be decoded without knowledge of stimulus time. Front. Synaptic Neurosci. *2*, 17.
38. Avermann, M., Tomm, C., Mateo, C., Gerstner, W., and Petersen, C.C. (2012). Microcircuits of excitatory and inhibitory neurons in layer 2/3 of mouse barrel cortex. J. Neurophysiol. *107*, 3116–3134.
39. Ascoli, G.A., Alonso-Nanclares, L., Anderson, S.A., Barrionuevo, G., Benavides-Piccione, R., Burkhalter, A., Buzsáki, G., Cauli, B., Defelipe, J., Fairén, A., et al.; Petilla Interneuron Nomenclature Group (2008). Petilla terminology: nomenclature of features of GABAergic interneurons of the cerebral cortex. Nat. Rev. Neurosci. *9*, 557–568.
40. Silberberg, G., and Markram, H. (2007). Disynaptic inhibition between neocortical pyramidal cells mediated by Martinotti cells. Neuron *53*, 735–746.
41. Berger, T.K., Silberberg, G., Perin, R., and Markram, H. (2010). Brief bursts self-inhibit and correlate the pyramidal network. PLoS Biol. *8*, e1000473.
42. Arenkiel, B.R., Peca, J., Davison, I.G., Feliciano, C., Deisseroth, K., Augustine, G.J., Ehlers, M.D., and Feng, G. (2007). In vivo light-induced

activation of neural circuitry in transgenic mice expressing channelrhodopsin-2. Neuron *54*, 205–218.

43. Sohal, V.S., Zhang, F., Yizhar, O., and Deisseroth, K. (2009). Parvalbumin neurons and gamma rhythms enhance cortical circuit performance. Nature *459*, 698–702.

44. Iurilli, G., Ghezzi, D., Olcese, U., Lassi, G., Nazzaro, C., Tonini, R., Tucci, V., Benfenati, F., and Medini, P. (2012). Sound-driven synaptic inhibition in primary visual cortex. Neuron *73*, 814–828.

45. Beltramo, R., D'Urso, G., Dal Maschio, M., Farisello, P., Bovetti, S., Clovis, Y., Lassi, G., Tucci, V., De Pietri Tonelli, D., and Fellin, T. (2013). Layer-specific excitatory circuits differentially control recurrent network dynamics in the neocortex. Nat. Neurosci. *16*, 227–234.

46. O'Connor, D.H., Hires, S.A., Guo, Z.V., Li, N., Yu, J., Sun, Q.Q., Huber, D., and Svoboda, K. (2013). Neural coding during active somatosensation revealed using illusory touch. Nat. Neurosci. *16*, 958–965.

47. Panzeri, S., Petersen, R.S., Schultz, S.R., Lebedev, M., and Diamond, M.E. (2001). The role of spike timing in the coding of stimulus location in rat somatosensory cortex. Neuron *29*, 769–777.

48. Buchanan, K.A., Blackman, A.V., Moreau, A.W., Elgar, D., Costa, R.P., Lalanne, T., Tudor Jones, A.A., Oyrer, J., and Sjöström, P.J. (2012). Target-specific expression of presynaptic NMDA receptors in neocortical microcircuits. Neuron *75*, 451–466.

49. Yetman, M.J., Washburn, E., Hyun, J.H., Osakada, F., Hayano, Y., Zeng, H., Callaway, E.M., Kwon, H.B., and Taniguchi, H. (2019). Intersectional monosynaptic tracing for dissecting subtype-specific organization of GABAergic interneuron inputs. Nat. Neurosci. *22*, 492–502.

50. Bureau, I., von Saint Paul, F., and Svoboda, K. (2006). Interdigitated paralemniscal and lemniscal pathways in the mouse barrel cortex. PLoS Biol. *4*, e382.

51. Petreanu, L., Mao, T., Sternson, S.M., and Svoboda, K. (2009). The subcellular organization of neocortical excitatory connections. Nature *457*, 1142–1145.

52. Meyer, H.S., Wimmer, V.C., Hemberger, M., Bruno, R.M., de Kock, C.P., Frick, A., Sakmann, B., and Helmstaedter, M. (2010). Cell type-specific thalamic innervation in a column of rat vibrissal cortex. Cereb. Cortex *20*, 2287–2303.

53. Gradinaru, V., Zhang, F., Ramakrishnan, C., Mattis, J., Prakash, R., Diester, I., Goshen, I., Thompson, K.R., and Deisseroth, K. (2010). Molecular and cellular approaches for diversifying and extending optogenetics. Cell *141*, 154–165.

54. Chow, B.Y., Han, X., Dobry, A.S., Qian, X., Chuong, A.S., Li, M., Henninger, M.A., Belfort, G.M., Lin, Y., Monahan, P.E., and Boyden, E.S. (2010). High-performance genetically targetable optical neural silencing by light-driven proton pumps. Nature *463*, 98–102.

55. Clack, N.G., O'Connor, D.H., Huber, D., Petreanu, L., Hires, A., Peron, S., Svoboda, K., and Myers, E.W. (2012). Automated tracking of whiskers in videos of head fixed rodents. PLoS Comput. Biol. *8*, e1002591.

56. Quiroga, R.Q., Nadasdy, Z., and Ben-Shaul, Y. (2004). Unsupervised spike detection and sorting with wavelets and superparamagnetic clustering. Neural Comput. *16*, 1661–1687.

57. Forli, A., Vecchia, D., Binini, N., Succol, F., Bovetti, S., Moretti, C., Nespoli, F., Mahn, M., Baker, C.A., Bolton, M.M., et al. (2018). Two-photon bidirectional control and imaging of neuronal excitability with high spatial resolution *in vivo*. Cell Rep. *22*, 3087–3098.

58. De Stasi, A.M., Farisello, P., Marcon, I., Cavallari, S., Forli, A., Vecchia, D., Losi, G., Mantegazza, M., Panzeri, S., Carmignoto, G., et al. (2016). Unaltered network activity and interneuronal firing during spontaneous cortical dynamics in vivo in a mouse model of severe myoclonic epilepsy of infancy. Cereb. Cortex *26*, 1778–1794.

59. Zucca, S., Pasquale, V., Lagomarsino de Leon Roig, P., Panzeri, S., and Fellin, T. (2019). Thalamic drive of cortical parvalbumin-positive interneurons during down states in anesthetized mice. Curr. Biol. *29*, 1481–1490.e6.

60. Harrison, T.C., Sigler, A., and Murphy, T.H. (2009). Simple and cost-effective hardware and software for functional brain mapping using intrinsic optical signal imaging. J. Neurosci. Methods *182*, 211–218.

61. Gentet, L.J., Avermann, M., Matyas, F., Staiger, J.F., and Petersen, C.C. (2010). Membrane potential dynamics of GABAergic neurons in the barrel cortex of behaving mice. Neuron *65*, 422–435.

62. Zucca, S., D'Urso, G., Pasquale, V., Vecchia, D., Pica, G., Bovetti, S., Moretti, C., Varani, S., Molano-Mazón, M., Chiappalone, M., et al. (2017). An inhibitory gate for state transition in cortex. eLife *6*, 26177.

63. Lefort, S., Tomm, C., Floyd Sarria, J.C., and Petersen, C.C. (2009). The excitatory neuronal network of the C2 barrel column in mouse primary somatosensory cortex. Neuron *61*, 301–316.

64. Helmstaedter, M., de Kock, C.P., Feldmeyer, D., Bruno, R.M., and Sakmann, B. (2007). Reconstruction of an average cortical column in silico. Brain Res. Brain Res. Rev. *55*, 193–203.

65. Holtmaat, A., Bonhoeffer, T., Chow, D.K., Chuckowree, J., De Paola, V., Hofer, S.B., Hübener, M., Keck, T., Knott, G., Lee, W.C., et al. (2009). Long-term, high-resolution imaging in the mouse neocortex through a chronic cranial window. Nat. Protoc. *4*, 1128–1144.

66. Chen, J.L., Carta, S., Soldado-Magraner, J., Schneider, B.L., and Helmchen, F. (2013). Behaviour-dependent recruitment of long-range projection neurons in somatosensory cortex. Nature *499*, 336–340.

67. Chuong, A.S., Miri, M.L., Busskamp, V., Matthews, G.A., Acker, L.C., Sørensen, A.T., Young, A., Klapoetke, N.C., Henninger, M.A., Kodandaramaiah, S.B., et al. (2014). Noninvasive optical inhibition with a red-shifted microbial rhodopsin. Nat. Neurosci. *17*, 1123–1129.

68. Mahn, M., Prigge, M., Ron, S., Levy, R., and Yizhar, O. (2016). Biophysical constraints of optogenetic inhibition at presynaptic terminals. Nat. Neurosci. *19*, 554–556.

69. Mateo, C., Avermann, M., Gentet, L.J., Zhang, F., Deisseroth, K., and Petersen, C.C. (2011). *In vivo* optogenetic stimulation of neocortical excitatory neurons drives brain-state-dependent inhibition. Curr. Biol. *21*, 1593–1602.

70. Petersen, R.S., Panzeri, S., and Diamond, M.E. (2001). Population coding of stimulus location in rat somatosensory cortex. Neuron *32*, 503–514.

71. Mazurek, M.E., and Shadlen, M.N. (2002). Limits to the temporal fidelity of cortical spike rate signals. Nat. Neurosci. *5*, 463–471.

72. Mikula, S., and Niebur, E. (2003). The effects of input rate and synchrony on a coincidence detector: analytical solution. Neural Comput. *15*, 539–547.

73. Quian Quiroga, R., and Panzeri, S. (2009). Extracting information from neuronal populations: information theory and decoding approaches. Nat. Rev. Neurosci. *10*, 173–185.

74. Benjamini, Y., and Hochberg, Y. (1995). Controlling the false discovery rate: a practical and powerful approach to multiple testing. J. R. Stat. Soc. Series B Stat. Methodol. *57*, 289–300.

75. Benjamini, Y., and Yekutieli, D. (2001). The control of the false discovery rate in multiple testing under dependency. Ann. Stat. *29*, 1165–1188.

76. Pettersen, K.H., Devor, A., Ulbert, I., Dale, A.M., and Einevoll, G.T. (2006). Current-source density estimation based on inversion of electrostatic forward solution: effects of finite extent of neuronal activity and conductivity discontinuities. J. Neurosci. Methods *154*, 116–133.

77. Belitski, A., Gretton, A., Magri, C., Murayama, Y., Montemurro, M.A., Logothetis, N.K., and Panzeri, S. (2008). Low-frequency local field potentials and spikes in primary visual cortex convey independent visual information. J. Neurosci. *28*, 5696–5709.

78. Reyes-Puerta, V., Sun, J.J., Kim, S., Kilb, W., and Luhmann, H.J. (2015). Laminar and columnar structure of sensory-evoked multineuronal spike sequences in adult rat barrel cortex *in vivo*. Cereb. Cortex *25*, 2001–2021.

79. Cauli, B., Audinat, E., Lambolez, B., Angulo, M.C., Ropert, N., Tsuzuki, K., Hestrin, S., and Rossier, J. (1997). Molecular and physiological diversity of cortical nonpyramidal cells. J. Neurosci. *17*, 3894–3906.

STAR★METHODS

KEY RESOURCES TABLE

REAGENT or RESOURCE	SOURCE	IDENTIFIER
Antibodies		
Rabbit anti-GABA unconjugated	Sigma-Aldrich	Cat# A2052; RRID: AB_447652
Rat monoclonal anti-somatostatin	Millipore	Cat# MAB354; RRID: AB_2255365
Mouse monoclonal anti-parvalbumin	Sigma-Aldrich	Cat# P3088; RRID: AB_477329
Mouse anti-NeuN	Millipore	Cat# MAB377; RRID: AB_2298772
Bacterial and Virus Strains		
AAV.EF1a.DIO.hChR2(H134R)-eYFP.WPRE.hGH	Penn Vector Core	Addgene viral prep # 20298-AAV1
AAV.EF1a.DIO.eNpHR3.0-eYFP.WPRE.hGH	Penn Vector Core, see [53]	Addgene viral prep # 26966-AAV1 and –AAV9
AAV.CBA.Flex.Arch-GFP.WPRE.SV40	Penn Vector Core, see [54]	Addgene viral prep # 22222-AAV1 and –AAV9
AAV.CAG.Flex.tdTomato.WPRE.bGH	Penn Vector Core	AllenInstitute864
Chemicals, Peptides, and Recombinant Proteins		
bisBenzimide H 33342 trihydrochloride (Hoechst)	Sigma-Aldrich	Cat# B2261; CAS: 23491-52-3
Lidocaine *N*-ethyl bromide (QX-314 bromide)	Sigma-Aldrich	Cat# L5783; CAS: 21306-56-9
SR95531 hydrobromide (Gabazine)	Tocris	Cat# 1262; CAS: 104104-50-9
DNQX disodium salt	Tocris	Cat# 2312; CAS: 1312992-24-7
NBQX disodium salt	Tocris	Cat# 1044; CAS: 479347-86-9
D-AP5	Tocris	Cat# 0106; CAS: 79055-68-8
Muscimol, BODPY TMR-X conjugate	Thermo Fisher Scientific	M23400
Cytochrome-c	Sigma-Aldrich	Cat# C2506; CAS: 9007-43-6
Catalase	Sigma-Aldrich	Cat# C9322; CAS: 9001-05-2
3,3′-Diaminobenzidine tetrahydrochloride hydrate	Sigma-Aldrich	Cat# D5637; CAS: 868272-85-9
VECTASTAIN® Elite ABC-Peroxidase Kits Standard	Vector Laboratories	Cat# PK-6100
DAB Peroxidase Substrate Kit	Vector Laboratories	Cat# SK-4100; CAS: 7411-49-6 and CAS: 7791-20-0
Critical Commercial Assays		
Kwik-Cast	World Precision Instruments	Cat# KWIK-CAST
Stepper motor	Zaber	Cat# T-NM17A04
Motorized linear stage	Zaber	Cat# T-LSM100A
Experimental Models: Organisms/Strains		
Mouse: Tg(Rbp4-cre)KL100Gsat/Mmcd	GENSAT	RRID: MMRRC_031125-UCD
Mouse: B6;129S6-*Gt(ROSA)26Sor*$^{tm14(CAG\text{-}TdTomato)Hze}$/J	The Jackson Laboratory	RRID: IMSR_JAX:007908
Mouse: B6;129P2-*Pvalb*$^{tm1(cre)Arbr}$/J	The Jackson Laboratory	RRID: IMSR_JAX:008069
Mouse: *Sst*$^{tm2.1(cre)Zjh}$/J	The Jackson Laboratory	RRID: IMSR_JAX:013044
Mouse: B6.Cg-Tg(Thy1-COP4/EYFP)18Gfng/J	The Jackson Laboratory	RRID: IMSR_JAX:007612
Software and Algorithms		
pClamp 10 Software Suite (v10.2 and v10.4)	Molecular Devices	https://www.moleculardevices.com; RRID: SCR_011323
Cheetah (v5.0)	NeuraLynx	https://neuralynx.com/software/cheetah
LabVIEW	National Instruments	http://ni.com; RRID: SCR_014325
Neurolucida	MBF Bioscience	RRID: SCR_001775
MATLAB (version 2014b)	Mathworks	http://www.mathworks.com; RRID: SCR_001622
Whisk	See [55]	https://www.janelia.org/open-science/whisk-whisker-tracking
ImageJ	Fiji	https://fiji.sc/
Wave_Clus	See [56]	N/A
GraphPad PRISM (v5.0)	GraphPad PRISM	http://www.graphpad.com/; RRID: SCR_002798

(*Continued on next page*)

Continued		
REAGENT or RESOURCE	SOURCE	IDENTIFIER
Other		
Piezoelectric bimorph stimulator	Physik Instrumente	Cat# PL140.11
16 channel linear silicon probe	NeuroNexus	Cat# A1x16-3mm-50-177-Z16
MultiClamp 700B amplifier	Molecular Devices	https://www.moleculardevices.com
Axon Digidata 1440A	Molecular Devices	https://www.moleculardevices.com
Axon Digidata 1550	Molecular Devices	https://www.moleculardevices.com
Digital Lynx SX	NeuraLynx	https://neuralynx.com
Basler ace camera	Basler AG	Cat# acA800-510um

LEAD CONTACT AND MATERIALS AVAILABILITY

This study did not generate new unique reagents. Any further information and requests should be directed to and will be fulfilled by the Lead Contact, Tommaso Fellin (tommaso.fellin@iit.it).

EXPERIMENTAL MODEL AND SUBJECT DETAILS

Mice

All experiments involving living animals were approved by the National Council on Animal Care of the Italian Ministry of Health (authorization # 34/2015-PR and 125/2012-B) and carried out in accordance with the guidelines established by the European Communities Council Directive. The mouse strain Tg(Rbp4-cre)KL100Gsat/Mmcd (otherwise called *Rbp4-cre*), identification number 031125-UCD, was obtained from the Mutant Mouse Regional Resource Center, a NCRR-NIH funded strain repository, and was donated to the MMRRC by the NINDS-funded GENSAT BAC transgenic project. B6;129S6-*Gt(ROSA)26Sor*$^{tm14(CAG\text{-}TdTomato)Hze}$/J, id #007908 (otherwise called *TdTomato* line), B6;129P2-*Pvalb*$^{tm1(cre)Arbr}$/J, id #008069 (*PV-cre* line), STOCK *Sst*$^{tm2.1(cre)Zjh}$/J, id #013044 (*SST-cre* line) and B6.Cg-Tg(Thy1-COP4/EYFP)18Gfng/J line 18, id #007612 (*Thy1-ChR2* line) were purchased from the Jackson Laboratory (Bar Harbor, USA). All data were collected from mice of either sex. From postnatal days 30, animals were separated from the original cage and housed in group of up to four littermates *per* cage with *ad libitum* access to food and water in a 12:12 light-dark cycle. Age of animals used for each experimental dataset is specified in Method Details. The number of animals used for each experimental dataset is specified in the text or in the corresponding Figure legend.

METHOD DETAILS

Viral Injections

Rbp4-cre x *TdTomato* mice were injected with AAV.EF1a.DIO.hChR2(H134R)-eYFP.WPRE.hGH or AAV.EF1a.DIO.eNpHR3.0-eYFP.WPRE.hGH; *SST-cre* or *PV-cre* x *Thy1-ChR2* mice were injected with a combination of AAV.CBA.Flex.Arch-GFP.WPRE.SV40 and AAV.CAG.Flex.tdTomato.WPRE.bGH (1:1) or AAV.EF1a.DIO.eNpHR3.0-eYFP.WPRE.hGH and AAV.CAG.Flex.tdTomato.WPRE.bGH (1:1). For experiments in Figures S5A–S5C and S5F–S5H, *SST-cre* or *PV-cre* x *Thy1-ChR2* mice were injected with AAV.CAG.Flex.tdTomato.WPRE.bGH. All viral injections were performed at the date of birth (P0-P2) similarly to [57, 58], unless otherwise stated, and adeno-associated viruses (AAVs) were purchased from the University of Pennsylvania Viral Vector Core. Newborn mice were deeply anesthetized by hypothermia and immobilized in a refrigerated custom stereotaxic apparatus. A volume of $\sim$ 250 nL of viral suspension was gradually injected at stereotaxic coordinates of 0 mm from bregma, 1.5 mm lateral to the sagittal sinus and 0.3 mm depth, by means of a glass micropipette. After the injection, the micropipette was gently removed and the skin was sutured. The pups were quickly revitalized under a heat lamp and subsequently returned to the cage.

Animal preparation and surgery for *in vivo* recordings

Anesthetized mice

Mice (age > 24 postnatal days) were anesthetized with an intraperitoneal injection of urethane (16.5%, 1.65 g*kg^{-1}) and placed on a stereotaxic apparatus. Body temperature was measured with a rectal probe and kept at 37°C with a heating pad. The depth of anesthesia was assured by monitoring respiration rate, heartbeat, eyelid reflex, vibrissae movements, reactions to tail and toe pinching. Oxygen saturation was controlled by a pulse oximeter (MouseOx, Starr Life Sciences Corp., Oakmont, PA). 2% lidocaine solution was injected under the skin before surgical incision. A small craniotomy (500 × 500 μm) was performed over S1 and, after the removal of the bone, the surface of the brain was continuously kept moist with normal HEPES-buffered artificial cerebrospinal fluid (ACSF). The dura was removed only for extracellular recordings experiments with linear silicon probes (Figures S4K–S4R).

For experiments with sensory stimulation (Figures 1, 2, 3, 7L–7P, S3L–S3Q_2, and S4), the craniotomy was targeted to the barrel field of S1 using custom built set-up for intrinsic optical imaging similarly to [59]. Intrinsic optical imaging was performed in mice in which the skull over the entire barrel field was thinned and illuminated with red light (wavelength: 630 ± 10 nm). Reflected light was collected with a camera (Hamamatsu, Milan, Italy). Most or all whiskers except the target one (usually C1 or C2 or D1) were trimmed. The targeted whisker was placed inside a glass pipette connected to a piezoelectric bender actuator (Physik Instrumente, Milan, Italy). The whisker was deflected at 18 Hz for 1.1 s every ~20 s for 40 trials. Camera frames were averaged over trials and image analysis was performed following [60]. The barrel corresponding to the stimulated whisker was identified as the region showing decreased reflectance relative to baseline. An image of the vasculature was taken with 546 nm illumination as spatial reference.

Awake head-fixed mice

For *in vivo* awake experiments, mice older than 6 weeks were used. Two-three weeks before recordings a custom head plate was implanted with dental cement posterior to S1 under isofluorane anesthesia (2.0%). The exposed bone was covered using the silicone elastomer Kwik-Cast (World Precision Instruments, Friedberg, Germany) and an intraperitoneal injection of antibiotic (BAYTRIL, Bayer, Germany) was given to prevent infection. Starting from two-three day after surgery, animals were habituated to head-restraint, pole presentation, and yellow light presentation sitting in a plastic tube (Figures S3H–S3K_2) or running on a free-spinning wheel (Figures 3, S4B, and S4B_1) as described in [6, 61] for a minimum of 7-10 days. Training periods gradually increased in duration each day, starting from a first head-restrained session lasting a few minutes. After completing the training, animals sat quietly in the recording environment (Figures S3H–S3K_2) or ran on the wheel (Figures 3, S4B, and S4B_1). Before electrophysiological recording, mice were anesthetized using 1.5%–2% isoflurane and a craniotomy was performed as described above. A minimum of 30 minutes was given to the mice to recover from anesthesia before the beginning of the recording session.

Slices preparation

Acute cortical coronal slices of S1 were made from P18-P36 animals. Mice were anesthetized with urethane (16.5%, 1.65 g*kg^{-1}) and decapitated. The brain was quickly dissected and placed in an ice-cold cutting solution containing: 130 mM Kgluconate, 15 mM KCl, 0.2 mM EGTA, 20 mM HEPES, and 25 mM glucose, with pH adjusted to 7.4 with NaOH and oxygenated with O_2 100%. Slices (slice thickness: 300 μm) were cut with a vibratome (VT1000S, Leica Microsystems, GmbH, Wetzlar, Germany) and after removing the subcortical areas, slices were immersed for 1 min in solution at room temperature (RT) containing: 225 mM D-mannitol, 25 mM glucose, 2.5 KCl, 1.25 NaH_2PO_4, 26 $NaHCO_3$, 0.8 mM $CaCl_2$, 8 mM $MgCl_2$, pH 7.4 with 95% O_2/5% CO_2. Slices were then incubated for 30 min at 35°C in standard ACSF (sACSF) composed of: 125 mM NaCl, 2.5 mM KCl, 25 mM $NaHCO_3$, 1.25 mM NaH_2PO_4, 2 mM $MgCl_2$, 1 mM $CaCl_2$, 25 mM glucose, pH 7.4 with 95% O_2/ 5% CO_2 and then maintained at RT.

Single cells electrophysiology

In both *in vivo* and *in vitro* experiments, the electrical signals were recorded through a Multiclamp 700B patch-clamp amplifier, digitized using a Digidata 1440 interface and analyzed with pClamp 10.2 or 10.4 software (Molecular Device, Sunnyvale, CA). *In vivo* and *in vitro* current-clamp recordings were sampled at 50 kHz and filtered at 10 kHz. *In vitro* voltage-clamp recordings were sampled at 10 kHz and filtered at 2 kHz.

In vivo recordings

Patch-clamp current-clamp recordings were performed as previously described [62]. In brief, 3-5 MΩ glass pipettes (Hilgenberg, Malsfeld, Germany) were filled with an internal solution containing (in mM): K-gluconate 140, $MgCl_2$ 1, NaCl 8, Na_2ATP 2, Na_3GTP 0.5, HEPES 10, Tris-phosphocreatine 10 to pH 7.2 with KOH. In some experiments biocytin (3 mg*ml^{-1}) was added to the solution for post hoc anatomical identification. Data were not corrected for liquid junction potential. In all patch-clamp recordings, an average of > 40 consecutive acquisitions (acquisition duration: 2-5 s) were performed under each experimental condition. For juxtasomal recordings (acquisition duration: 4-5 s), the pipettes were filled with HEPES-buffered ACSF. Data were acquired at 50 kHz and filtered at 10 kHz. Throughout the experimental session, the craniotomy was covered with normal HEPES-buffered ACSF. The depth of the recorded cortical cells was inferred from the position of the glass pipette with respect to the brain surface or, in some cases, determined by post hoc anatomical identification. Deep cells belonged to infragranular layers, either layer V (600 – 890 μm) or VI (890 – 1100 μm). Deep recorded cells were assigned to layer V or VI only if they were successfully filled and identified *a posteriori*. Cells were considered located in deep layers when the glass pipette reached a depth > of 600 μm [6]. Superficially recorded cells (140-365 μm from the pial surface) were assigned to supragranular layer II/III even in the absence of morphological reconstruction. Layer IV cells were located at depth 400 - 600 μm and identified *a posteriori*. Cells in Figures 1A–C_1 were principal ChR2-negative recorded in layers II/III, V and VI. Cells in Figures 1D–1J, 2, 3, and S4C–S4J were principal opsin-negative neurons located in deep layers. Cells in Figures S3A–S3K_2 were located in supragranular layers. Cells in Figures 7, S6, and S7D–S7I were supragranular and infragranular ChR2-negative principal cells.

In vitro recordings

Slices were transferred to a submerged recording chamber (RC26, Warner Instruments, Hamden, CT, USA) and continuously perfused with fresh bathing solution (in mM: 125 NaCl, 2.5 KCl, 1.25 NaH_2PO_4, 25 $NaHCO_3$, 2 $MgCl_2$, 2 $CaCl_2$, 25 glucose, pH 7.4 with 95%O_2/5% CO_2). Bath temperature was maintained at 30–32°C by an inline solution heater and temperature controller (TC-344B, Warner Instruments, Hamden, CT, USA). Recorded neurons were visually identified with an infrared differential interference

contrast microscopy (IR DIC) and a 40X water-immersion objective. Pipettes (tip resistance, 3–4 MΩ) were filled with internal solution containing (in mM): K-gluconate 140, $MgCl_2$ 1, NaCl 8, Na_2ATP 2, Na_3GTP 0.5, HEPES 10, Tris-phosphocreatine 10 to pH 7.2 with KOH. For recordings in Figures 6E, 6F, 7I–7K, and S6I–S6K of excitatory and inhibitory postsynaptic currents (EPSCs and IPSCs, respectively) the following solution was used (in mM): Cs-methansulfonate 145, NaCl 8, Na_2ATP 2, Na_3GTP 0.5, EGTA 0.3, HEPES 10, Tris-phosphocreatine 10 and Qx-314 bromide 5 (Sigma Aldrich, Saint Louis, MO) to pH 7.25 with CsOH. In some experiments biocytin (2-3 mg*ml^{-1}) was added to the intracellular solution for post hoc morphological identification. Series resistance (range 6-20 MΩ) was not compensated and data were not corrected for the liquid junction potential. In all the recordings obtained with the K-based intracellular solution, neurons were held at −70 mV, unless otherwise stated. The input resistance and the firing pattern of the recorded neurons were evaluated observing cellular responses to current injections (duration, 400 ms; initial current injection value, −100 pA; increasing current step, 50 pA, Table S5). In all photostimulation experiments, consecutive trials (3-20) were recorded at 0.2 Hz, except for Figures 7H–7K and S6H–S6K (0.1 Hz). In experiments displayed in Figure 6, interneurons were targeted using the IR DIC based on their round or fusiform cell body and the absence of clear apical dendrites. Pyramidal cells and interneurons were further distinguished based on their electrophysiological properties (see Analysis of *In Vitro* Experiments for details). In the experiments in Figures 6D and $6D_1$ the excitatory postsynaptic currents (EPSCs) were recorded clamping the neurons at −69 mV, corresponding to the reversal potential for Cl^- ions. In the recordings in which a Cs-based intracellular solution was used (Figures 6E, 6F, 7H–7K, and S6H–S6K), the EPSCs and IPSCs were isolated holding the cells at −73 mV and + 10 mV, the reversal potentials of inhibition and excitation which were calculated and experimentally established, respectively. Light-evoked IPSCs were completely blocked by the application of 12.5 μM gabazine (average charge transfer: control, 21.2 ± 6.6 pC; gabazine, 0.2 ± 0.4 pC, n = 11. Paired t test, p = 0.008), confirming the GABAergic nature of these currents. For experiments in Figures 7F, 7G, S1G–S1L, S5D, S5E, S5I, S5J, S6F, and S6G cells were identified based on TdTomato fluorescence. DNQX, D-AP5, TTX, NBQX and gabazine were purchased from Tocris (Bristol, UK).

Linear probes recordings

A 16-channel linear silicon probe (A1x16-3mm-50-177-Z16, NeuroNexus, Ann Arbor, MI) was slowly inserted to the desired depth using a micromanipulator (maximal probe depth: 1000-1050 μm). Electrical recording started 30 minutes after probe insertion. Throughout the experimental session, the craniotomy was covered with normal HEPES-buffered ACSF. Data were acquired with Cheetah 5 (Digital Lynx SX, NeuraLynx, Dublin, Ireland) in the 0.1 – 9000 Hz range and were sampled at 32 kHz. Electrode depth was confirmed *a posteriori* by performing the inverse current source density (iCSD) analysis during sensory stimulation and identifying a sink corresponding to layer IV (see Linear Probe Recordings in Anesthetized Mice for details). At least 60 trials (trial duration: 5 s) were acquired under each experimental condition.

Optical stimulation

Blue (λ = 473, 488 or 491 nm; stimulus duration 10 ms, unless otherwise stated) and yellow (λ = 594 nm; stimulus duration 500 ms) light illumination were performed using continuous wave, solid-state laser sources (CNI, Changchun, China; World Star Tech, Toronto, Canada; Cobolt, Vretenvägen, Sweden). Light pulses were delivered via an optical fiber (fiber diameter: 200 μm, amsTechnologies, Milan, Italy) or, in case of structured illumination, via a 10X object (Olympus, Tokyo, Japan) coupled to a DMD (see below). Blue light was presented at intensity of 0.13-18 mW. Yellow light was presented at intensity of ∼30 mW for *in vivo* experiments and 3-30 mW for *in vitro* recordings. Light power was measured at the fiber tip or underneath the objective (for recordings using DMD).

In *in vivo* recordings the fiber optic was positioned above the cortical area of interest and close to the pial surface such that the illuminated area comprised the region where the electrophysiological recording was performed. In Figures 1A–$1C_1$, 7L–7P, and S3L–$S3Q_2$ blue light was delivered 21-35 ms after the onset of the whisker-evoked depolarization. In the *in vitro* experiments displayed in Figures 5, 6A–$6C_3$, 7H–7K, S1G–S1L, and S6H–S6K, the fiber optic was placed close to the slice surface at the border between layer V and layer IV. In the *in vitro* experiments displayed in Figures 6D and $6D_1$ the fiber optic was placed in layer II/III. The somata of patched neurons in layer II/III were located within the cortical column illuminated by the fiber tip. In dual color optogenetic experiments *in vivo*, two fibers were used and were oriented in order to illuminate the same cortical area. In brain slice experiments in Figures 7F, 7G, S6F and S6G the blue and yellow lasers were delivered through a single optic fiber (fiber diameter: 200 μm). In experiments displayed in Figures 7H–7K and S6H–S6K, blue and yellow light were delivered through two independent fibers (200 μm and 1000 μm in diameter, respectively).

For patterned illumination (Figures S3A–$S3G_2$) a 473 nm laser beam (CNI, Changchun, China) passed through an acousto-optic modulator (AOM, Gooch & Housego, USA) and neutral density filters (Thorlabs, Newton, NJ). The beam was expanded by a first telescope using achromatic doublet lenses (L_1 and L_2; L_1, f = 35 mm; L_2, f = 150 mm) to impinge on the active window of the Vialux DMD (DLP 7000, Texas Instruments, Dallas, TX) with an angle of −24° with respect to the direction normal to the DMD active window [62]. The ON axis component of the modulated beam (exiting at 0° with respect to the direction normal to the DMD active window) was then relayed by a series of lenses (L_3, focal distance, f = 100 mm; L_4, f = 60 mm; L_5, f = 100 mm) and a 10X microscope objective (UPlanFLN 10x 0.3NA, Olympus, Tokyo, Japan) to the sample. The OFF axis was directed to a beam dumper at an angle of +48° with respect to the direction normal to the DMD active window. A dichroic mirror was positioned between L_5 and the microscope objective (Di01-R404/488/594, Semrock, Rochester, NY). Fluorescence was collected through a lens (L_6, f = 180 mm) by a camera (ORCA-Flash4.0, Hamamatsu, Hamamatsu, Japan) with an appropriate emission filter in front of it. The DMD was controlled using custom-made software using LabVIEW (National Instruments, Austin, TX), which managed the communication with the Vialux driving

board using the ALP-4.1 controller suite dynamic link libraries. The ALP 4.1 Advanced Programming Interface (API) allowed loading the patterns dynamically to an on-board memory, setting triggers and stimulation time, and managing other driver functionalities. A simple calibration routine was performed projecting a square pattern, adapting it to the pre-calibrated camera field of view, and retrieving the mapping parameters between DMD and sample plane. Patterned illumination was performed on a field of view of dimensions 400 × 400 μm^2 (Figures S3A–S3G_2) or 200 × 200 μm^2. For the 400 × 400 μm^2 field of view, stimulation protocols consisted in a train of 5 consecutive patterns in which individual squares (lateral dimension, 12 μm) were randomly positioned in order to cover 1/5 of the field of view. Each pattern was projected for 2 ms; total train duration was 10 ms. Each patterned illumination session was accompanied by a wide field stimulation session that was performed using the DMD and illuminating the whole field of view. Total power delivered to the sample in the wide field or patterned mode was constant (1.9 mW).

In both excitatory and inhibitory optogenetic experiments, we accurately controlled the optical stimulation, using a small-diameter fiber optic (diameter: 200 μm) with low numerical aperture (NA: 0.22), placed almost adjacent to the surface of the brain to minimize the divergence of the uncollimated light beam exiting the fiber tip. Under these conditions, we estimated that the net illuminated cortical surface had a maximal diameter comparable to a single barrel column (~300 μm) [63]. This was verified in excitatory optogenetic experiments using wide field stimulation with DMDs, in which the area of illumination could be precisely controlled down to 200 × 200 μm^2. Assuming that ~16% of layer V neurons expressed the opsin (see Figures S1A–S1E) and that ~1300 layer V neurons were contained within a barrel-related column [63], we estimated that our brief blue light stimulus resulted in the generation of 1-2 APs in ~200 cells. This level of firing activity is reached under many physiological conditions, including whisker deflection [64]. Most importantly, in our inhibitory optogenetic experiments we reduced the probability of firing in a similar number of layer V opsin-positive cells (~200), given that 20 out of 90 juxtasomally recorded deep cells in anesthetized mice and 16 out of 64 deep cells in awake mice (22% and 25%, respectively, similar to the 16% value estimated from Figures S1A–S1E) were silenced during 500 ms yellow light illumination in more than 90% of the trials in mice expressing the inhibitory opsin. However, only a fraction of these inhibitory opsin-positive neurons respond to the whisker stimulus. In fact, from our juxtasomal electrophysiological recordings in anesthetized and awake mice we estimated that ~30%–50% of recorded infragranular neurons responded with increased firing rate to the whisker deflection.

Sensory stimulation

For electrophysiological recordings in anesthetized mice, whisker deflection (duration: 10 ms, 0.2 Hz) was performed placing the targeted whisker inside a glass pipette attached to a piezoelectric stepper. The glass was positioned at ~0.8 cm away from the mouse's face and the piezoelectric bender actuator was moved in the anterior-posterior direction. For the experiments in awake mice (Figures 3, S4B, and S4B_1), mice were head-fixed on a custom wheel (Ø: 8 cm; width: 6.4 cm; fabricated using a FFF 3D printer). A vertical bar (a needle with gauge number 20) was presented perpendicular to whisking motion at ~0.8-1 cm from the whisker pad. The pole quickly moved in and out of the whisker field using a custom linear actuator controlled via Arduino Uno and synchronized with electrophysiology data via external triggers. The pole stayed still in the whisker field for 2 s at 0.25 Hz. Simultaneous high-speed videography was performed to track whisker movements and contacts as described in Whisker Imaging and Tracking.

Behavioral experiments

Surgery for cranial window implantation

All procedures were conducted in accordance with the guidelines of the Veterinary Office of Switzerland and in agreement with the veterinary office of the Canton of Geneva (license number GE/74/18). The stereotaxic viral injections were carried out on five 6-week-old *Rbp4-cre* mice. A mix of O_2 and 4% isoflurane at 0.4 l/min was used to induce anesthesia followed by an intraperitoneal injection of MMF solution consisting of 0.2 mg/kg medetomidine (Dormitor, Orion Pharma, Hamburg, DE), 5 mg/kg midazolam (Dormicum, Roche, Basel, CH), and 0.05 mg/kg fentanyl (Fentanyl, Sintetica, Mendrisio, CH) together in sterile 0.9% NaCl. AAV.EF1a.DIO.eNpHR3.0-eYFP.WP.hGH (300 nl) was delivered to the barrel cortex (1.4 mm posterior, 3.5 mm lateral from bregma). A 3 mm diameter cranial window was implanted, as described previously [65]. After this procedure, a metal post was implanted lateral to the window using dental acrylic to restrict head movement during behavior.

Habituation and water deprivation

Animals were housed with an inverted light-dark cycle 7-8 days before the first pre-training session. All experiments were performed during the dark phase. Two weeks after surgery, mice were handled and accustomed to be head restrained on the training setup for 10-15 min during 4-5 days. Water deprivation started 3-5 days before the first pre-training session and ceased at the end of the training. Weight was monitored daily during this period and the amount of water given was adjusted to prevent them from losing more than 15% of their original weight. Altogether, mice received a minimum of 1 mL of water *per* day corresponding to the amount they drank during the training as rewards plus what the experimenter provided outside of the training sessions.

Behavioral testing

Using all whiskers, mice were trained to discriminate between three commercial-grade sandpapers (P600, P800, and P1000) in a Go/No-go paradigm as described previously [66]. The control of devices and recording of behavioral parameters were performed with a data acquisition interface (PCI 6503, National Instruments, Austin, TX) and a custom-written LabWindows/CVI software (National Instruments, Austin, TX). Licks were detected electrically: mice were sitting on a metallic plate from which a current of 1.2 μA was applied and closed a circuit when their tongue touched the spout. Sandpapers were attached onto a four arms wheel (P800 × 2, P600, and P1000) mounted on a stepper motor (T-NM17A04, Zaber, Vancouver, Canada) and a motorized linear stage

(T-LSM100A, Zaber, Vancouver, Canada) to move textures in and out of reach of whiskers. For each trial, the wheel spun for a random time in a back position and stopped between 2 textures positions. Then, the wheel moved in a front position and the selected texture fell onto the whisker field at approximately 15 mm from the snout with an angle of 70° relative to the rostro-caudal axis. Initially, mice were trained to trigger a 4-6 μl sucrose water reward (100 mg/ml) by licking the spout during the presentation of the target stimulus (P800). Then, they were gradually introduced to the No-go stimuli (P600 and P1000) within two-three sessions (pre-training; one session *per* day of 150-300 trials each). The training session started when Go and No-go stimuli were pseudo-randomly presented with 50% probability (P600 and P1000 were presented with a probability of 25% each) for each trial type with a maximum of 4 consecutive presentations of the same stimuli. A trial consisted in a 1 s pre-stimulus period followed by a 3 kHz auditory cue for 200 ms, a delay period of 500 ms after which the texture reached the whiskers within 150 ms and remained for 2 s before being retracted. Licking during the target stimulus presentation triggered a water reward at the end of the 2 s of presentation and was scored as a ‘Hit’. Licking during the non-target stimulus presentation triggered a 500 ms white noise at the end of the 2 s of presentation plus a 5 s time out period and it was scored as a ‘false alarm’ (FA). In the absence of lick during stimulus presentation, trials were scored as ‘miss’ or ‘correct rejection’ (CR) for target and non-target stimuli, respectively. To refrain from compulsive licking during training, in addition to the aforementioned rules, mice had to show a 2 times increase in the licking rate during stimulus presentation compared to baseline to get rewarded on target stimulus presentation. 250-400 trials *per* session were performed (1 session *per* day) at a rate of 6 trials/min.

We delivered light for optogenetic inhibition of layer V by shining light at wavelength $\lambda = 595$ nm through an LED (M595L3, Thorlabs, Newton, NJ; light power density, ~48 mW/mm^2) from the beginning of the training sessions on the cranial window in 20% of trials (‘Light WIN’ in Figure 4). Light was delivered 100 ms before the texture started moving into the mouse whisker field and it was turned off 100 ms after the 2 s texture presentation using a linear ramp function for 500 ms to avoid rebound activity [67, 68]. In another 20% of trials, light (wavelength, $\lambda = 595$ nm; light power, 2 mW) from a LED placed in front of the mouse eyes was delivered the same way as for the ‘light WIN’ trials (with the same ramp for extinction) in order to control for the putative effect of direct retinal light stimulation during ‘light WIN’ trials on mouse performance (‘Light EXT’ in Figure 4). In the remaining 60% of trials no light was delivered (‘Light OFF’ in Figure 4). For experiments in Figure 4D, under light anesthesia (4% isoflurane at 0.4 l/min), a small hole was drilled through the cranial window to insert a glass pipette through which 300 nL of muscimol (Bodipy-TMR-X, 5 mM in saline buffer with 5% DMSO) was injected at 300 μm and 500 μm below the pia. Mice were left to recover for 45 min and their behavioral performance was then assessed for another 100 trials.

Histology

The animals were deeply anesthetized with urethane and transcardially perfused with 0.01 M PBS, pH 7.4, and then 4% paraformaldehyde (PFA) in phosphate buffer (PB; pH 7.4). The brains were post-fixed overnight (ON) at 4°C and subsequently cut to obtain coronal slices of 40 μm thickness. Sections were incubated ON, or for 48 h, at 4°C in primary antibody diluted in a PBS solution containing 5% NGS, 0.3% Triton X-100, and 0.015% sodium azide or in PB containing 0.3% Triton X-100 and 10% BSA. Sections were then incubated for 2–3 h at RT in the appropriate secondary antibody. The sections were counterstained by incubation with Hoechst (1: 400) for 20 min at RT, mounted on glass slides using Mowiol (Sigma Aldrich, Saint Louis, MO) and coverslipped. To distinguish the boundary between layer V_a and V_b, sections were incubated at 37°C for 1 to 4 hours in a PBS solution containing 0.03% Cytochrome-c, 0.01% Catalase, 0.05% 3,3′-diaminobenzidine (Sigma Aldrich, Saint Louis, MO). Primary antibodies used: Anti-GABA (1:1000 rabbit, Sigma A2052); Anti-Somatostatin (1:100 rat, Millipore MAB354, Billerica, MA); Anti-Parvalbumin (1:500 mouse, Sigma P3088); Anti-NeuN (1:250 mouse, Millipore MAB377, Billerica, MA). Alexa-conjugated (Invitrogen, Carlsbad, CA) secondary antibodies were used.

Fluorescence images were acquired with a Leica SP5 inverted confocal microscope. Cells were counted within a given cortical layer in at least 3 sections *per* animal and in at least 3 different animals. The sections were randomly sampled in the rough volume (1.5 mm radius) around the injection site. z stacks of images were acquired at steps of 2 μm (total z distance, 10 μm). Boundaries between layers were defined using Hoechst staining (Figures S1B and S1C) or DIC images (Figures S1D and S1E). For morphological reconstruction of recorded neurons, at the end of the *in vitro* experiment cortical slices were fixed in 4% PFA in PB at 4°C ON. Afterward, the tissue was transferred to a fixative-free PBS solution. For *in vivo* experiments, coronal slices (200 μm thick) obtained from the fixed brain were transferred to a fixative-free PBS solution. To reveal biocytin-filled neurons, after PBS rinsing, quenching of endogenous peroxidase and permeabilization, slices were treated with avidin-biotinylated horseradish peroxidase complex (Vectastain ABC elite, Vector Laboratories, Burlingame, CA) and then labeled with a 3,3′-diaminobenzidine solution (DAB Peroxidase Substrate Kit, Vector Laboratories, Burlingame, CA). After incubation with Hoechst (1: 400, 20 min, RT), slices were mounted on glass slides and coverslipped. Morphological reconstructions were performed using Neurolucida (MBF Bioscience, Williston, VT).

QUANTIFICATION AND STATISTICAL ANALYSIS

Statistical methods

Values are expressed as mean ± s.e.m. Statistical analysis was performed with MATLAB software (Mathworks, Natick, MA) and GraphPad Prism 5 software (GraphPad Software, San Diego, CA). To test for normality of data distribution a Kolmogorov-Smirnov test was run on each experimental sample. The significance threshold was always set at 0.05. When comparing two populations of data, t test was used to calculate statistical significance in case of normal distribution, otherwise the non-parametric Mann-Whitney or Wilcoxon signed-rank (for unpaired and paired comparison, respectively) tests were used. When multiple populations of data were

compared, one-way ANOVA with Bonferroni post hoc test was used in case of normal distribution, otherwise the non-parametric Friedman or Kruskall-Wallis with Dunn post hoc test were used for paired and unpaired comparison, respectively. All tests were two-sided, unless otherwise stated. For statistical comparison of the 100 repetitions shown in Figure 3F a Binomial test at the $p = 0.05$ level was used (see Juxtasomal Recordings in Awake Mice for details). For statistical comparison of behavioral data shown in Figure 4, a one-tailed Paired t test was used with post hoc Holm-Bonferroni correction for multiple comparison. The number of samples (n) and p values are reported in the figure legends; n typically refers to the number of neurons, unless otherwise stated. In all figures: *, $p < 0.05$; **, $p < 0.01$; ***, $p < 0.001$ and error bars indicate s.e.m. No statistical methods were used to pre-determine sample size, but we collected sample sizes similar to those reported in previous publications [6, 35]. Blinding was not used in this study. Criteria for data inclusion are described in following sections.

Analysis of *in vivo* recordings

In vivo *whole-cell patch-clamp experiments*

Neurons were defined as ChR2-positive or ChR2-negative cells according to the latency of their response to laser stimulation (< 1 ms for ChR2-positive cells; > 1 ms for ChR2-negative cells). Cells with average resting membrane potential more depolarized than −50 mV were excluded from analysis. Responses to blue light stimulation were separated in two classes on the basis of the pre-stimulus membrane potential of the recorded neuron. The trials defined as ‘resting state’ were the ones in which the pre-stimulus membrane potential (i.e., the mean membrane potential in the 20 ms prior the laser pulse) was within 5 mV of the most hyperpolarized value observed in that same neuron. The trials defined as ‘activated state’ included all trials in which the pre-stimulus membrane potential was more than 13 mV depolarized than the most hyperpolarized value observed in the same recorded cell, similarly to [69]. The integral of the membrane potential changes (named ‘Area’), the amplitudes of the maximal depolarization (‘Positive peak’), and the amplitude of the maximal hyperpolarization (‘Negative peak’) with respect to the pre-stimulus membrane potential were calculated on the average of all the trials in either ‘resting state’ or ‘activated state’, over a time window of 200 ms from the onset of the laser stimulus. For experiments with whisker stimulation, the ‘Area’, the maximal amplitudes of the whisker-evoked depolarization (‘Peak amp’), and the membrane potential value at the time of maximal whisker-response (‘V_m at whisker peak time’) were measured in a time window of 150 ms starting from the rising phase of the whisker-evoked response selecting trials in the resting state. The membrane potential of the cell before stimulation was calculated in a 5 ms time window before the rising phase of the whisker-evoked response. For the analysis of recordings reported in Figures 7A–7E, S6A–S6E, and S7D–S7I trials occurring in the activated membrane state were selected. In the experiments displayed in Figures 7E, S6E, S7F, and S7I ‘response probability’ quantified the efficiency of the blue laser stimulation to trigger a prolonged hyperpolarization when the light stimulus occurred during an ‘activated state’. ‘Response probability’ was calculated as the ratio between the number of single trials in which blue light hyperpolarized the membrane potential more than 4.5 mV with respect to the pre-stimulus membrane potential in a 200 ms time window post-stimulus and the total number of trials considered.

Juxtasomal recordings in anesthetized mice

In juxtasomal electrophysiological recordings spikes were identified with a threshold set at > 3 times the peak-to-peak noise value using Clampfit (Molecular Device, Sunnyvale, CA). Visual inspection was subsequently used to avoid false positives. Identified spikes were analyzed using customized MATLAB codes (Mathworks, Natick, MA). Only cells with > 50 trials in all protocols were considered for analysis. Neurons were considered positive for the excitatory opsin (ChR2) if they discharged AP during the 10 ms blue light illumination in more than 90% of the trials. Neurons were considered positive for the inhibitory opsin (Halo) if their firing (either spontaneous or sensory-evoked) was completely silenced during 500 ms yellow light illumination in more than 90% of the trials. We observed 20 halorhodopsin-positive neurons out of 90 juxtasomally recorded cells (22%). In 11 of the 20 halorhodopsin-positive neurons, whisker stimulation and light stimulation were performed at the same time and these cells are shown in Figures S4A and S4A$_1$. In the remaining 9 Halo-positive cells, whisker stimulation was performed either after light illumination or not at all. Opsin-positive neurons were excluded from the analysis displayed in Figures 1, 2, and S4C–S4J. Neurons were considered to respond to the whisker stimulation if there was a significant increase (t test or Mann-Whitney, $p = 0.05$ one-tailed) in their spike activities during the post-stimulus window (ranging from 0 to 100 ms after the onset of the stimulus presentation) as compared to the activity in the pre-stimulus time window (ranging from −100 to 0 ms with respect to the onset of whisker deflection). Since the temporal profile of the spike responses was highly variable and to avoid missing any responsive neurons, we counted the number of spikes in 1 ms bin in both the pre- and the post-stimulus window. Using sliding windows of increasing duration (window duration: 10-100 ms, duration increase: 1 ms) we compared, for each sliding window duration, the intervals with the maximal number of spikes in the pre-stimulus window with the intervals with the maximal number of spikes in the post-stimulus window. A neuron was considered responsive when a significant increase between the pre-stimulus and post-stimulus activity in at least one sliding window duration was observed. False positive and false negative were corrected by visual inspection. Among all the opsin-negative neurons, 13 out of 27 cells and 19 out of 70 neurons were considered whisker-responsive for the dataset shown in Figures 1 and 2, respectively. Peri-stimulus time histograms in Figures 1E–1E$_3$, 1I, 1I$_1$, and S7A–S7C were obtained by binning each individual response with 10 ms bins, averaging across trials and then across neurons. Error bars indicate the standard error of the mean across neurons. For Figures 1F, 1J, S4D, and S4H mean values of spikes rates were computed averaging individual spikes across trials and then across neurons during the response window (0-100 ms). To compute the accuracy of whisker stimulation time estimation that can be estimated by observing neural population activity (Figures 2G, 2H, and S4C–S4J), we defined population response events (PREs) from single-trial pseudo-simultaneous population responses obtained by pooling together many neurons recorded sequentially with the juxtasomal

techniques. More precisely, in each trial we identified a PRE at any given time around the whisker stimulation when the number of spikes *per* neuron in a 10 ms window centered at that time was larger than a certain threshold (*Th*). The threshold *Th* was an arbitrary parameter, which we kept the same for each analyzed population across all conditions of whisker and laser stimulation. The values of *Th* chosen for the populations of cells displayed in Figures 2 and S4C–S4J were set at 0.1 spikes *per* neuron and 0.04 spikes *per* neuron respectively, as these parameters empirically produced higher occurrences of PREs straight after whisker stimulation and lower percentages of PREs before whisker stimulation. However, we verified that changes in the stimulus time estimation errors across different laser conditions were very similar across variations of the *Th* parameter. The quantification of the accuracy of the whisker stimulation time estimation in each trial (absolute error) was obtained from the PREs by measuring their time (PRE time) in each trial in a time window from −40 ms to +100 ms with respect to the whisker-stimulation onset and then subtracting from each PRE time the value of the mode of the PRE distribution for each condition and by taking the absolute value ('absolute error' in Figures 2G, 2H, S4F, and S4J). This was because the value of this mode was taken as the typical latency between whisker stimulation and PRE response.

Our calculations of PRE reported in Figures 2 and S4C–S4J do not take into account the effects of noise correlations, because they are based on a pseudo-simultaneous population responses constructed by collecting together responses of non-simultaneously recorded neurons. Noise correlations in barrel cortex are mainly positive [70], and positive noise correlations tend to amplify the variability of the time course of the population activity [71]. By ignoring such correlations our method may eliminate any strong and narrow spontaneous activity peaks in the pooled population – in effect, pseudo-population responses may reduce "false positives" in PRE compared to real populations that contain correlated noise. To test for this, we used the procedure of [72] to generate correlated spike trains that matched exactly the true population trial-averaged post-whisker-stimulation time-dependent firing rate of the neurons analyzed here (sampled with 10 ms time resolution) under both the control, the blue light and the yellow light condition, and on top of that could have arbitrary values of noise correlations. We then varied the noise correlations of the simulated data between a value of 0 (corresponding to the pseudo-simultaneous responses that would be obtained by pooling data from neurons recorded non-simultaneously) and a value 40 times larger than those of real noise correlation of nearby whisker-response neurons, as measured from a previously published dataset of 52 neuronal pairs simultaneously recorded with a small array of extracellular electrodes in the D2 barrel related column from urethane anaesthetized rats in response to whisker deflection [70]. We found (results not shown) that the distribution of PRE times simulated with realistic noise correlations was shorter (One-way repeated-measures ANOVA, $p < 1E-3$) with blue light stimulation than with respect to control, and longer (Paired t test, $p < 1E-3$) with yellow light stimulation than with respect to control, in excellent agreement with the real-data pseudo-population results (Figures 2G and 2H). Furthermore, these differences were still significant (Friedman test, $p < 1E-3$ for blue light stimulation; Paired t test, $p < 1E-13$for yellow light stimulation) when increasing the noise correlations up to 40 times the ones previously reported by Petersen and colleagues [70]. As explained in [71] the effect of increasing noise correlations on such temporally varying firing rates is to create more frequent and stronger spontaneous random population activity peaks due to noise amplification by correlations. Layer V activation canceled out effectively these PRE events due to noise amplification for all tested levels of noise correlations, thereby making whisker deflection estimation more robust even in the presence of correlated neuron-to-neuron variations. Our interpretation of these results is that the increase of accuracy in coding whisker deflection time brought about by the firing of layer V pyramidal neurons would be maintained in the presence of noise correlations among whisker-responsive cells. Experiments in which the multiunit activity was recorded simultaneously using 16 channels silicon linear probes confirmed this interpretation (Figures S4K–S4R).

Whisker imaging and tracking

For data shown in Figure 3, a high-speed rate camera (Basler acA800, Ahrensburg, DE) was used and whiskers were imaged from below through an objective lens (f = 8 mm) and a mirror angled at 45 degrees placed below the running wheel. Whiskers were lit from above using custom diffused infrared LEDs. High-speed videos were acquired at 1 kHz with a 200-μs exposure time and were synchronized with electrophysiology data via external triggers. Whiskers touches onto the pole in both protraction and retraction phase were manually tracked from the videos and time points of each individual whisker contact with the pole were identified using a custom MATLAB script. Touch duration and inter-touch intervals were extrapolated. Whisker contacts with the pole were considered touches if the whisker touched the border of the stimulus bar for at least 8 frames. Whisker tracking was also performed offline using Whisk (https://www.janelia.org/open-science/whisk-whisker-tracking; [55]), which returned whisker position for every frame. Data from this analysis were processed using custom MATLAB codes to obtain whisker amplitude (half-width of envelope) and set-point (median angle of envelope) in all the frames recorded (independently on the presence of whisker touches).

To compare touches kinematics under control conditions and during optogenetic inhibition of layer V neurons, we computed the duration of touches and the inter-touch intervals (start-to-start). For each cell which responded with an increase in AP firing rate upon pole touch (touch-responsive neurons, see next section) the median of touch duration and inter-touch interval across touches was calculated and these values were averaged across cells. The mean values indicated that yellow light stimulation did not affect touch kinematics (touch duration: 28 ± 2 ms versus 27 ± 2 ms under control conditions and during optogenetic inhibition of layer V, Paired t test p = 0.62; inter-touch interval: 71 ± 3 ms versus 69 ± 2 under control conditions and during optogenetic inhibition of layer V, Paired t test p = 0.43, n = 12 cells from 8 animals). For each neuron the median of whisker amplitudes and whisker set-points, relative to the rostro-caudal axis, across frames was calculated and then averaged across cells (whisker amplitude: 10 ± 3 deg versus 13 ± 3 ms deg under control conditions and during optogenetic inhibition of layer V, Paired t test p = 0.01; whisker set-point: 109 ± 6 deg versus 112 ± 6 deg under control conditions and during optogenetic inhibition of layer V, Paired t test p = 0.006, n = 12 cells from 8 animals).

Juxtasomal recordings in awake mice

Spikes identification and classification of opsin-positive and opsin-negative cells were performed as for recordings in anesthetized animals. We observed 16 halorhodopsin-positive neurons out of 64 juxtasomally recorded cells (25%). Out of the 48 opsin-negative cells that were recorded, only 23 cells had > 53 pole touches in both the tactile stimulation protocol and the tactile stimulation combined with optogenetic stimulation. These 23 cells were included in the analysis described below. We generated peri-touch raster plots and time histograms considering the touches and spikes in a time window of 500 ms under control conditions and during optogenetic inhibition of layer V. This time window was placed between 1 s and 1.5 s from the pole presentation for the optogenetic inhibition of layer V and between 0.5 s and 1 s for the control condition. The time windows [0 s - 0.5 s] and [1.5 s - 2 s] were not considered in the analysis to avoid electric artifacts due to the ascending and descending pole movement and to discard possible rebound effect in the spike activity after yellow light illumination. The times corresponding to whisker touches onto the pole were manually identified from whisker videos as described in Whisker Imaging and Tracking. To study the touch-evoked activity, we aligned the neuron spike times with respect to the touches onset (0 ms) and we plotted the corresponding raster plot in a peri-touch time window ranging from −20 to 40 ms from touch time. This time window was chosen such that: *i)* it was long enough to include the whole touch duration (mean touch duration across cells: 27.1 ± 1.2 ms under control conditions and 28.1 ± 1.5 ms during optogenetic inhibition of layer V, n = 23 cells from 12 animals); *ii)* it did not exceed the value of the mean inter-touch interval (mean inter-touch interval across cells: 70.2 ± 1.8 ms under control conditions and 70.9 ± 1.9 ms during optogenetic inhibition of layer V, n = 23 neurons from 12 animals). Successive touches containing overlapping spikes in this peri-touch windows as well as touches with peri-touch window at the border between the control condition and the optogenetic inhibition of layer V were removed from analysis.

To classify cells as ‘touch-responsive’ we used the same method described to define ‘whisker-responsive’ cells in recordings from anesthetized mice with the exception that: *i)* the time windows [-20 ms – 0 ms] and [0 ms - 40 ms] were used as pre-touch (Pre) and post-touch (Post) condition, respectively; *ii)* the size of the sliding time windows ranged from 5 ms to 20 ms (increasing size by steps of 1 ms). Twelve out of 23 recorded neurons were classified as ‘touch-responsive cells’ and used for the analysis displayed in Figure 3. Peri-touch time histograms in Figures 3C and 3C$_1$ were obtained by binning each cellular response (bin size:1 ms), convolving it with a Gaussian Kernel of standard deviation equal to 2 ms, and averaging across all touches and then across cells. Error bars indicate the standard error of the mean across neurons. For Figure 3D, mean values of spikes rates were computed averaging individual spikes across touches and then across neurons during the Pre and the Post time windows. The PRE analysis shown in Figures 3E and 3F was performed similarly to that performed on recordings in anesthetized mice. To create the single touch pseudo-simultaneous population response event, for each touch the spikes of the 12 touch-responsive neurons were pooled in the time window from ranging from −20 to +40 ms (bin size: 4 ms). This was done by randomly choosing for each cell a common minimum number of touches (53 touches without replacement) under both control conditions and during optogenetic inhibition of layer V and the *Th* to define a PRE was set to 0.1 spikes *per* neuron. Since the number of touches was different across cells, we performed 100 random repetitions of the previous operation and we calculated the ‘Absolute error’ under control conditions and during optogenetic inhibition of layer V neurons. These values were statistically compared for each repetition (Mann-Whitney test). Then, we used a Binomial test (p = 0.05) to evaluate over 100 repetitions if a significant increase or decrease in the absolute error during layer V optogenetic inhibition could be observed [73]. A significant p value was obtained only in the first case. We also controlled for false discovery rate (FDR) using two different procedures [74, 75] and the results were similar to the binomial test described above.

Linear probe recordings in anesthetized mice

Multi-unit activity was analyzed using Wave_Clus software [56]. Signals were filtered (800-8000Hz) and spikes events were detected with a threshold criterion set at 4 SD of the high-frequency signal. To reject spurious events, we used an unsupervised clustering algorithm based on superparamagnetic clustering on the feature extraction of the coefficients provided by the decomposition of the detected spikes by means of wavelet basis function [56]. Visual inspection was performed on the rejected spikes to confirm the reliability of the artifact rejection procedure.

The laminar electrodes location was determined with iCSD analysis of the local field potential (LFP) following the procedure described in [76]. CSDs were calculated from the trial-averaged LFP (frequency range 0.1–250 Hz; see [77] for details of the filtering procedure) measured at each electrode. The appearance of an early sink (8-10 ms after the onset of the whisker stimulation) was used to identify layer IV. This was followed by a propagation of sink activity in layer II/III and a source/sink in layer Va and Vb, respectively [10, 78]. The laminar electrodes location was finally assigned using layers boundaries as in [63].

The PRE analysis on laminar multiunit signals recorded with silicon probes was performed as described for juxtasomal recordings in anesthetized mice. In each animal, single-trial pseudo-simultaneous PRE were computed pooling together all the spikes from different channels in a time window ranging from −40 to +100 ms with respect to the onset of the whisker deflection (bin: 10 ms; *Th* > 0.05 spikes *per* channel). The accuracy of stimulus time estimation (‘Absolute error’) was calculated as previously described for anesthetized mice. To study possible different contribution originated from individual cortical layers, we performed the analysis by considering recordings from electrodes positioned in granular and infragranular layers.

Analysis of *in vitro* experiments

Cells were classified as opsin-positive or opsin-negative according to the latency of their response to laser stimulation (< 1 ms for ChR2-positive cells). Cells with average resting potential more depolarized than −55 mV were excluded from the analysis. In all the experiments cell input resistance (R_{in}) was calculated from hyperpolarizing current injections. AP threshold, amplitude, duration, and after-hyperpolarization (AHP) were calculated at rheobase, where rheobase was defined as the lowest current pulse value

needed to elicit an AP. Frequency adaptation was calculated as the ratio between the first (ISI_1) and last interspike (ISI_n) interval during a 400 ms current injection at 2 x rheobase (Table S5). For experiments in Figures 1G–1J, functionality of the opsin was confirmed by recording from Halo-positive neurons in cortical slices. Yellow light illumination (stimulus duration, 500 ms) suppressed the firing activity of Halo-positive neurons in cortical slices (firing rate: pre, 9.4 ± 1.7 Hz; stim, 0.0 ± 0.0 Hz; post, 9.7 ± 1.1 Hz, n = 6 cells, Friedman test, p = 1E-4). For experiments in Figure 6, interneurons were distinguished from regular spiking pyramidal cells on the basis of their electrophysiological properties (lower AP half-width and higher, usually non regular, firing frequency, Table S5) [39]. In some experiments, this was further confirmed based on post hoc anatomical reconstruction. Interneurons were divided in fast spiking (FS) and non-fast spiking (NFS) cells on the basis of their R_{in}, rheobase, AP half-width and AHP (Table S5) [79]. Higher non-adapting firing frequency and the absence of sag were considered typical of FS interneurons [39]. Blue light-evoked membrane depolarizations were analyzed averaging different trials and measuring the 'positive peak amplitude', the 'negative peak amplitude', and the 'area' underneath the membrane potential response over a period of 500 ms from the light onset (Figures 5 and 6C–6C_2). In voltage-clamp experiments reported in Figures 6D–6F, trials were averaged and the excitatory and inhibitory charges were quantified integrating the inward and outward currents from the onset of light pulse and the time point where the currents returned to zero. Experiments in Figures 7H–7K and S6H–S6K were performed on either ChR2-negative or ChR2-positive neurons. IPSCs measured at +10 mV in ChR2-positive neurons were completely abolished by the application of gabazine, confirming the pure GABAergic nature of these currents. In experiments displayed in Figures 7K and S6K, the charge transferred was calculated in a time window of 255 ms starting from the onset of the blue laser. In Figures S5E and S5J the effect of opsin-induced inhibition was quantified calculating the firing frequency before, during, and after yellow light illumination. In each cell, this was done at the value of depolarizing current injection that, in the absence of optogenetic manipulation, induced continuous AP discharge over the entire duration of the current injection. In Figures 7F, 7G, S6F, and S6G, the peak amplitude of the yellow and the blue light-evoked responses were measured at the peak with reference to the membrane potential values (in a 5 ms time window) immediately before the stimulus.

Analysis of behavioral experiments

The overall performance of the animal was calculated as the percentage of correct trials (Hit + CR) over the total number of trials *per* session under the different experimental conditions. Only sessions in which the performance was $\geq$ 75% in the absence of light stimulation (Light OFF) were considered for the evaluation of the optogenetics effects (n = 15 sessions from 5 animals). Performance average was done across sessions and error bars indicated the standard error of the mean. In each Hit and FA trial, the RT was considered as the time of occurrence of the first lick within the texture presentation window. RTs in FA trials tended to become longer when the yellow light was delivered onto the cranial window compared to when the light was off although the effect did not reach statistical significance (mean RT: 788 ± 84 ms versus 643 ± 71 ms in Light WIN and Light OFF conditions, respectively; one-tailed Paired t test, p = 0.09 Holm-Bonferroni corrected, n = 12 sessions from 5 animals). Statistical comparisons were done performing one-tailed t test and the obtained p values were corrected with the Holm-Bonferroni post hoc correction for multiple comparisons. For the analysis of RT times in Hit trials (Figure 4C), we tested if covariations across data points (sessions were performed on different animals with each animal having a different mean RT) could affect statistical comparisons. We normalized the RT of each session from a given mouse to the mean RT obtained for that animal in the Light OFF condition. This procedure regressed away the effect of covariations across sessions by differences in the mean RT of individual mice. After this normalization, we obtained p values (one-tailed Paired t test with Holm-Bonferroni correction for multiple comparison: p = 0.006 for Light OFF versus Light WIN; p = 0.048 for Light EXT versus Light WIN; p = 0.178 for Light OFF versus Light EXT) similar to those of not normalized data shown in Figure 4C.

DATA AND CODE AVAILABILITY

The dataset and codes supporting the current study have not been deposited in a public repository because of their size and non-standard format but they are available from the Lead Contact upon request.

Current Biology, Volume *30*

Supplemental Information

Temporal Sharpening of Sensory Responses

by Layer V in the Mouse

Primary Somatosensory Cortex

Dania Vecchia, Riccardo Beltramo, Fabio Vallone, Ronan Chéreau, Angelo Forli, Manuel Molano-Mazón, Tanika Bawa, Noemi Binini, Claudio Moretti, Anthony Holtmaat, Stefano Panzeri, and Tommaso Fellin

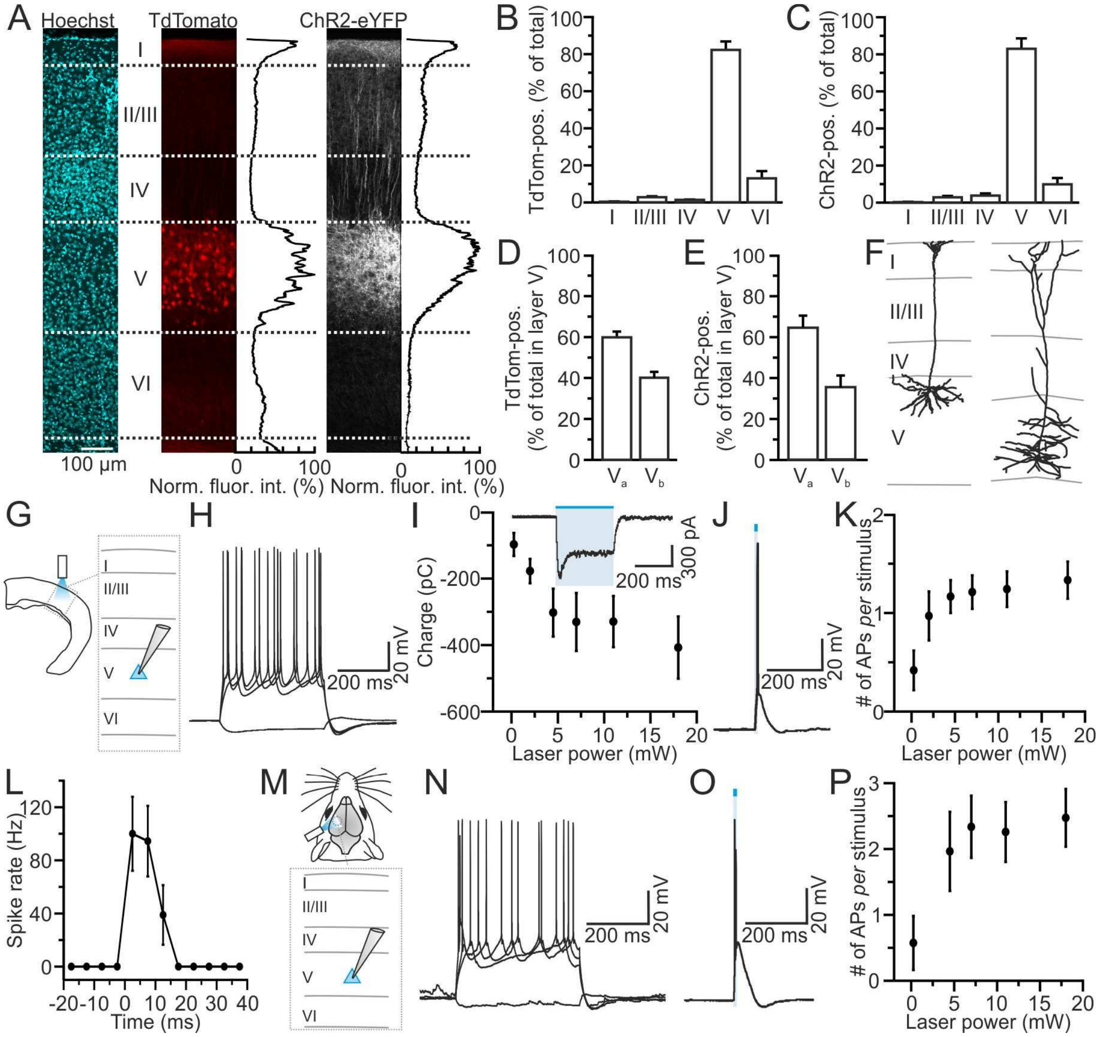

Figure S1. Selective expression of transgenes and functional characterization of ChR2 in layer V principal cells. Related to Figure 1. (A) Confocal images showing a coronal section of the primary somatosensory cortex of a double transgenic mouse *Rbp4-cre* x *TdTomato* which was injected with AAV transducing ChR2-eYFP. Left: The boundaries of cortical layers (dashed lines) were identified on the basis of cellular density using Hoechst staining. Middle and right: confocal images showing TdTomato (middle) and ChR2-eYFP (right) expression. The normalized fluorescence intensity is shown on the side for both images. **(B-C)** Percentage of TdTomato- (B) and ChR2-expressing cells (C) across cortical layers. Data were pooled from 3 animals (3 sections *per* animal). Within layer V, we found that 22 ± 6% and 16 ± 3% of the total Neuronal Nuclei- (NeuN) positive population expressed TdTomato and ChR2, respectively (n = 3 animals, 3 sections *per* animal). **(D-E)** Percentage of TdTomato- (D) and ChR2- (E) expressing cells within layer V_a and layer V_b. **(F)** Examples of ChR2-positive layer V cells that were filled with biocytin and reconstructed morphologically *a posteriori*. A layer V_a slender-tufted pyramidal neuron is shown on the left and a layer V_b thick-tufted pyramidal cell on the right. **(G)** Schematic representation of the experimental configuration used for brain slice experiments. Blue light was delivered to isolated cortical slices through a fiber optic and recordings were performed from ChR2-positive neurons (blue triangle). **(H)** Representative current-clamp patch-clamp recordings showing the membrane potential responses to current injections (-100, +350, +500, +650 pA) of a ChR2-positive layer V neuron. **(I)** Average charge transfer as a function of the light intensity (stimulus duration: 300 ms; n = 7 ChR2-positive cells). Inset: representative voltage-clamp recording showing blue light-induced photocurrent. Photocurrent latency: 0.20 ± 0.03 ms, n = 7 cells. **(J)** Representative current-clamp patch-clamp recording from a ChR2-positive layer V neuron showing the membrane potential response to 10 ms of blue light stimulation. **(K)** Average number of action potentials (AP) *per* stimulus as a function of the light stimulus intensity in ChR2-positive layer V neurons (n = 12 cells). A 10-ms light pulse in ChR2-positive neurons elicited one or few APs on average, over a large range of power values. Since our goal was to probe the cortical responses to a brief activation of layer V pyramidal cells, we used a 10-ms photoactivation stimulus throughout this study. **(L)** Average peri-stimulus time histogram (bin: 5 ms) of layer V ChR2-positive neurons during blue light stimulation (stimulus duration: 10 ms; laser power: 4.6-5 mW), n = 12 cells. **(M)** Schematic of the experimental configuration for *in vivo* recordings. **(N)** Representative current-clamp patch-clamp recording from a ChR2-positive layer V neuron *in vivo* showing membrane

potential responses to current injections (-100, +350, +450, +550 pA). **(O-P)** Same as in (J-K) for ChR2-positive layer V pyramidal neurons *in vivo*. In (P), n = 5 cells from 4 animals. Latency to membrane depolarization: 0.31 ± 0.02 ms, n = 5 cells.

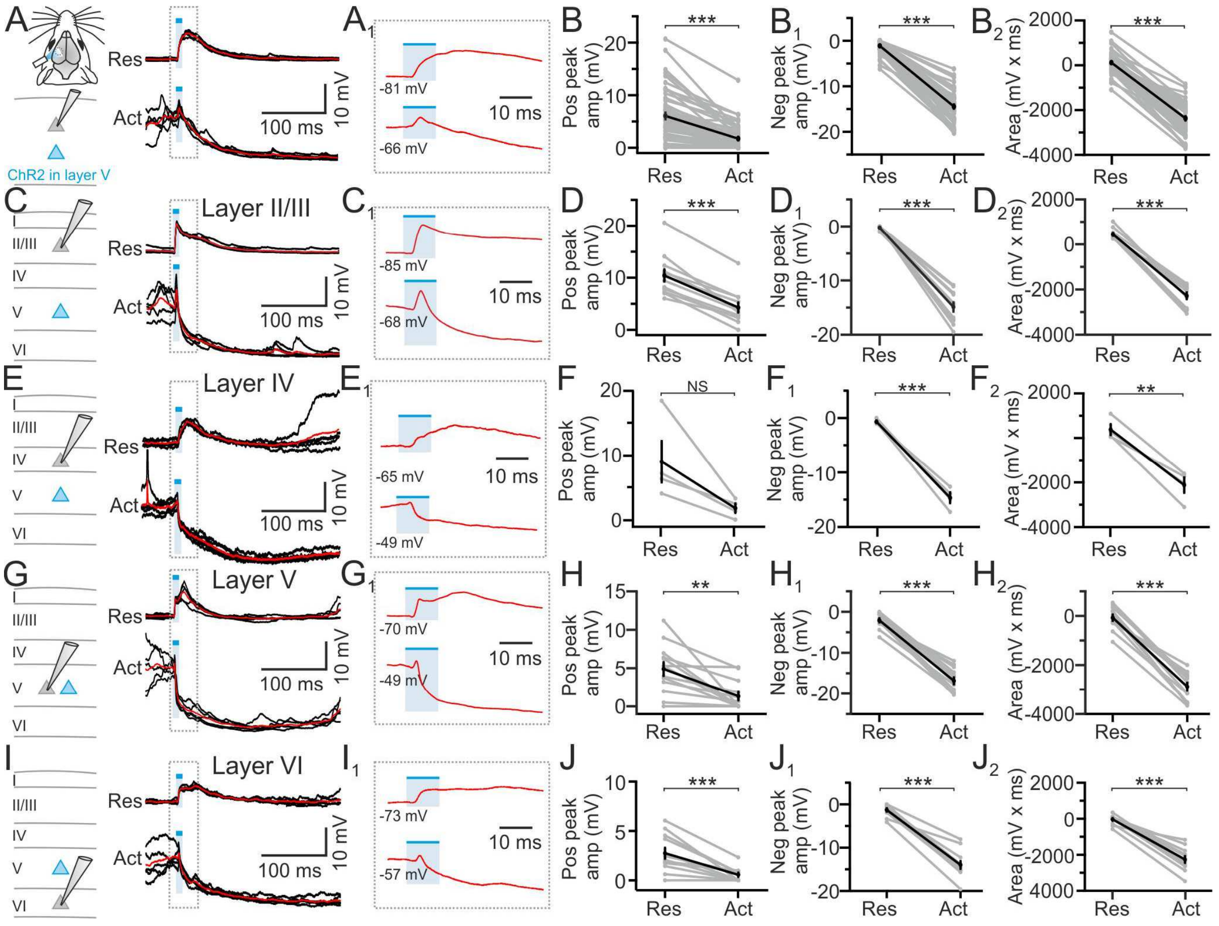

Figure S2. Layer V photostimulation evokes state-dependent changes in the membrane potential of cortical neurons *in vivo*. Related to Figure 1. (A) Left: Schematic of the experimental configuration for intracellular recordings in anesthetized mice. Blue light was delivered through a fiber optic. In this as well as in other figures ChR2-positive neurons are indicated in blue, ChR2-negative cells in grey. Right: Representative patch-clamp current-clamp recordings from a ChR2-negative pyramidal neuron showing cellular responses to blue light (blue bars, 10 ms duration) during spontaneous activity. Responses to five different light stimuli are shown in black and their average in red for light stimuli during the resting (Res, top) and the activated state (Act, bottom). **(A_1)** Zoom in of the average response (red) highlighted in (A, grey dotted line). **(B-B_2)** Positive peak amplitude (B), negative peak amplitude (B_1), and integral (Area, B_2) of the light-evoked response in ChR2-negative principal neurons in the resting (Res) and activated (Act) states. In this as well as in other figures, values from individual experiments are shown in grey, the average of all cells in black. n = 55 cells from 30 animals; Wilcoxon test in (B-B_1); Paired Student's *t*-test in (B_2). Subthreshold latency: 2.90 ± 0.09 (Res) and 2.37 ± 0.10 (Act). **(C-D_2)** Same as in (A-B_2) for ChR2-negative layer II/III principal neurons. n = 12 cells from 6 animals; Paired Student's *t*-test. **(E-F_2)** Same as in (A-B_2) for ChR2-negative layer IV principal neurons. n = 4 cells from 4 animals; Paired Student's *t*-test. **(G-H_2)** Same as in (A-B_2) for ChR2-negative layer V principal neurons. n = 13 cells from 8 animals; Paired Student's *t*-test. **(I-J_2)** Same as in (A-B_2) for ChR2-negative layer VI principal neurons. n = 12 cells from 9 animals. Subthreshold latencies for cells across layers are reported in Table S2.

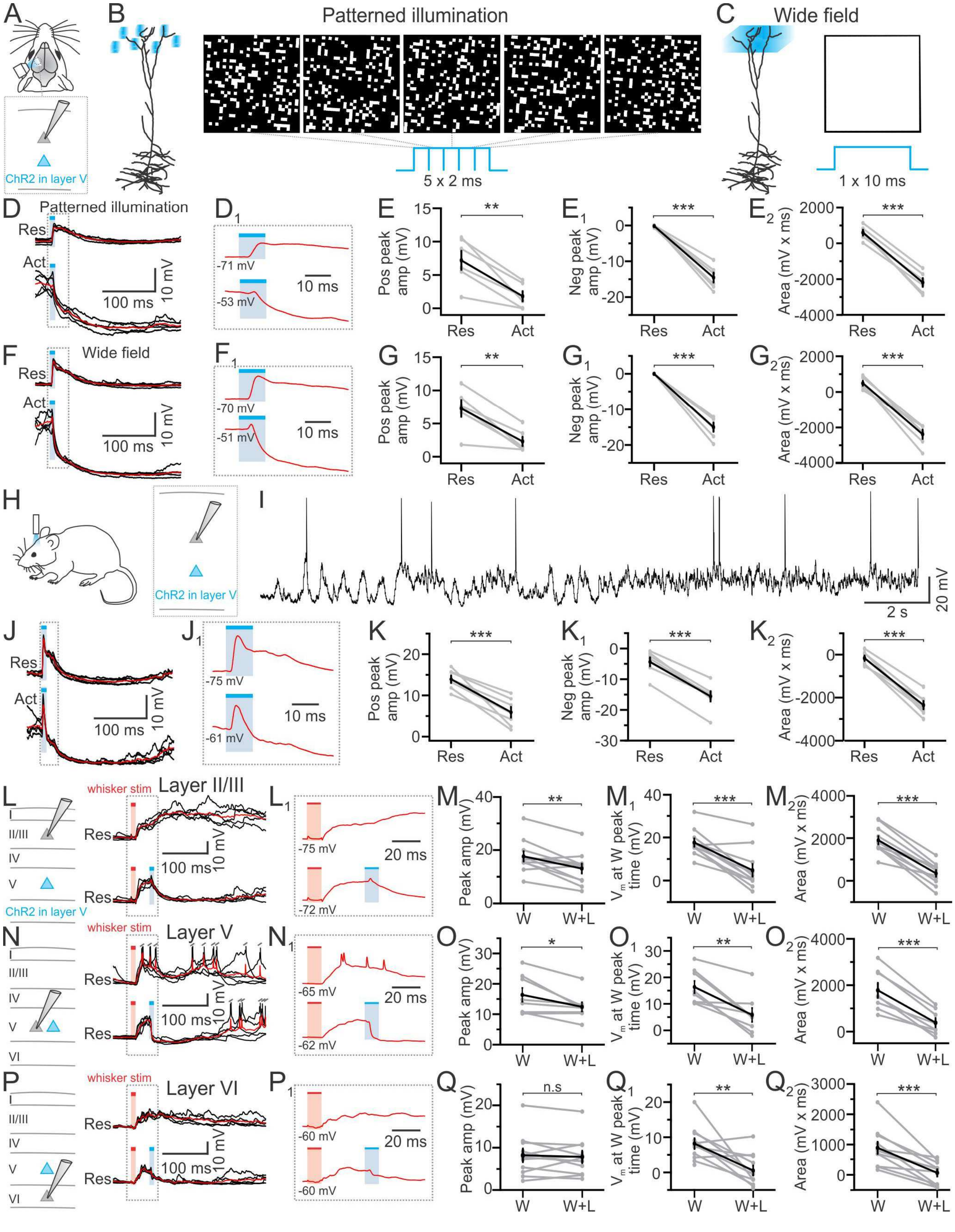

Figure S3. Stimulation of layer V pyramidal neurons either with patterned illumination or in awake mice generates state-dependent responses in cortical neurons. Related to Figure 1. (A) Schematic representation of the experimental configuration for patterned illumination in anesthetized animals. Fiber optic-mediated wide-field illumination may lead to non-physiological activation of ChR2-positive neurons. Because the fiber optic used for the photostimulation in Figures 1A-F and Figure S2 was placed on the pial surface, and because blue light is strongly scattered and absorbed within the brain, the neuronal structures that were illuminated most strongly under our experimental conditions were the distal

dendrites of layer V neurons. The photostimulation would thus likely cause synchronous depolarization of large portions of the dendritic trees. In contrast, under physiological conditions, cortical pyramidal neurons are bombarded by bursts of depolarizing synaptic inputs, each spatially confined. To mimic this spatially restricted pattern of dendritic activation *in vivo*, we used an optical illumination system based on a digital micromirror device (DMD; see STAR Methods). We projected light patterns of arbitrary geometry on the cortical surface, at a fast refresh rate (500 Hz). We selected a 400 x 400 μm^2 or 200 x 200 μm^2 field of view and we illuminated it with a spatially randomized patterns of 12-μm light spots (patterned stimulation, see STAR Methods for details). **(B)** The dendritic tree of layer V neurons was illuminated *in vivo* with complex light patterns (patterned illumination) to mimic physiological depolarization generated by local dendritic inputs. Five consecutive patterns of randomly generated spots (black and white images on the right) were projected for 2 ms each. White pixels indicate regions illuminated by blue light, black pixels regions that were not illuminated. **(C)** In each patterned illumination experiments, wide field illumination was also performed as control. **(D)** Representative current-clamp recordings from a ChR2-negative principal neuron *in vivo* during patterned optogenetic activation of layer V. Responses to five different trials are shown in black and their average in red for stimuli that occurred during the resting (Res, top) and activated state (Act, bottom). **(D_1)** The portion of the average response highlighted in (D, grey dotted line) is displayed at an enlarged scale. **(E-E_2)** Positive peak amplitude (E), negative peak amplitude (E_1), and area (E_2) of the light-evoked membrane response in ChR2-negative principal neurons in the resting (Res) and activated (Act) states following patterned illumination. n = 6 cells from 3 animals; Paired Student's *t*-test. **(F-G_2)** Same as in D-E_2 for wide field illumination. n = 6 cells from 3 animals; Paired Student's *t*-test. Wide-field illumination obtained with either the fiber optic (Figures S2A-B_2) or the DMD, which precisely controlled the area of illumination (400 x 400 μm^2 or 200 x 200 μm^2), produced similar responses on cortical neurons. Subthreshold latencies are reported in Table S2. **(H-I)** Schematic representation of the experimental configuration for intracellular recordings in awake mice (H) and example of continuous recording from a ChR2-negative neurons under these experimental conditions (I). **(J-J_1)** Representative current-clamp recording from a ChR2-negative principal neuron in an awake mouse. Five superimposed responses (black), and their average (red) are shown for wide field stimuli occurring in the resting (top) and activated state (bottom). **(K-K_2)** Same as in G-G_2 in awake mice. n = 7 cells from 3 animals; Paired Student's *t*-test. Subthreshold latencies are displayed in Table S2. **(L)** Left: schematic representation of the experimental configuration. A single whisker (C1, C2, or D1) was deflected with a piezoelectric actuator (stimulus duration: 10 ms) while patch-clamp current-clamp recordings were performed in the correspondent barrel related column in anesthetized animals (which was identified using intrinsic optical imaging, see STAR Methods for more details). Electrophysiological recordings were combined with optogenetic layer V activation (blue light duration: 10 ms). Right: representative traces from a layer II/III ChR2-negative neuron *in vivo*. Cellular responses to whisker deflection (red bar) and to whisker deflection followed by optogenetic activation of layer V pyramidal cells (blue bar) are shown. **(L_1)** The average responses highlighted in (L) are displayed at an enlarged scale. **(M-M_2)** Positive peak amplitude (M), membrane potential value at the whisker-response time peak (M_1) and area (M_2) in ChR2-negative principal layer II/III cells under the different experimental conditions. n = 11 cells from 9 animals; Paired Student's *t*-test. **(N-O_2)** Same as in (L-M_2) for identified ChR2-negative layer V principal neurons. n = 8 cells from 7 animals; Paired Student's *t*-test. **(P-Q_2)** Same as in (L-M_2) for identified ChR2-negative layer VI principal neurons. n = 11 cells from 9 animals; Paired Student's *t*-test.

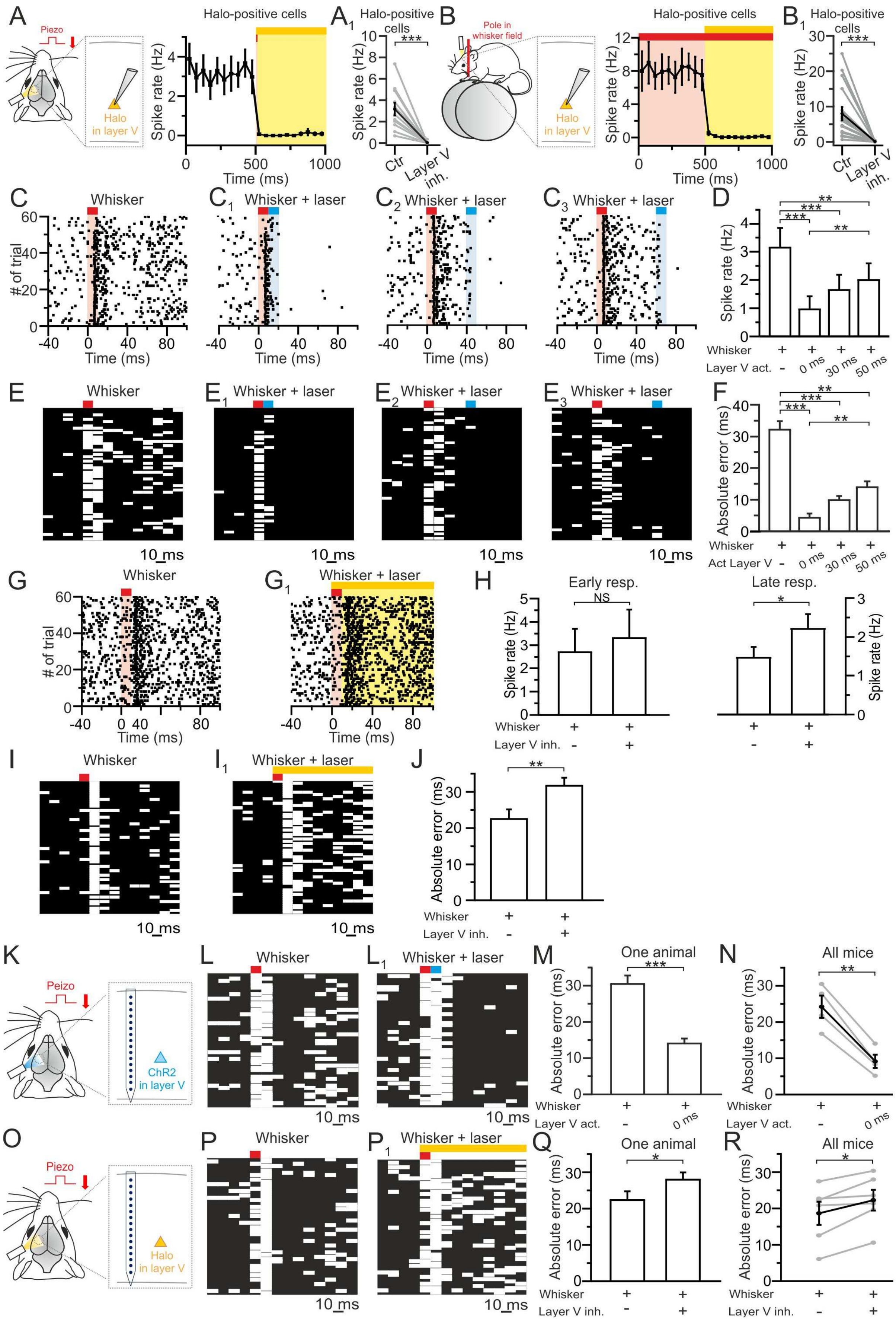

A
Piezo
Halo in layer V
Halo-positive cells
Spike rate (Hz)
Time (ms)
A1 Halo-positive cells
***
Ctr
Layer V inh.
B
Pole in whisker field
Halo in layer V
Halo-positive cells
B1 Halo-positive cells
C Whisker
# of trial
C1 Whisker + laser
C2 Whisker + laser
C3 Whisker + laser
D
Whisker
Layer V act.
0 ms
30 ms
50 ms
E Whisker
E1 Whisker + laser
E2 Whisker + laser
E3 Whisker + laser
10 ms
F
Absolute error (ms)
Act Layer V
G Whisker
G1 Whisker + laser
H
Early resp.
NS
Late resp.
Layer V inh.
I Whisker
I1 Whisker + laser
J
K
Peizo
ChR2 in layer V
L Whisker
L1 Whisker + laser
M One animal
N All mice
O
Piezo
Halo in layer V
P Whisker
P1 Whisker + laser
Q One animal
R All mice

Figure S4. Layer V sharpens the temporal profile of neuronal responses to whisker stimulation across cortical layers in individually recorded cells and in population recordings. Related to Figures 1-3. (A) Left: schematic representation of the experimental configuration in anesthetized mice. Recordings were performed from Halo-positive neurons. Optogenetic and whisker stimulations were performed as in Figure 1G. Right: peri-stimulus time histogram (bin: 50 ms) of Halo-positive cells under control conditions and after simultaneous whisker stimulation (red bar, stimulus duration: 10 ms) and layer V photoinhibition (yellow bar, stimulus duration: 500 ms). Juxtasomal recordings were performed as described in Figure S3L. n = 11 cells from 7 animals. Please note that 20 Halo-positive neurons out of 90 (22 %) were recorded in anesthetized animals (see STAR Methods). The plot in (A) shows the 11 cells in which whisker stimulation and light stimulation were performed at the same time. In the remaining 9 Halo-positive cells, whisker stimulation was performed either after light illumination or not at all. **(A_1)** Average spike rate under the different experimental conditions (Control, Ctrl, *vs* layer V inhibition, layer V inh.). n = 11 cells from 7 animals; Wilcoxon test. **(B)** Left: same as in (A) for experiments in awake mice actively whisking on a pole, which was lowered in the contralateral mouse whisker field. Optogenetic and whisker stimulations were performed as in Figure 3. Right: peri-stimulus time histogram (bin 50 ms) of Halo-positive cells during pole presentation (red bar) and simultaneous pole presentation and layer V photoinhibition (yellow bar, stimulus duration 500 ms). n = 16 cells from 11 animals. 16 Halo-positive neurons out of 64 (25 %) were recorded in awake animals (see STAR Methods). **(B_1)** Same as in (A_1) for awake mice. n = 16 cells from 11 animals; Paired Student's *t*-test. **(C-C_3)** Pseudo-simultaneous population response to whisker stimulation obtained pooling together whisker responsive and whisker nonresponsive sequentially-recorded deep neurons that were negative for ChR2. Juxtasomal recordings were performed as described in Figure S3L. Neural activity was recorded in response to vibrissae stimulation (red bar), in the absence (C) or presence of layer V photostimulation (blue bar, C_1-C_3). Blue light was presented at different delays (~ 0 ms in C_1, ~ 30 ms in C_2, ~ 50 ms in C_3) with respect to the time of onset of the whisker-evoked response. n = 27 cells from 13 animals. **(D)** Average cellular spike rate under the different experimental conditions. n = 27 cells from 13 animals; Friedman test. **(E-E_3)** PREs from all whisker responsive and whisker non-responsive cortical neurons (and all trials) during whisker stimulation (red bar, E) and whisker stimulation followed by layer V optogenetic activation (blue bar, E_1-E_3). **(F)** Average absolute error of PRE under the different experimental conditions. All PREs in the time window [-40 , +100] ms from the onset of the whisker stimulus were considered. n = 142 (no layer V activation), n = 42 (layer V activation at ~ 0 ms), n = 72 (layer V activation at ~ 30 ms) and n = 78 (layer V activation at ~ 50 ms) events for n = 27 neurons; Kruskal-Wallis test. **(G-G_1)** Pseudo-simultaneous population response to vibrissae stimulation (red bar) obtained pooling together whisker responsive and whisker non-responsive sequentially-recorded deep neurons (Halo-negative), in the absence (G) or presence of layer V optogenetic inactivation (yellow bar, G_1). Juxtasomal recordings were performed as in (C). **(H)** Same as in (D) for layer V photoinhibition during the early [0, 40] ms and late [40, 100] ms response time window. n = 70 neurons from 19 animals; Wilcoxon test. **(I-I_1)** PREs from all recorded cortical neurons (and all trials) during whisker stimulation (red bar, I) and whisker stimulation paired with layer V optogenetic inactivation (yellow bar, I_1). **(J)** Same as in (F) for layer V optogenetic suppression experiments. n = 177 (no layer V inactivation) and n = 183 (layer V inactivation) events for n = 70 neurons; Mann-Whitney test. **(K)** Schematic representation of the experimental configuration. Optogenetic and whisker stimulations were performed as in Figure 1. A 16-channels linear probe placed across cortical layers was used for recordings. **(L-L_1)** PREs from all simultaneous units and trials recorded in one animal during whisker stimulation (red bar, L) and during whisker stimulation followed by layer V photoactivation (blue bar, L_1). Blue light was presented at ~ 0 ms with respect to the time of onset of the whisker-evoked response. All PREs in a time window from -40 ms to +100 ms from the onset of the whisker stimulation were considered. **(M)** Mean values of absolute error of PREs in whisker stimulation trials and in trials in which whisker stimulation was paired with layer V photostimulation. n = 167 PREs (no layer V activation) and n = 161 PREs (layer V activation at ~ 0 ms); Mann-Whitney test. **(N)** Average values of the absolute error of PREs across animals for whisker stimulation and for concurrent whisker stimulation and layer V photoactivation. n = 4 mice, Paired Student's *t*-test. **(O)** Same as in (K) for layer V photoinhibition. **(P-P_1)** Same as in (L-L_1) for layer V photosupression. **(Q)** Same as in (M) for layer V photoinhibition. n = 132 PREs (no layer V inactivation), n = 190 PREs (layer V inactivation); Mann-Whitney test. **(R)** Same as in (N) for layer V photosuppression. n = 6 mice, Paired Student's *t*-test.

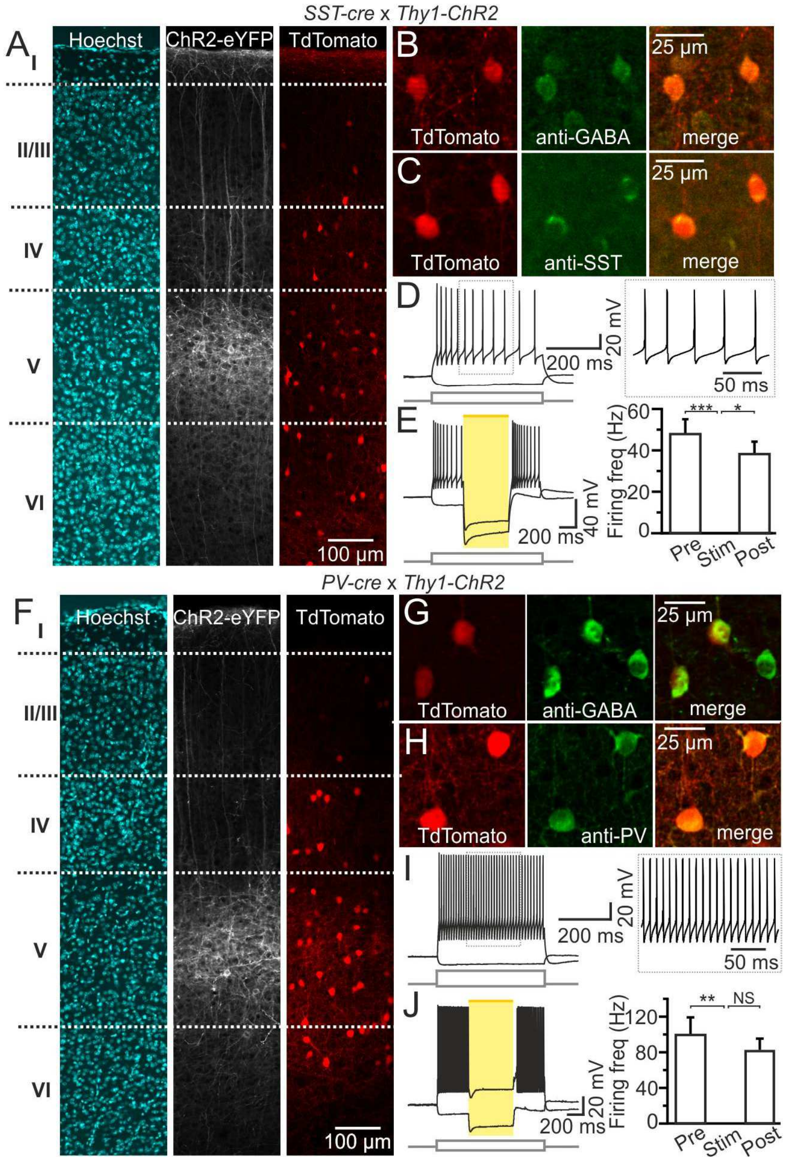

Figure S5. Histological and functional characterization of injected double transgenic *SST-cre* x *Thy1-ChR2* mice and *PV-cre* x *Thy1-ChR2* mice. Related to Figure 7. (A) Confocal images showing a coronal section of the primary somatosensory cortex of a double transgenic mouse *SST-cre* x *Thy1-ChR2* which was injected with AAV transducing TdTomato. Left: the boundaries of cortical layers (dashed lines) were identified on the basis of cellular density using Hoechst staining. Middle and right: Confocal images showing ChR2-eYFP (middle) and TdTomato (right) expression. Note that the somata of ChR2-expressing cells are confined to infragranular layers while TdTomato-positive cells are distributed across cortical layers. Data were pooled from 3 animals (3 sections *per* animal). **(B)** Left: confocal image of cortical section from a *SST-cre* x *Thy1-ChR2* mouse which was injected with AAV transducing TdTomato. Middle: the section was immunostained against GABA (anti-GABA). Right: the two images on the left are shown merged. **(C)** Same as in (B) for a section immunostained against somatostatin (anti-SST). **(D)** *In vitro* current-clamp recordings from a TdTomato-positive cell in a *SST-cre* x *Thy1-ChR2* mouse which was injected with AAVs coding for TdTomato and Arch. Current injections: -100, +250 pA. **(E)** Left: recording from the same neuron as in (D) during concurrent current injections (-100, +250 pA) and Arch photoactivation (yellow bar). Right: average firing frequency before (pre), during (stim), and after (post) yellow light stimulation in cells expressing the inhibitory opsin Arch or Halo. n = 11 cells located in layer V and II/III. Friedman test **(F)** Same as in (A) for a coronal section of the primary somatosensory cortex of a double transgenic mouse *PV-cre* x *Thy1-ChR2* which was injected with AAV transducing TdTomato. Data were pooled from 3 animals (3 sections *per* animal). **(G)** Same as in (B) for a cortical section from a *PV-cre* x *Thy1-ChR2* mouse which was injected with AAV transducing TdTomato. **(H)** Same as in (C) for a section which was immunostained against parvalbumin (anti-PV). **(I)** *In vitro* current-clamp recordings from a TdTomato-positive cell in a *PV-cre* x *Thy1-ChR2* mouse which was injected with AAVs coding for TdTomato and Arch. Current injections: -100, +600 pA. **(J)** Left:

recording from the same neuron as in (I) during concurrent current injections (-100, +450 pA) and Arch photoactivation (yellow bar). Right: average firing frequency before (pre), during (stim), and after (post) yellow light stimulation in cells expressing Arch or Halo. n = 6 cells located in either layer V or II/III. Friedman test.

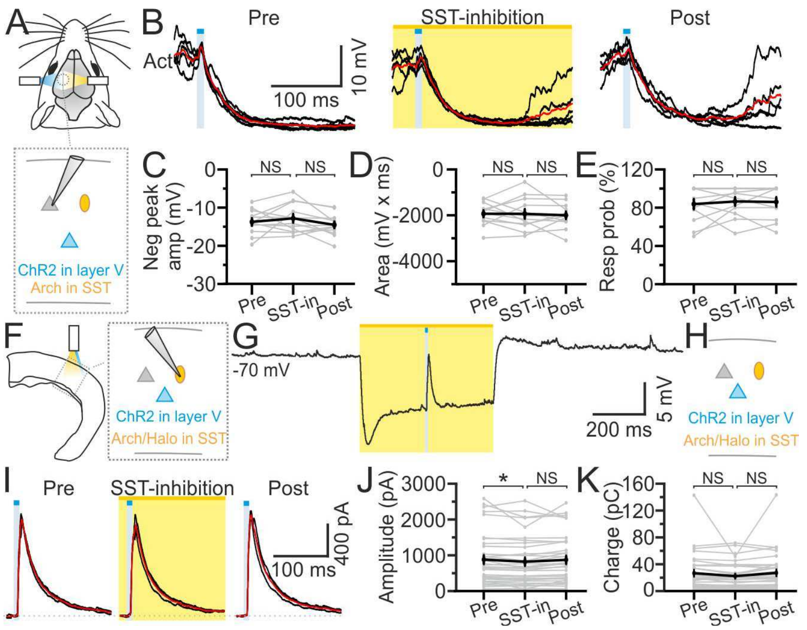

Figure S6. Inhibition of somatostatin (SST) interneurons has minor effects on cortical responses to layer V stimulation. Related to Figure 7. (A) *In vivo* recordings from both superficial and deep principal ChR2-negative cells (grey triangle) during concurrent stimulation of layer V principal neurons (blue triangle) and inhibition of SST interneurons (yellow oval). **(B)** Representative patch-clamp current-clamp recordings from a ChR2-negative principal neuron *in vivo* to blue light stimulation alone (Pre and Post) and to combined blue and yellow light stimulation (SST-inhibition) during an activated state. **(C-E)** Negative peak amplitude (C), area (D), and response probability (E) of light-evoked membrane potential responses under the conditions shown in (B). n = 12 cells from 6 animals. One-way repeated measures ANOVA. **(F)** Schematic of the experimental configuration in brain slices. **(G)** Representative trace from an Arch-expressing SST interneuron showing efficient cell hyperpolarization following yellow light illumination (yellow bar). Blue light pulse (blue bar) delivered during photoinhibition resulted in subthreshold EPSP. **(H)** Schematic of the recordings in brain slices from either ChR2-negative (grey triangle) or ChR2-positive (blue triangle) neurons. ChR2-negative cells were located both in layer II/III and V. **(I)** IPSCs evoked in recorded cells by layer V stimulation before (Pre), during (SST-inhibition), and after (Post) photoinhibition of SST interneurons. **(J-K)** Amplitude (J) and charge transfer (K) of IPSCs under the different experimental conditions. n = 35 cells. One-way repeated measures ANOVA.

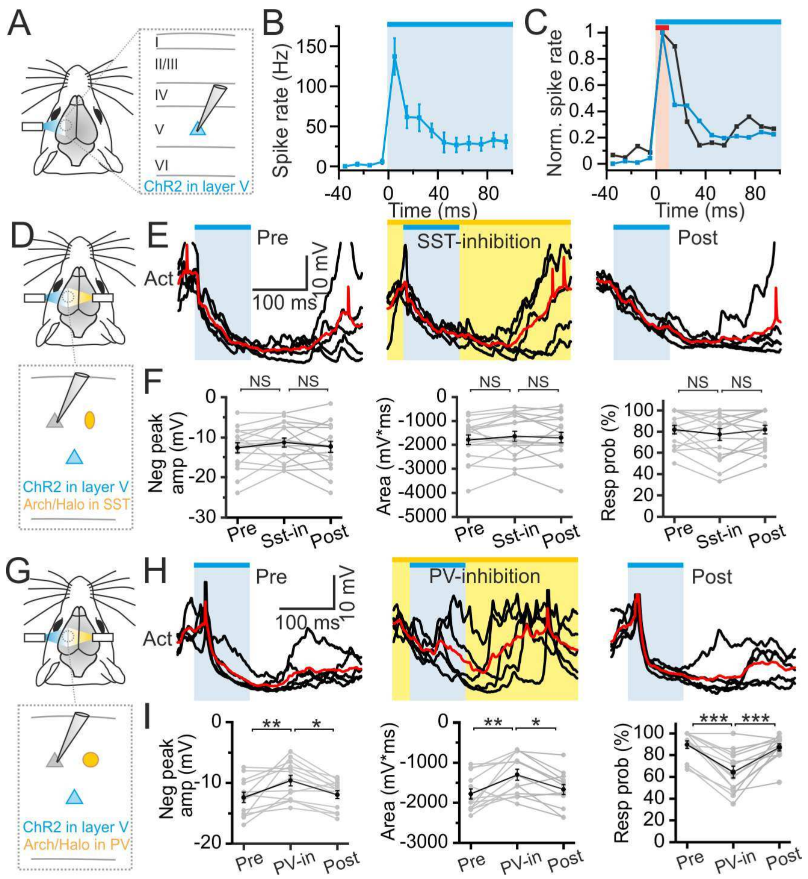

Figure S7. Effects of photoinhibition of SST or PV interneurons on cortical responses to prolonged layer V stimulation. Related to Figure 7. (A) Schematic of the experimental configuration. Juxtasomal recordings of layer V Thy1-ChR2-positive cells were performed in anesthetized animals during blue light stimulation (stimulus duration: 100 ms). **(B)** Average peri-stimulus time histogram (bin: 10 ms) of layer V ChR2-positive neurons during blue light stimulation (stimulus duration: 100 ms). n = 10 cells from 8 animals. **(C)** Normalized average peri-stimulus time histogram for cells reported in panel B (blue line) and in Figure 1E (black line) showing similar temporal profile of layer V spike activity during layer V optogenetic stimulation (blue bar) and during whisker stimulation (red bar). **(D)** *In vivo* recordings of ChR2-negative principal neurons (grey triangle) were performed in both superficial and deep layers during concurrent photoactivation of a subpopulation of layer V principal neurons (blue triangle) and photoinhibition of SST interneurons (yellow oval). **(E)** Five representative current-clamp patch-clamp traces (black) and their mean (red) from a ChR2-negative principal neuron *in vivo* during optogenetic stimulation of layer V with a prolonged blue light stimulus (Pre and Post, stimulus duration: 100 ms) and during combined blue and yellow light stimulation (SST-inhibition) during an activated state. **(F)** Negative peak amplitude (left), area (middle), and response probability (right) of light-evoked membrane potential responses under the three experimental conditions shown in (E). n = 17 cells from 8 animals. One-way repeated measures ANOVA. **(G)** Same as in (D) for photoinhibition of PV interneurons (yellow oval). **(H)** Same as in (E) for PV-inhibition. **(I)** Same as in (F) for PV-inhibition. n = 13 cells from 7 animals. One-way repeated measures ANOVA.

Laser power (mW)	Subthreshold latency (ms)	# of APs *per* stimulus	Time to 1st spike (ms) [# cells displaying APs]	Time to 2nd spike (ms) [# cells displaying APs]	Time to 3rd spike (ms) [# cells displaying APs]	# of recorded cells
18	0.19 ± 0.03	1.33 ± 0.19	3.3 ± 0.5 [n = 11]	11.1 ± 0.8 [n = 5]	N/A	12
13	0.20 ± 0.03	1.24 ± 0.18	3.5 ± 0.6 [n = 10]	12.7 ± 1.2 [n = 5]	N/A	11
7	0.22 ± 0.04	1.21 ± 0.17	4.4 ± 0.9 [n = 11]	11.9 ± 1.4 [n = 3]	24.0 ± 0.0 [n = 1]	11
4.6	0.26 ± 0.03	1.17 ± 0.17	4.6 ± 0.7 [n = 11]	11.6 ± 1.0 [n = 3]	N/A	12
1.8	0.51 ± 0.10	0.97 ± 0.25	6.4 ± 1.0 [n = 8]	12.8 ± 2.7 [n = 2]	20.9 ± 0.0 [n = 1]	11
0.14	1.14 ± 0.17	0.42 ± 0.20	8.4 ± 1.3 [n = 4]	12.7 ± 0.00 [n = 1]	N/A	11

Table S1. Light-evoked responses in ChR2-positive neurons in brain slices. Related to Figure 1 and Figure S1. Layer V ChR2-positive cells were recorded in patch-clamp current-clamp mode in isolated cortical slices. Blue light of different intensities (laser power) was shined on the sample for 10 ms through a fiber optic. ChR2-positive cells were characterized by subthreshold latency <1 ms at laser power > 1 mW (at the fiber exit, see STAR Methods).

	Subthreshold latency in the Res. state (ms)	Subthreshold latency in the Act. state (ms)	Significance
Exc. LII/III (n = 12)	2.32 ± 0.17	2.44 ± 0.13	* *vs* LII/III awake in Res. state<br>* vs LV in Act. state<br>* vs LVI in Act. state<br>* *vs* LII/III wide-field DMD in Act. state<br>*** *vs* LII/III awake in Act. state
Exc. LIV (n = 4)	2.79 ± 0.38	2.81 ± 0.26	* *vs* LII/III awake in Res. state<br>** vs LV in Act. state<br>* *vs* LII/III awake in Act. state
Exc. LV (n = 13)	2.14 ± 0.16	2.03 ± 0.12	* *vs* LII/III in Act. state<br>** *vs* LIV in Act. state<br>* *vs* LII/III wide-field DMD in Act. state
Exc. LVI (n = 12)	2.17 ± 0.23	2.01 ± 0.22	* *vs* LII/III in Act. state<br>* *vs* LII/III patterned DMD in Act. state<br>* *vs* LII/III wide-field DMD in Act. state
Exc. LII/III (n = 6) Patterned DMD	2.73 ± 0.30	3.02 ± 0.47	* *vs* LII/III awake in Res. state<br>* *vs* LVI in Act. state<br>* *vs* LII/III awake in Act. state
Exc. LII/III (n = 6) Wide-field DMD	2.67 ± 0.34	3.19 ± 0.42	* *vs* LII/III awake in Res. state<br>* *vs* LII/III in Act. state<br>* *vs* LV in Act. state<br>* *vs* LVI in Act. state<br>* *vs* LII/III awake in Act. state
Exc LII/III (n = 7) Awake	1.84 ± 0.15	1.75 ± 0.06	*** *vs* LII/III in Act. state<br>* *vs* LIV in Act. state<br>* *vs* LII/III DMD in Act. state<br>* *vs* LII/III wide-field DMD in Act. state

Table S2. Light-evoked responses of ChR2-negative cells *in vivo*. Related to Figure 1 and Figures S2 and S3. Principal ChR2-negative cells were recorded in current-clamp patch-clamp mode *in vivo*. Student's *t*-test was used for statistical comparison between two given classes of cells. When unequal variances were detected, the Welch's correction was used. Mann-Whitney test was applied in the case of non-parametric data.

	Laser power: 18-14 mW<br>Vm = -70 mV	Laser power: 4.6-5 mW<br>Vm = -70 mV	Laser power: 18-14 mW<br>Vm = -50 mV	Laser power: 4.6-5 mW<br>Vm = -50 mV
**Exc. LV (n=12)**				
Subthreshold latency (ms)	2.29 ± 0.15	3.05 ± 0.56	2.57 ± 0.21	3.18 ± 0.53
# of cells with APs	0	0	0	1
# of APs *per* stimulus	0	0	0	0.38 ± 0.00<br>(n = 1)
**Exc. LII/III (n=10)**				
Subthreshold latency (ms)	3.77 ± 0.18	4.79 ± 0.38	3.86 ± 0.24	5.62 ± 0.78
# of cells with APs	6	0	8	2
# of APs *per* stimulus	0.85 ± 0.15<br>(n = 6)	0	0.90 ± 0.10<br>(n = 8)	1 ± 0<br>(n = 2)
**Exc. LIV (n=10)**				
Subthreshold latency (ms)	2.44 ± 0.12	2.54 ± 0.14	2.35 ± 0.18	2.78 ± 0.19
# of cells with APs	0	0	2	0
# of APs *per* stimulus	0	0	0.27 ± 0.13<br>(n = 2)	0

Table S3. Light-evoked responses in principal ChR2-negative neurons in brain slices. Related to Figure 5. Layer V, II/III, and IV principal ChR2-negative cells were recorded in patch-clamp current-clamp mode in isolated cortical slices. Blue light of different intensities was shined for 10 ms through a fiber optic. Laser power used in Figure 5 was 4.6-5 mW.

	Subthreshold latency (ms)	Significance
FS LV (n = 9)	2.09 ± 0.21	*** *vs* NFS LII/III
NFS LV (n = 11)	2.66 ± 0.21	*** *vs* NFS LII/III<br>* *vs* FS LIV
FS LII/III (n = 6)	2.73 ± 0.25	** *vs* NFS LII/III<br>* *vs* FS LIV
NFS LII/III (n = 14)	4.62 ± 0.37	*** *vs* FS LV<br>*** *vs* NFS LV<br>** *vs* FS LII/III<br>*** *vs* FS LIV
FS LIV (n = 8)	1.94 ± 0.16	* *vs* NFS LV<br>* *vs* FS LII/III<br>*** *vs* NFS LII/III

Table S4. Subthreshold latencies of light-evoked responses in interneurons recorded in brain slices. Related to Figure 6. Interneurons were recorded in patch-clamp current-clamp mode in isolated cortical slices. Blue light (power: 4.6-5 mW) was shined for 10 ms through a fiber optic. Student's *t*-test was used for statistical comparison among two given classes of cells. When unequal variances were detected, the Welch's correction was used. Mann-Whitney test was applied in the case of non-parametric data.

	Exc. LV (n = 12)	Exc. LII/III (n = 10)	Exc. LIV (n = 10)	FS LV (n = 9)	NFS LV (n = 11)	FS LII/III (n = 6)	NFS LII/III (n = 14)	FS LIV (n = 8)
V rest (mV)	-69 ± 2	-78 ± 2	-72 ± 2	-70 ± 2	-65 ± 2	-67 ± 3	-66 ± 3	-68 ± 1
Input Resistance (MΩ)	154 ± 15	125 ± 14	129 ± 19	110 ± 16	175 ± 24	71 ± 8	212 ± 22	65 ± 8
Rheobase (pA)	121 ± 14	140 ± 19	145 ± 34	300 ± 42	168 ± 38	442 ± 40	118 ± 15	419 ± 38
Firing frequency max (Hz)	40 ± 3	35 ± 3	40 ± 2	197 ± 19	66 ± 10	148 ± 15	53 ± 7	189 ± 13
First spike threshold (mV)	-39 ± 1	-37 ± 1	-41 ± 1	-42 ± 2	-42 ± 2	-37 ± 2	-40 ± 7	-39 ± 2
First spike amplitude (mV)	75 ± 4	71 ± 3	80 ± 2	69 ± 4	68 ± 5	51 ± 6	74 ± 3	61 ± 3
First spike duration (ms)	1.36 ± 0.14	1.79 ± 0.08	1.14 ± 0.04	0.43 ± 0.05	0.82 ± 0.06	0.55 ± 0.05	0.99 ± 0.10	0.40 ± 0.02
First spike AHP (mV)	14 ± 1	14 ± 1	13 ± 2	22 ± 1	13 ± 2	18 ± 3	12 ± 1	19 ± 1
ISI_1/ISI_n	0.34 ± 0.06	0.32 ± 0.03	0.45 ± 0.03	0.66 ± 0.03	0.41 ± 0.06	0.67 ± 0.06	0.31 ± 0.04	0.59 ± 0.04

Table S5. Biophysical properties of recorded neurons in brain slice preparation. Related to Figures 5 and 6. AHP, after hyperpolarization; ISI_1, inter spike interval between the first pair of discharged spikes; ISI_n, inter spike interval between the last pair of discharged spikes.
